# Global Text Mining and Development of Pharmacogenomic Knowledge Resource for Precision Medicine

**DOI:** 10.3389/fphar.2019.00839

**Published:** 2019-08-07

**Authors:** Debleena Guin, Jyoti Rani, Priyanka Singh, Sandeep Grover, Shivangi Bora, Puneet Talwar, Muthusamy Karthikeyan, K Satyamoorthy, C Adithan, S Ramachandran, Luciano Saso, Yasha Hasija, Ritushree Kukreti

**Affiliations:** ^1^Genomics and Molecular Medicine Unit, Council of Scientific and Industrial Research (CSIR)—Institute of Genomics and Integrative Biology (IGIB), New Delhi, India; ^2^Department of Biotechnology, Delhi Technological University, Delhi, India; ^3^Department of Biomedical Sciences, Acharya Narayan Dev College, University of Delhi, New Delhi, India; ^4^G N Ramachandran Knowledge Centre, Council of Scientific and Industrial Research (CSIR)—Institute of Genomics and Integrative Biology (IGIB), New Delhi, India; ^5^Academy of Scientific & Innovative Research (AcSIR), New Delhi, India; ^6^Institute of Medical Biometry and Statistics, University of Lübeck University Medical Center Schleswig-Holstein - Campus Lübeck, Lübeck, Germany; ^7^Institute of Human Behaviour and Allied Sciences, Delhi, India; ^8^Department of Bioinformatics, Alagappa University, Karaikudi, India; ^9^School of Life Sciences, Manipal University, Manipal, India; ^10^Central Inter-Disciplinary Research Facility (CIDRF), Pondicherry, India; ^11^Department of Physiology and Pharmacology “Vittorio Erspamer,” Sapienza University of Rome, Rome, Italy

**Keywords:** text mining, precision medicine, disease–drug–gene–mutation relationship, pharmacogenomic markers, pharmacogenomic knowledgebase

## Abstract

Understanding patients’ genomic variations and their effect in protecting or predisposing them to drug response phenotypes is important for providing personalized healthcare. Several studies have manually curated such genotype–phenotype relationships into organized databases from clinical trial data or published literature. However, there are no text mining tools available to extract high-accuracy information from such existing knowledge. In this work, we used a semiautomated text mining approach to retrieve a complete pharmacogenomic (PGx) resource integrating disease–drug–gene-polymorphism relationships to derive a global perspective for ease in therapeutic approaches. We used an R package, pubmed.mineR, to automatically retrieve PGx-related literature. We identified 1,753 disease types, and 666 drugs, associated with 4,132 genes and 33,942 polymorphisms collated from 180,088 publications. With further manual curation, we obtained a total of 2,304 PGx relationships. We evaluated our approach by performance (precision = 0.806) with benchmark datasets like Pharmacogenomic Knowledgebase (PharmGKB) (0.904), Online Mendelian Inheritance in Man (OMIM) (0.600), and The Comparative Toxicogenomics Database (CTD) (0.729). We validated our study by comparing our results with 362 commercially used the US- Food and drug administration (FDA)-approved drug labeling biomarkers. Of the 2,304 PGx relationships identified, 127 belonged to the FDA list of 362 approved pharmacogenomic markers, indicating that our semiautomated text mining approach may reveal significant PGx information with markers for drug response prediction. In addition, it is a scalable and state-of-art approach in curation for PGx clinical utility.

## Introduction

With advancements in high-throughput genomic technologies since the past decade, the focus of pharmacogenomic (PGx) research has moved from candidate gene studies to large-scale clinical PGx. Identifying important drug response genes is critical in PGx. A given drug may have pharmacogenes—genes important for its pharmacology—that are involved in its pharmacokinetics fate or pharmacodynamics action (Walker, 2004). However, to clearly elucidate the role of these genes for any particular drug takes years of research, and abundant articles are published in various perspectives. This delay impedes our ability to identify, evaluate, and use genetics to optimize drug selection and dosing with minimal toxicity (Ventola, 2013).

Generally, we explore the answers to such questions in publications describing the disease–drug–gene relationships of interest in a particular population. Such relationships of clinical importance for drug dosing and administration must be interpreted as a priority. Analyzing such related data from the literature, we need to rapidly identify and develop high-throughput, accurate, and population-specific genetic polymorphisms that correlate with drug response. Such genetic considerations can be expected to be important in diagnosis, treatment, and prevention. Both clinical and research communities have placed emphasis on identifying PGx relationships. Several databases employ manual curation of biomedical literature to provide comprehensive coverage of such disease or drug-related genetic association relationships in humans. Some of them are Online Mendelian Inheritance in Man (OMIM) (Amberger et al., 2009), Human Gene Mutation Database (HGMD) (Stenson et al., 2009), the Comparative Toxicogenomics Database (CTD) (Davis et al., 2016), Genetics Home Reference (GHR) (http://ghr.nlm.nih.gov/) (National Library of Medicine (US), Genetics Home Reference 2013), and the Pharmacogenomics Knowledgebase (PharmGKB) (Whirl-Carrillo et al., 2012). Despite such focused approach to capture valuable PGx information in biomedical databases, much of this information still remains inaccessible in the unstructured text of biomedical publications.

A fully automated PGx relationship curation system to retrieve clinically relevant information is still far-fetched (Singhal et al., 2016). Therefore, advanced computational approaches with statistical evaluation can reduce manual efforts to curate important PGx relationships from available literature. The various sources of experimental noise reported in different articles result in a number of important genes or polymorphisms being overlooked in the biomedical text. Therefore, the recent efforts in (semi-)automated approaches facilitate automated extraction with manual curation of relationships for high quality are critical (Garten et al., 2010). Hence, in this paper, we propose an end-to-end semiautomated approach for the extraction of disease–drug–gene–polymorphism relationships in different global populations from biomedical literature. If an article mentions a drug and a genetic association, these articles are screened for their relevance in PGx in context to any drug response. The biological entities (e.g., disease, drug, gene, genetic variant) obtained by automated extraction were normalized with standard available datasets to exclude the ambiguities in publications. Furthermore, for performance evaluation, we compared our proposed pipeline with databases like OMIM (Amberger et al., 2009), CTD (Davis et al., 2016), and PharmGKB (Whirl-Carrillo et al., 2012) to assess the sensitivity and specificity of the disease-drug–gene–polymorphism relationships. We also calculated the accuracy of each relationship obtained and compared their occurrence within the three datasets. We conducted a validation study by comparing our result with commercially used FDA-approved drug labeling biomarkers (https://www.fda.gov/drugs/science-research-drugs/table-pharmacogenomic-biomarkers-drug-labeling) (FDA, 2018). The final result of this approach is the disease–drugs–gene–variant mined from the literature published to date. The final PGx relations extracted were also prioritized for significance in clinical application. The key feature of the study is the use of text mining to tabulate the most important PGx information related to disease or given drugs by studying its variability and impact on individuals, which can be used for future clinical administration.

## Methods

### Dataset Used

**Figure 1:**
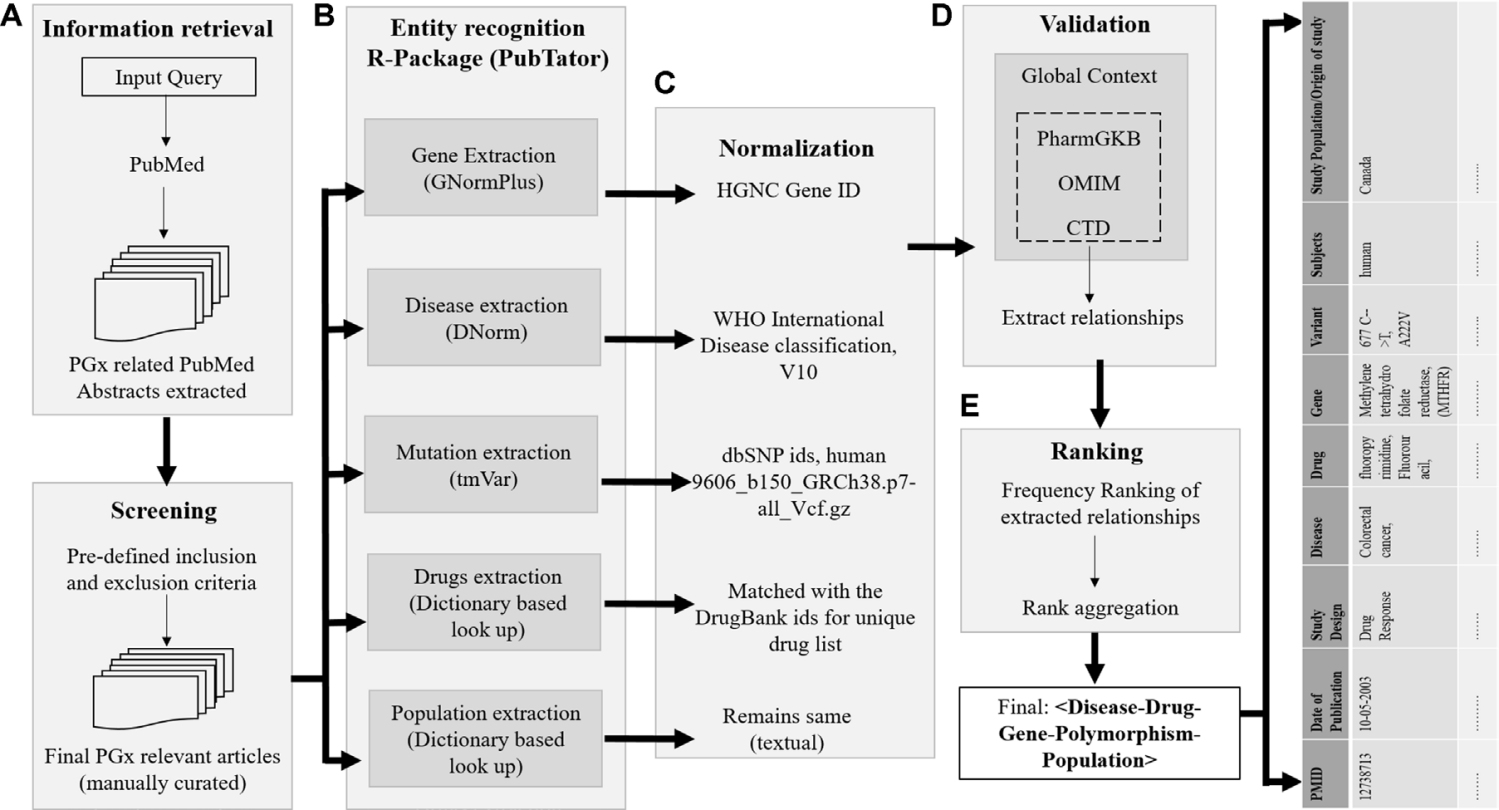
Overview of the proposed approach. The process of retrieving evidence-based sentences from PubMed abstracts using pubmed.mineR includes: **(A)** information retrieval, **(B)** entity recognition, **(C)** normalization, **(D)** validation, and **(E)** data integration and ranking. The final list of relationships of disease–drug–gene–polymorphism is tabulated population-wise.

The schematic representation of the overall study architecture is shown in **Figure 1** and can be summarized in the following steps: step 1, build a corpus of PGx and related abstracts fetched from PubMed using the Medical Subject Headings (MeSH) query; step 2, identify all biological entities in these PubMed abstracts (diseases, genes, drugs, polymorphisms, and populations); step 3, normalization of the obtained entities; step 4, validation with available dataset(s) in global context; and step 5, evaluation of extracted data and ranking of PGx relationships. This process results in a list of all PMIDs aligned PGx relationships of the form < disease–drug–gene–polymorphism>.

The details of each step are described in the following paragraphs. We used the in-house built R package, pubmed.mineR (Rani et al., 2015), to extract the PGx relationships from the corpus. This package mines a given literature corpus without dependency on other packages for information extraction. Many such online text-mining algorithms are available like GoPubMed (Doms and Schroeder, 2005) and PolySearch2 (Liu et al., 2015). These available algorithms perform direct extraction of sentences from PubMed abstracts and highlight genes, disease, and species for easy reading through texts. These algorithms have limitations as are rigid in extracting predesigned concept lines and are not available in open source. Keeping in mind the limitations of the existing systems, and combining their advantages, pubmed.mineR offers user flexibility to expand the user capabilities for executing multifaceted approaches. For example, on retrieving all the genes studied with warfarin drug response association from the literature, Polysearch2 could retrieve only six MEDLINE citations with CYP p450 gene, and PubTator retrieved ∼20,000 articles. However, warfarin had ∼500 publications in PharmGKB with different genes associated. It facilitates the extraction of terms and their contexts, gene recognition, association between terms and between genes, including cross-associations, and hunting for key evidences for proof of associations.

**Step 1: Information retrieval.** The dataset used in this pipeline includes PubMed articles only. Our PubMed query was formed using MEDLINE’s MeSH and included terms “inter-individual variability,” “pharmacogenomics,” “pharmacogenetics,” and “drug response.” We downloaded all PubMed citations that were human studies with an abstract available. The citations were downloaded in CSV format using the “e-utilities” interface provided by NCBI. The corpus of articles for this literature mining, containing pharmacogenetics and related articles, was thus created using the R package, pubmed.mineR.

**Step 2: Entity recognition.** For each PMID, the annotation results of all the biomedical entities mentioned in the abstract, i.e., disease, drugs, gene, and mutation, were obtained using PubTator (Wei et al., 2013). In PubTator, the four biological entities—disease, chemical, gene, and related mutation annotations—were extracted by DNorm (Leaman et al., 2013), dictionary-based lookup approach, GNormPlus (Wei et al., 2015), and tmVar (Wei et al., 2013), respectively. The population of each study was extracted independently using a dictionary-based content search by the pubmed.mineR package. Since we focused on extracting PGx association, we retained only those abstracts that had at least one drug mention, with one gene mention, or any genetic variant mentioned.

**Step 3: Normalization.** The annotated articles were then passed through several filters, with each entity normalized to reduce false positives and ambiguity. Gene mentioning normalization was initially assessed based on the lexica provided with PubTator dataset, i.e., GNormPlus. The annotated genes retrieved from our PGx corpus were further matched with orthographical variations used by the authors to generate a standard expression that is identical to HGNC gene names (Yates et al., 2017). The gene entities that did not match the HGNC lexicon were preprocessed to find enumeration of potential names and rematched. All the unmatched entities were ruled out, based on abbreviated names, unconventional names, unspecified names, and other disambiguation. The baseline system implemented for disease normalization used dictionary lookup method using parent disease terms from International Classification of Diseases (ICD-10): version 2016 (Organisation, 2004). All the arbitrary terms referring to a symptom or any consequence of a disease/syndrome which is not a disease in itself (has not been classified as a disease by ICD) were excluded, as they resulted in a high error percentage. Initially, the dictionary lookup method was configured to identify exact matches. However, in case of no exact match, a partial match with the parent disease concept was accepted. Drugs were matched with DrugBank IDs for unique drugs (Wishart et al., 2008). In case any other names of the drug (e.g., its chemical name, brand name, etc.) or the drug metabolite, chemical names associated with any gene/polymorphism were identified, the entities failed to normalize and were consequently excluded. Finally, the genetic polymorphisms related to the selected genes were retrieved in the final subcorpus and were matched according to their annotation in dbSNP IDs, human (ftp site:ftp://ftp.ncbi.nlm.nih.gov/snp/organisms/human_9606/genotype_by_gene/).

**Step 4: Validation.** Validation was carried out independently in two subtasks: 1) all the biological entities obtained were validated for their presence in any of the three benchmark datasets, and 2) the PGx relationships obtained were cross-validated with the gold standard, PharmGKB. The benchmark datasets used to analyze this proposed semiautomated approach output were OMIM, CTD, and the gold standard, PharmGKB. We used the ground truth labels of the PharmGKB database to validate the PGx relationships that were retrieved after mining the proposed approach, as OMIM focuses on disease–gene variant association and not on the genetic relevance with regard to drug intervention. Similarly, CTD database manually curates chemical–gene, chemical–disease, and gene–disease relationships, but genetic variant was not annotated in this database. A detailed statistical evaluation was conducted to assess the biological entities extracted as an output from this pipeline individually, as well as in relations with any other entity. To evaluate this, we compared the performance of the system to that obtained from OMIM, CTD, and PharmGKB and measured the concordance of the data obtained. This is one of the methods that we implemented to evaluate our findings. However, this may not be the only exhaustive resource for this validation. A circumstantial error analysis was carried out in terms of specificity, sensitivity, accuracy, precision, recall, and F measure. We further validated the entities for their presence in the three benchmark datasets; however, the PGx relationship matched using PharmGKB was considered the *gold standard*. Output from our approach is the *test data*. The true positive (TP) data are those which are present both in *gold standard* and *test data*, and true negative (TN) are data absent in both *gold standard* and *test dataset*. False positive (FP) is absent in gold standard and present in test data, and false negative (FN) is the number of correct, incorrect, and missed associations extracted by the system in comparison with the gold standard, respectively.

**Step 5: Ranking of the PGx relationships.** We report the results in terms of specificity, sensitivity, accuracy, precision, recall, and F measure. Let TP, FP, and FN be the number of correct, incorrect, and missed associations extracted by the system in comparison with the gold standard, respectively. The four candidate lists of genes obtained from our study, OMIM, CTD, and PharmGKB were combined to develop a unique list of PGx relationships from each disease–drug–gene triplet. All relationships with a frequency greater than 10 (*f* > 10) that occurred only in our framework were appended directly to the end of the consolidated list. A consensus of *f* > 10 has been decided by the authors, with the convention that lower than 10 articles may have been published as random co-occurrences or without any unidirectional scientific evidence. Hence, the papers with <10 may not be of significance. By doing so, the genes extracted from these datasets were assumed to be more relevant than those extracted from our pipeline. This is based on our observation that these datasets are manually curated and annotated with validated results with low noise, hence minimal FP genes. The gene names that overlapped between our approach and that of these datasets were found to be of prime importance, and their ranks need to be aggregated. We simply raised their rank order for such genes based on the number of occurrence in these three datasets. Finally, on stringent curation, 2,304 PGx relationships were obtained and validated. These relationships were compared with commercially used FDA-approved drug labeling biomarkers. Of the 2,304 markers obtained and validated, 127 were common with FDA-approved pharmacogenomic markers. This marked the reliability of the outcome of our pipeline. In addition, the remaining PGx relationships suggest that although they are not included in PharmGKB, they are of prime clinically importance.

## Results

The result section is split into sections based on the different steps used in our approach to extract the PGx relationship as disease–drug–gene–mutation. The overall architecture of the system framework and the obtained results in each step are represented in **Supplementary Figure 1**. Later, we evaluated the biological entities by normalizing them against the benchmark datasets, and then estimating the overall performance (precision in terms of identifying correct information with respect to disease, drug, and gene association) of our approach when compared to the three datasets. Upon validating, by comparing with the commercially available FDA-approved biomarkers for drug labeling, we ultimately present a resource of significantly enriched PGx-specific relationships as disease–drug–gene–polymorphism across populations to optimize therapeutic interventions.

### Dataset Extraction

A search for the “Pharmacogenomic”-related MeSH descriptor retrieved 633,074 abstracts, of which 518,529 were selected based on original articles only. Nonhuman studies were further excluded (88,209). The remaining citations (430,320) were then screened for the entities mentioned in the abstract. Articles with empty disease, drug, and gene fields were excluded (183,625). Out of the remaining 246,695 articles, seeds with no drug entity were excluded (66,607), as our search was focused on obtaining PGx-specific seeds. The debarred entries were mostly disease–gene association or *in silico* tool/database development methods. Of the 183,637 citations, 12 had positive drug association studies and were therefore included. Eventually, the entities in this penultimate corpus included 246,695 articles. Articles excluded at the final step were mostly nongenetic studies or cytogenetic studies or disease association related to confounding factors. Articles with a gene/genetic variant mentioned against a drug association were included to form the final PGx subcorpus retrieving 180,088 citations with PGx-specific association studies. Entities in these articles (tabulated in details in **Supplementary Tables 1–4**) were normalized with the respective standard datasets. These articles were then manually curated for their relation between disease-specific drugs administered, genes, genetic variants, drug phenotypes, and other semantic classes relevant to PGx.

### System Performance

We conducted two experiments to assess intrinsic performances of our approach.

1. To estimate the total and unique seeds of individual extraction components (entity recognition and normalization, relation extraction) (**Supplementary Figure 2**), all predictions were ranked based on their occurrence in the abstract to get a balanced assortment of entities (**Supplementary Table 6 and 9**). The breakdowns of TP, FP, and FN were also provided and were calculated as mentioned in **Supplementary Table 5**. The performance parameters during normalization indicated the poor dependability of the biological entities due to lack of nonstandard annotations in biomedical texts. A total of 82% of the gene names could be mapped to an HGNC gene ID. The remaining 18.14% could not be assigned an ID due to unspecific gene mentions (Hakenberg et al., 2008). This is because authors use different names for a gene, spell names in many ways, or even introduce completely new names. Similar is the case with diseases and drugs when matched with ICD v10 (Dogan et al., 2014) and DrugBank (Wishart et al., 2008), respectively (**Supplementary Table 2**). Some of the common errors were due to unrecognized gene name variations. For example, P-glycoprotein (P-gp)’ in the abstract is with the most relevant synonym of “MDR1,” whereas its official name (symbol) is ATP-binding cassette subfamily B member 1 (*ABCB1*). Such variations were either lexical, structural, orthographic, or morphologic. Most of the FNs were due to unknown syntactic variations; the recall can be further improved by future automated analysis of gene names/symbols to identify variation patterns. More often, FP were strict in the sense that they were wrong recognitions, regions of text not referring to a gene. There was disambiguation due to wrong recognitions of gene mentions from abstracts, such as “IL-1 receptor” being recognized separate from IL2R or with the whole mention of “type II IL-1 receptor.” Other examples like the mention of “src inhibitors” refers to the molecules that inhibit the proto-oncogene tyrosine-protein kinase, src, and itself is not a gene. **Supplementary Table 8** provides a detailed view of errors that cause FN or FPs, sorted by error type.
2. To estimate precision, recall, and accuracy of the extracted PGx relationships, the obtained data were compared to PharmGKB, OMIM, and CTD to estimate unique and total coverage of the output. The performance metrics of all the possible binary relations between the entities obtained from PubTator like disease–drug, disease–gene, drug–gene, drug–SNP, and gene–SNP relations are also shown in **Supplementary Tables 7 and 10**). Our estimation of precision, recall, and *F* measure of the obtained 2,304 PGx relationships are tabulated in **Table 1**.

**Table 1:** Performance comparison of pharmacogenomic (PGx) relationships obtained from our proposed pipeline with other benchmark datasets (OMIM, CTD, and PharmGKB).

Context type	TP	TN	FP	FN	Sensitivity	Specificity	Efficacy	Precision	Recall	*F* measure	Accuracy
Our pipeline with “PharmGKB”	1,509	208	254	78	82.6	86.9	88.2	0.904	0.930	0.923	89.1
Our pipeline with “OMIM”	2,225	–	79	–	78.0	77.5	81.8	0.600	0.681	0.764	59.3
Our pipeline with “CTD”	1,776	153	375	–	70.7	65.5	72.2	0.729	0.803	0.801	79.7
Our pipeline with (“PharmGKB” AND “OMIM” AND “CTD”)	1,875	102	275	75	82.3	84.4	93.3	0.896	0.852	0.828	94.7

PharmGKB corpus compared to that of our pipeline and the articles extracted in these datasets. The formulae used for calculating the accuracy of the proposed pipeline compared to the other datasets (ref. **Supplementary Table 5** for detailed analysis).

Through manual evaluation, we observed that most entities could be found in databases like 1000Genome population (Consortium, 2015). For drugs, and diseases, precision lies between 87 and 90%, respectively; here, we note that the system often marks diseases as adverse effects or any symptomatic form, so precision is low as the symptom would be common with other disease categories as well. Similarly, drugs have multiple applications in different disease types or are often used as an adjunct. *F* scores for all entities range from 84 to 92% for individual entity types (**Supplementary Table 9**). For relations, *F* score is between 65 and 84% (**Supplementary Table 10**).

### Error Analysis

To better understand the system performance, we performed error analysis on the output of the test dataset. We calculated the precision, recall, and *F* measure, respectively, using the gold standard datasets. The percentage distribution of the error obtained in each step of the analytical pipeline is illustrated in **Table 2**. Error calculations were carried out in two steps: on each entity and the PGx relationships obtained from the pipeline. Both were compared to the gold standard dataset simultaneously. Error percentage is calculated by the absolute error upon exact value multiplied by 100 (formula as in equation 1). In our data, as shown in **Table 2**, the “observed value in data” is the entity output obtained from our pipeline, and “true value in data” is that extracted from the gold standard datasets. Since our pipeline screened a large PGx corpus of 188,088 articles, it may have entities more than that in gold standard dataset like PharmGKB. Such entities co-occurring in any PGx relationship have been presented as novel evidences, which, on validation in specific population cohorts, may be taken forward for clinical annotation and marker development.

**Table 2:** Error analysis evaluation results on different types of error occurrence on the test dataset.

Sl. No.	Sources of error	True value in data	Observed value in data	Error percentage
1.	Entity detection error	633,074*	582,428	8.00%
2.	Entity absent in text	633,074*	615,650	2.75%
3.	Failure to detect entity	633,074*	609,413	3.73%
4.	Entity normalisation error			
a.	Gene normalization error	42,607	50,336	18.14%
b.	Disease normalization error	71,704	92,481	28.97%
c.	Drug normalization error	11,033	14,563	31.99%

$\text{Error percentage}=\frac{\left|\text{Observed value-True value}\right|}{\text{True value}}\times 100\;(1)$

PharmGKB has been considered as the gold standard dataset for all the comparisons. *in total PGx corpus extracted from MEDLINE. The error percentage has been calculated according to the formula 1.

#### Entity Detection Error

Error in entity obtained from the PubTator tool was also another significant source for precision error. Such errors occur due to an overlap of two different entities, spanning in the name of one of them. This error occurred when entities were present but are not detected by the tool. Consider the example of article “*High serum sclerostin levels are associated with a better outcome in haemodialysis patients*” (PMID: 26890570), GNormPlus incorrectly extracted “Sclerostin” as gene, whereas no gene has been studied to be associated with any drug response phenotype. This led to the extraction of a false association between “sclerostin” with drugs, “Alfacalcidol” and/or “Warfarin.”

#### Entities Absent in Text

The absence of entities in the text can be primarily because of two reasons: either there are no entities in the abstract or no abstracts available in PubMed; or the PubTator could not retrieve the entities mentioned due to erroneous syntax. For PMID 28398598, the authors hypothesize clinical decision support tool for PGx prescribing. Although the abstract does not mention any drug–gene association, it monitors the prescription of most commonly prescribed medications according to the developed tool (Hakenberg et al., 2012). Certainly, it justifies the need for manual curation for entity recognition from such articles. Restricting our entity recognition and processing to only biomedical abstracts is a primary limitation of this study and major reason for this error.

#### Failure to Detect Entities

Failure to detect entities is also a source of errors. Consider the article (PMID: 28220983), the clinical significance of the CNV 1q21·1 microdeletion or microduplication has been studied. However, the mutation extraction tool, tmVar, could not identify CNV as mutations.

### Entity Normalization Error

Errors in entity normalization result in precision errors. The annotation obtained from our test dataset did not match with the gold standard annotation when the genes were matched with the HGNC gene IDs (18.14%). Owing to unspecific gene mentions, it was difficult to assign a gene ID (for example, a name of a gene family, ‘‘major histocompatibility complex’’ belong to “human leukocyte antigen”) to nonhuman genes like murine or rat genes, which contributed to FP in gene mentions, and for diseases, when normalized with ICD parent disease class (28.97%) and drugs with the DrugBank IDs (31.99%). In the case of genes, the error arises due to inclusion of nongenic regions like pseudogenes, miRNA, intergenic regions, and upstream elements. For diseases, if there are multiple names, occurrence with other names might be missed when searching for a specific disease. In addition, most of these mentioned in the test dataset comprised of the parent disease category in WHO ICD v·10 (2016); however, diseases like “aneurysm,” “stroke,” and “embolism” appears under both “diseases of nervous system” as well as “diseases of the circulatory system.” Such discrepancies resulted in normalization error. When drugs obtained from the test data were normalized to the DrugBank IDs, the large amount of the error occurred as the active drug compound had synonymous chemical name or common name and different metabolites with synonyms. This resulted in a high normalization error percentage (31.99%) in the case of drugs.

#### The Absence of Annotated Pgx Relationships in Benchmark Datasets

**Figure 2:**
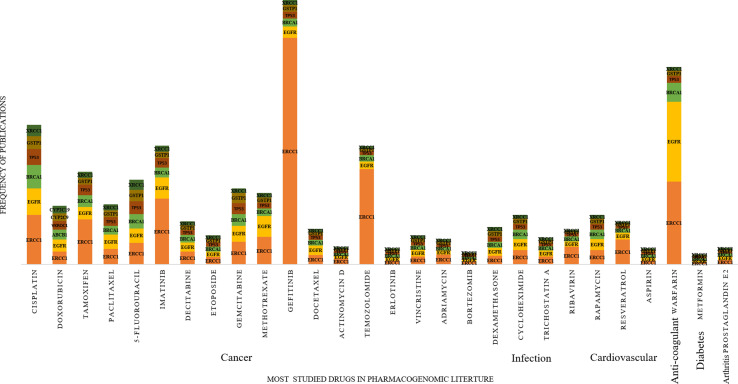
Distribution of pharmacogenomic (PGx)-specific entities obtained from 2,304 PGx relationships with at least 100 citations. In the increasing order of the most prevalent medications prescribed from left to right and the most studied genes studied for PGx association with these drugs.

**Figure 3:**
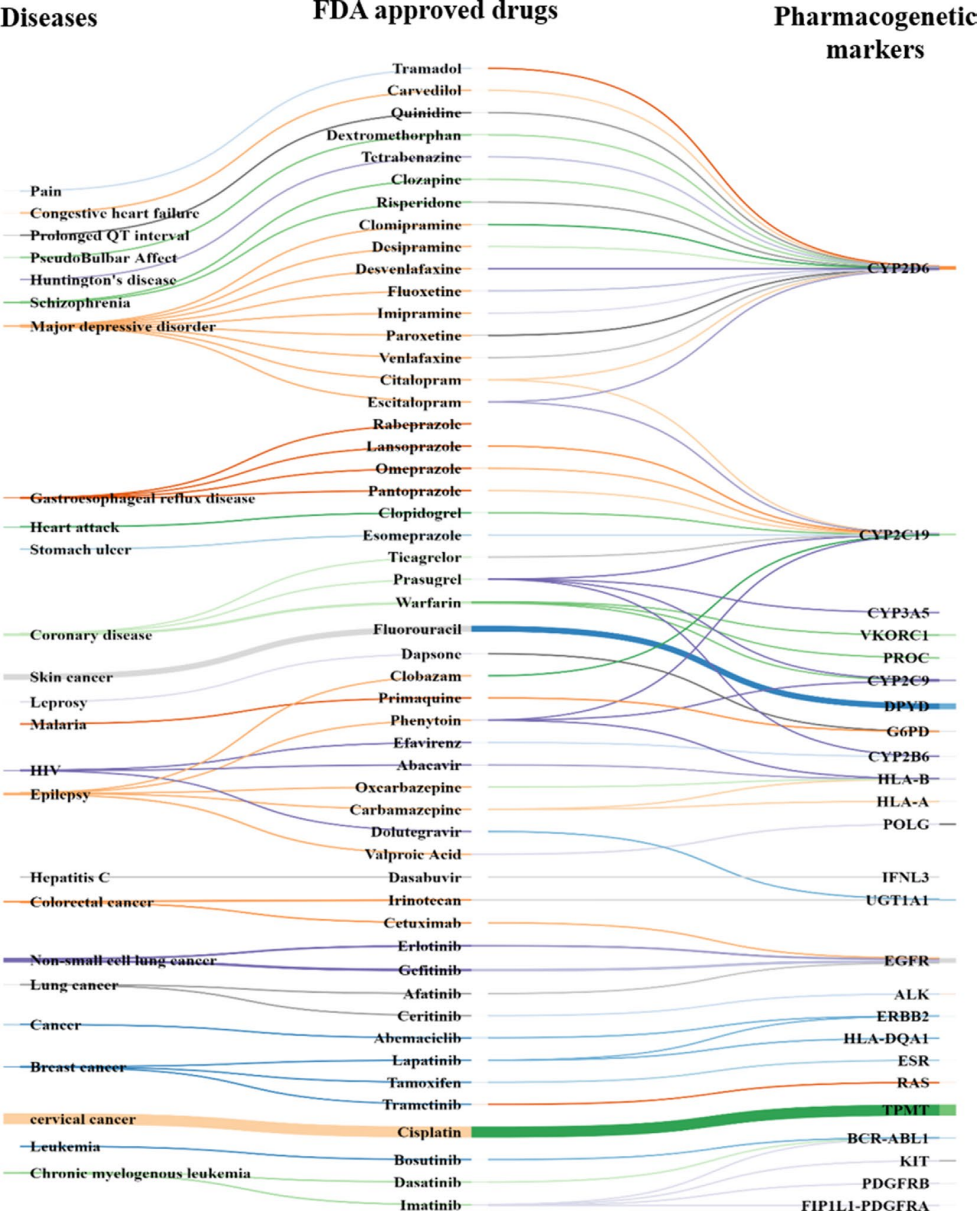
PGx-specific enriched markers other than that mentioned in PharmGKB. Disease ontology (left), FDA-approved drug (middle), and pharmacogenes (right) known (i.e., statistical association in clinical-genetics studies) to alter drug response or efficacy or lead to adverse drug response (**Supplementary Table 9**).

Comparing the predicted PGx associations from 180,088 PMIDs with PharmGKB, OMIM, and CTD, we estimated a coverage provided by our method. We found nearly 2,304 associations extracted by the system, of which 287 were not captured by PharmGKB. The system extracted the tertiary relation < neonatal hemolytic anemia–acetaminophen–GSS variants> from article 27581854. However, in PharmGKB, the clinical annotation entry related to acetaminophen contains only drug-toxicity-related binary relation excluding the disease neonatal hemolytic anemia. Such annotations are considered as additional important PGx relationships that can be considered for clinical utility with disease *per se*. The total and unique entities and relationships are described in **Supplementary Tables 6 and 7**, respectively. The additional PGx relationships has been tabulated with their detailed characterization and clinical significance in **Supplementary Table 11**. We found the highest coverage with drug–gene/variant annotations in PharmGKB (89.1%), and the lowest with OMIM (59.3%), which could be explained due to no drug association studies in OMIM. Comparing our system with CTD, we found that it could recover 79.7% of the drug–gene and disease–drug relations, but note that many of the drug–gene relations in CTD were also automatically extracted and not approved for PGx-specific relevance. Comparing our system with a total of all the three datasets revealed the highest coverage (94.7%) (**Table 1**). **Figure 2** presents the associated genes found by co-occurrence in 180,088 articles of the most widely studied drugs globally. The top 10 genes plotted for each of these drugs have at least 100 citations. Ultimately, **Figure 3** shows these additional PGx relationships pertaining to global PGx literature that are of prime clinical importance. The left nodes represent the disease class, the middle nodes the drugs, and the nodes on the right corresponds to the markers annotated to the genes pertaining the PGx relationship. Each color of the edges represents a relationship (disease–drug and/or drug–gene) for distinct visualization. The width of the edges represents the number of evidence present on each of the relationship. For example, cancer-cisplatin-*TPMT* has the most evidence followed by skin cancer-fluorouracil-*DPYD* and so on.

### A Resource for Pharmacogenomic Evaluation

We created a repository containing the 180,088 pharmacogenetically relevant articles identified by our scanning method with PGx-specific seeds associated to those articles. The result also comprises of 2,304 disease–drug–gene with their frequency of co-occurrences detected in those articles (**Supplementary Table 11**). Thus, our knowledge resource provides an overview over genetic variations implicated in drug response. This can be queried by disease or drug or gene, and it summarizes gene–drug relationships, categories of evidence, and supporting literature. The PGx evidences, with regard to drugs, can help us to determine clinical decisions. They provide a quick overview of how gene/genetic variants affect the drug response in individual patients, in specific population. We, therefore, provided a thorough categorization to classify disease–drug–gene relationships according to the type of pharmacogenetic evidence obtained from the source corpus. This growing resource needs to be tapped for clinical benefits, for drugs of pharmacogenetics significance, and is a core component of pharmacogenetic screening.

## Discussion

The dynamic knowledge about the DNA sequences and their functional consequence has ventured several exploratory research like pharmacogenomics. Investigating the DNA sequence has actually outpaced the growth in computer power for the past several years, giving rise to the new era of big data analytics. Advancements such as creating algorithms to develop computational packages to dig deeper details of DNA like associating variations in genes with clinically relevant phenotypes—disease risk, therapeutic response, and adverse effects of drugs—are expected to arise in the near future (Hansen et al., 2009). Our study attempts to measure the capability of a text mining system in semiautomatically extracting database level annotations from PubMed abstracts. Therefore, we performed retrieval of articles through an automated tool followed by manual curation for its relevance in PGx. Our evaluation of the proposed system pipeline against gold standard annotations extracted from curated database provides insight of the clinical applicability of text mining for treatment management. In context of text mining, this is the first attempt to effectively combine information from multiple sentences to extract quaternary relations between disease, drugs, genes, and polymorphism data in the global pharmacogenomic context. Our approach to link association across sentences using entity identity words resulted in substantial performance improvement. A performance of 89.1% of overall accuracy was achieved when compared with PharmGKB, which was further enhanced to 94.7% after detailed comparison with all the three curated databases. This demonstrates that our approach, to some extent, addressed the linguistic inference challenge pertaining to the use of text mining for database curation (Ravikumar et al., 2015).

First, even though our algorithm has improved precision when compared with PharmGKB and also ranking of PGx relationships of drug–gene pairs, the overall precision is still lower than the gold standard (Rubin et al., 2005). Starting from a PGx-specific seed, the algorithm implicitly classifies sentences into PGx-related or nonrelated. However, if “n” drugs and “m” genes co-occur in any PGx article, the algorithm will automatically extract all “n” and “m” possible drug–gene pairs. The extraction algorithm used was probabilistic and do not consider the syntactic relationships between drug entities and gene entities, independently in sentences. Our extraction pipeline cannot extract ready-made PGx relationships from the literature, but it can find sentences with PGx-relevant information excluding several nonspecific associations (Xu and Wang, 2013). Therefore, the emphasis on semiautomated approach has been established to construct the exact and complete knowledge resource of PGx-specific disease–drug–gene associations from published literature.

Second, the results presented in the previous section show the effectiveness of the proposed approach by performance comparison when extracted using our semiautomated method, and OMIM, CTD, and PharmGKB were used to extract correct disease–drug–gene–mutations. Quite noticeably, a large number of relationships overlapped between different combinations of the entities (e.g., disease–drug, drug–gene, drug–variant, etc.) obtained from our system and that from the standard datasets (**Table 7 of Supplementary data**). We, therefore, propose to present an independent comprehensive resource to curate important disease-related PGx relationships. We also analyzed our drug–gene pair predictions for known errors and false negatives. Known errors are PGx relationships obtained from our approach but are unrelated or nonspecific to the disease; however, they are presently associated in any of the three datasets. These are detailed in **Supplementary Tables 8–10**. It can be primarily be due to the following: a) indirect or infrequent drug mention. Such cases could be avoided by increasing the weight of target drugs, administered in a particular disease type/category, frequency in comparison to other disease mentions. b) Unrelated documents: in few cases, the documents were not directly related to the target disease. This can be improved by more comprehensive extraction of documents related to the target disease. c) Disease name ambiguity: in some cases, the DNorm tool identifies a nondisease mention as a disease, and the feature set is disturbed due to close proximity of the mutation with false disease identification. These errors occur due to ambiguous abbreviations or nonstandard notations mentioned by authors that resemble biological entity names.

Third, the entire PGx relationship extraction algorithm starts with 1,753 disease types, 666 drugs, associated with 4,132 genes, and 33,942 polymorphisms collated from 180,088 publications filtered out from 6 million MEDLINE abstracts. During the extraction and ranking process, many non-PGx-specific MEDLINE sentences were automatically excluded. Ultimately, we can rank the 2,304 human PGx relationships (**Supplementary Table 11**) according to their PGx specificity and can further improve the precision of the relationship extraction algorithm. We validated the approach by comparing our results with commercially used FDA-approved drug labeling biomarkers. Of the 362 FDA-approved pharmacogenomic markers, 127 were common, with the 2,304 markers obtained from our proposed approach (**Supplementary Table 11**).

Translational medicine revolves around the discovery of basic biological sciences and uses this research into clinical setting. Therapeutic observation can be further used for clinical development, targeted therapy, or drug repurposing. It focuses on patient care, including the creation of new diagnostics, prognostics, prevention strategies, and therapies based on pharmacogenomic discoveries for precision medicine. The emergence of translational bioinformatics spans into the development of algorithms and computational tools to derive the actual basis of molecular and cellular data with an explicit goal of affecting clinical care. This promise of translational clinical medicine is progressing with the vision of genome-guided medicine (Hauser et al., 2018).

## Limitations and Future Direction

The minimum requirement to adopt a PGx test is an association of PGx biomarker(s) and primary outcomes, replicable in a specific-population cohort mainly related to drug toxicity, or often, lack of efficacy. This should be accompanied by a clear recommendation for the drug/dose adjustment with the respective gene or its variant. Once such an association is available from systematic analysis of the relationship obtained from studies on phenotypic response and genetic variability in population-specific individuals, a PGx test can be formulated for patient evaluation. In this study, our predominant concern was to extract the PGx-specific associations from wide biomedical literature. Trimming the unwanted publications from this huge database to a sizeable number of relevant pharmacogenetics articles was obligatory. This led to the need for use of a semiautomated data mining tool that could screen retrieved articles. With such automatic extraction and manual validation, there will likely be articles that are missed due to the varied complexities of biomedical literature. In addition, ∼30% of the relationships were missed by the system due to the absence of relevant entities in the abstract. An extension to more advanced systems to overcome the additional experimental or statistical noise and retrieval of information from full text, tables, or supplementary files may result to a detailed output (Jimeno Yepes and Verspoor, 2014). Erroneous output after entity normalization was a major concern. This was due to unmatched entities filtered out based on nonstandardized, unambiguous abbreviations or unspecified names appearing in the text, which did not correspond to the standard nomenclature system. The basis of this study is to cover the broader task for PGx relationship extraction from huge corpora. The result section is divided according to the different steps of our followed approach to extract the PGx relationship as disease–drug–gene–mutation. However, this does not specifically consider genetic polymorphism and their relation with any drug response outcome since annotating genetic variant representations from evidence was in multitude. There are other significant variations in gene expression or variations linked as haplotype blocks or at protein level, which also play significant roles. These complex compound effects of gene variants, like effect of alleles or genotypes or alternative isoforms involved, were beyond the ability of the package to segregate. Therefore, disease–drug–gene relationships were only taken forward as PGx relationships in this study. Future advances in the respective domains of used tools with advanced integrated systems for multifaceted retrieval with stringent statistical validation would enable increased performance of such approaches to reduce manual labor cost with higher precision and efficiency. Some of these advanced tools/databases are available online (Percha and Altman, 2018, Yu et al., 2019).

Another parameter to consider is the fact that clinical utility of a genome-guided intervention cannot only be judged solely based on statistical evaluation but also on the effectiveness of population-wise patient treatment individualization. Both of these elements are needed to assess the value of healthcare resources used. Third, in this study, we focused on mining the biomedical literature for supporting precision medicine, whereas other studies have shown value in using additional text sources such as electronic health records and clinical trial data, which are of higher value addition. Systematically integrating data and results from multiple textual sources might be worth exploring in future research for robustness of such a text mining pipeline.

In conclusion, we have shown our approach for text mining drug response association with genes/genetic variants from the global biomedical literature published in PGx. This approach can be exploited to generate PGx relationships published for medications administered among different types of diseases. Our approach can thus apply broadly to a variety of diseases and their respective drugs administered. On comparative analysis with currently used FDA-approved PGx drug label biomarkers commercially available, a 68% overlap of our approach confirms the accuracy of our approach and demonstrates that these text-mined results are potentially useful for clinical value addition and widening the spectrum of clinical curation and improving therapeutic services.

## Data Availability

The raw data supporting the conclusions of this manuscript will be made available by the authors, without undue reservation, to any qualified researcher.

## Author Contributions

DG and RK originally conceived and designed the experiments of this article. DG and JR performed the experiments. DG conducted the statistical analysis and wrote the first draft. DG, JR, PT, PS, SB, SG, and RK contributed to the study protocol and analysis tools. DG, JR, PT, and RK analyzed the data. DG, PS, SB, and RK wrote the paper. DG, JR, and RK managed the data. PT, SG, CA, KS, LS, YH, MK, SR, and RK scrutinized the data and manuscript. All authors interpreted the data, reviewed successive drafts, and approved the final version of the article. The study was overall supervised by RK.

## Funding

Financial support for this project has been provided by the Council of Scientific and Industrial Research (CSIR) funded project (MLP1804).

## Conflict of Interest Statement

The authors declare that the research was conducted in the absence of any commercial or financial relationships that could be construed as a potential conflict of interest.
