# Corrigendum: Global Text Mining and Development of Pharmacogenomic Knowledge Resource for Precision Medicine

**DOI:** 10.3389/fphar.2020.614445

**Published:** 2020-12-04

**Authors:** Debleena Guin, Jyoti Rani, Priyanka Singh, Sandeep Grover, Shivangi Bora, Puneet Talwar, Muthusamy Karthikeyan, K. Satyamoorthy, C. Adithan, S. Ramachandran, Luciano Saso, Yasha Hasija, Ritushree Kukreti

**Affiliations:** ^1^ Genomics and Molecular Medicine Unit, Council of Scientific and Industrial Research (CSIR)—Institute of Genomics and Integrative Biology (IGIB), New Delhi, India; ^2^ Department of Biotechnology, Delhi Technological University, Delhi, India; ^3^ Department of Biomedical Sciences, Acharya Narayan Dev College, University of Delhi, New Delhi, India; ^4^ G N Ramachandran Knowledge Centre, Council of Scientific and Industrial Research (CSIR)—Institute of Genomics and Integrative Biology (IGIB), New Delhi, India; ^5^ Academy of Scientific & Innovative Research (AcSIR), New Delhi, India; ^6^ Institute of Medical Biometry and Statistics, University of Lübeck University Medical Center Schleswig-Holstein – Campus Lübeck, Lübeck, Germany; ^7^ Institute of Human Behaviour and Allied Sciences, Delhi, India; ^8^ Department of Bioinformatics, Alagappa University, Karaikudi, India; ^9^ School of Life Sciences, Manipal University, Manipal, India; ^10^ Central Inter-Disciplinary Research Facility (CIDRF), Pondicherry, India; ^11^ Department of Physiology and Pharmacology “Vittorio Erspamer,” Sapienza University of Rome, Rome, Italy

**Keywords:** text mining, precision medicine, disease-drug-gene-mutation relationship, pharmacogenomic markers, pharmacogenomic knowledgebase

In the original article, Supplementary Table 11 is missing, as published. Supplementary Table 11 is mentioned in the article text in *Discussion* section, para 4, but it is missing from the Supplementary file. Also, **Supplementary Table 12** mentioned under section **‘A Resource for Pharmacogenomic Evaluation’** in the original article should be **Supplementary Table 11**. 

The authors apologize for this error and state that this does not change the scientific conclusions of the article in any way. The original article has been updated.

