# Supplementary material for: Corrigendum: Global Text Mining and Development of Pharmacogenomic Knowledge Resource for Precision Medicine
Source: Front Pharmacol. 2020 Dec 4;11:614445. doi: 10.3389/fphar.2020.614445 (PMC7746840; doi:10.3389/fphar.2020.614445)
Supplement: Supplementary file 1 [file datasheet1.docx]

**Global text mining and development of pharmacogenomic knowledge resource for precision medicine**

**Debleena Guin^1, 2^, Jyoti Rani^3,4^, Priyanka Singh^1, 5^, Sandeep Grover^6^, Shivangi Bora^1, 2^, Puneet Talwar^7^, M. Karthikeyan^8^, K. Satyamoorthy^9^, C. Adithan^10^, S. Ramachandran^4,5^, Luciano Saso^11^, Yasha Hasija^2^ and Ritushree Kukreti^1, 5^***

**^1^**Genomics and Molecular Medicine Unit, Council of Scientific and Industrial Research (CSIR) - Institute of Genomics and Integrative Biology (IGIB), New Delhi-110007, India

^2^ Department of Biotechnology, Delhi Technological University, Shahbad Daulatpur, Delhi, India

^3^ Department of Biomedical Sciences, Acharya Narayan Dev College, University of Delhi, New Delhi, India

^4^ G N Ramachandran Knowledge Centre, Council of Scientific and Industrial Research (CSIR) - Institute of Genomics and Integrative Biology (IGIB), New Delhi-110007, India

^5^ Academy of Scientific & Innovative Research (AcSIR), CSIR- Institute of Genomics and Integrative Biology (CSIR-IGIB) Campus, New Delhi, India.

^6^Institute of Medical Biometry and Statistics, University of Lübeck University Medical Center Schleswig-Holstein - Campus Lübeck, Lübeck, Germany

^7^Institute of Human Behaviour and Allied Sciences, Dilshad Garden, Delhi, India

^8^Department of Bioinformatics, Alagappa University, Karaikudi, India

^9^School of Life Sciences. Manipal University, Manipal, India

^10^Central Inter-Disciplinary Research Facility (CIDRF), Sri Balaji Vidyapeeth MGMCRI Campus, Pondy-Cuddalore Main Road, Pondicherry, India

^11^Department of Physiology and Pharmacology "Vittorio Erspamer", Sapienza University of Rome, P. le Aldo Moro 5, 00185 Rome, Italy.

***Correspondence:**

Ritushree Kukreti

ritus@igib.res.in; ritushreekukreti@gmail.com

## Key words: text mining_1_, precision medicine_2_, disease-drug-gene-mutation relationship_3_, Pharmacogenomic markers_4_, pharmacogenomic knowledgebase_5_

**Supplementary Information**

**Contents**

Detailed Methods

1. **Supplementary Figure 1 – Detailed pipeline**
2. **Supplementary Table 1- Consolidated**
3. **Supplementary Table 2- Diseases**
4. **Supplementary Table 3- Drugs**
5. **Supplementary Table 4- Genes**

Results

1. **Supplementary Table 5- Statistical evaluation & formulae used**
2. **Supplementary Table 6 – Performance per entity**
3. **Supplementary Table 7 - Performance per relation type**
4. **Supplementary Table 8 - Sources of errors for entity normalization**
5. **Supplementary Table 9 - Performance per entity type**
6. **Supplementary Table 10 -Performance per entity type, as estimated by manual evaluation.**

Discussion:

- 1. **Supplementary table – 11 – A comprehensive table of 2,304 human PGx relationships between genetic variant- gene- drug-disease with their corresponding evidences available.**
  2. **Additional table for the readers (not cited in the article): PGx markers common between FDA approved drug labels available and proposed pipeline**

PubMed

Pre-compiled PGx MeSH enhanced PubMed Query

Pharmacogenomics Query (Best match, Human studies, English as on 30.11.2017) (633074 PMIDs)

Review articles (114545 PMIDs)

Remaining Articles (518529 PMIDs)

Non-Human studies (88209 PMIDs)

Remaining Articles (430320 PMIDs)

Biomedical entity recognition using PubTator

Articles with empty fields in Disease/ Drug/Gene/Polymorphism (183637 PMIDs)

Remaining Articles (246695 PMIDs)

Articles with non-genetic studies, or cytogenetic studies or disease association related to confounding factors (66607 PMIDs)

Remaining Articles (180088 PMIDs)

Normalization of biological entity

| **Entities** | **Normalized with** |
| --- | --- |
| Diseases | Parent disease of WHO ICD v.10 classes |
| Chemical/Drug | DrugBank ID |
| Gene | HGNC Gene ID |

Articles not normalized (140303 PMIDs)

Remaining Articles (184560 PMIDs)

| Disease (1753) |
| --- |
| Chemical/Drug (666) |
| Gene (4132) |
| Polymorphism (33942) |
| Population (179) |

Disease-wise segregation

| **Disease- wise segregation** |
| --- |
| Neoplasm (34444 PMIDs) |
| Neurological Diseases (13893 PMIDs) |
| Mental & Behavioural (10892 PMIDs) |
| Metabolic (7628 PMIDs) |
| Cardiovascular (10385 PMIDs) |
| Blood & related disorder (5634 PMIDs) |
| Others (18808 PMIDs) |

Pharmacogenomic relationship extraction

Performance comparison of the proposed pipeline with OMIM, CTD & PharmGKB

Frequency ranking of extracted 2304 relationships

**<Disease-Drug-Gene-Polymorphism- Population>**

**Supplementary Figure 1:** General flowchart summarizing the Global PGx Text Mining data extraction process.

| **Biological Entities** | **Diseases** | **Drugs** | **Genes** | **Populations** | **Polymorphism** |
| --- | --- | --- | --- | --- | --- |
| No. | 74459 | 62781 | 73221 | 1079 | 133942 |

**Supplementary Table 1:** Consolidated information about all the biological entities and number of each of them extracted in the pipeline.

| **Biological Entities** | **Infectious diseases** | **Neoplasms** | **Blood and immune disorders** | **Metabolic diseases** | **Mental disorders** | **CNS Diseases** | **Circulatory disease** | **Respiratory disease** | | | **Digestive disease** | **Skin Disease** | **Muscle** | **Genitourinary system** | **Congenital Disease** |
| --- | --- | --- | --- | --- | --- | --- | --- | --- | --- | --- | --- | --- | --- | --- | --- |
| No. | 50 | 319 | 54 | 75 | 77 | 82 | 111 | | 30 | 30 | | 19 | 37 | 19 | 99 |
| Frequency | 2091 | 34444 | 5634 | 7628 | 10892 | 13893 | 10385 | | 3734 | 1953 | | 1184 | 3846 | 1909 | 2948 |

**Supplementary Table 2:** Detailed classification of all the disease extracted in the pipeline and number of genes extracted under each category with their frequency of occurrence.

| **Drugs** | **Anti- coagulant** | **Immunosuppressant** | **Analgesics** | **Respiratory drugs** | **CNS Drug** | **Drugs acting on Kidney** | **Gastro-intestinal** | **Anti-viral** | **Drugs CNS** | **Anti-psychotic** | **Cardiovascular drugs** | **Anti-Cancer** | **Anti-microbial** | **Hormone- related drugs** | **Others** |
| --- | --- | --- | --- | --- | --- | --- | --- | --- | --- | --- | --- | --- | --- | --- | --- |
| No. | 56 | 28 | 82 | 63 | 56 | 23 | 30 | 38 | 106 | 69 | 45 | 82 | 91 | 189 | 1593 |
| Frequency | 426 | 982 | 1809 | 1925 | 1426 | 1058 | 1263 | 1056 | 3238 | 3284 | 1562 | 2059 | 1923 | 1099 | 4877 |

**Supplementary** **Table 3:** Detailed classification of all the drugs extracted in the pipeline and number of genes extracted under each category with their frequency of occurrence.

| **Genes** | **Phase I genes** | **Phase II genes** | **Transporters** | **Receptor genes** | **MHC genes** | **Zinc finger genes** | **Growth Factor genes** | **Others** |
| --- | --- | --- | --- | --- | --- | --- | --- | --- |
| No. | 90 | 43 | 537 | 900 | 20 | 236 | 68 | 8201 |
| Frequency | 22244 | 18124 | 15699 | 56796 | 6539 | 2359 | 5397 | 283747 |

**Supplementary Table 4:** Detailed classification of all the genes extracted in the pipeline and number of genes extracted under each category with their frequency of occurrence.

| **Parameters** | **Formula** |
| --- | --- |
| Sensitivity | TP %  TP+TN |
| Specificity | TN %  TN+FP |
| Efficiency | TP+TN %  TP+TN+FP+FN |
| Precision | TP  TP+FP |
| Recall | TP  TP+FN |
| Accuracy | TP+TN %  TP+TN+FP+FN |
| F-measure | 2X Precision x Recall  Precision + Recall |

**Gold standard**

|  | **+ve** | **-ve** |
| --- | --- | --- |
| **+ve** | TP | FP |
| **-ve** | FN | TN |

**Test**

**Data**  2 x 2 contingency table

**Supplementary Table 5:** Details of formulae used for performance evaluation of the proposed pipeline.

| **Sl. No.** | **Entity type** | **Total** | **Unique** | **PharmGKB** | **OMIM** | **CTD** |
| --- | --- | --- | --- | --- | --- | --- |
| 1 | Disease | 74459 | 9539 | 3540 | 202 | 12981 |
| 2 | Drug | 62781 | 1693 | 3794 | - | 169460 |
| 3 | Gene | 73221 | 15794 | 27042 | 16590 | 472994 |
| 4 | SNP | 133942 | 20339 | 6443 | 25713 | - |
| 5 | Population | 1079 | 208 | 179 | - | - |

**Supplementary Table 6:** **Number of entities found in 180078 abstracts.** Performance per entity type compared with other available datasets, as estimated by manual evaluation. This data has been used for error calculation. (Refer Table 1)


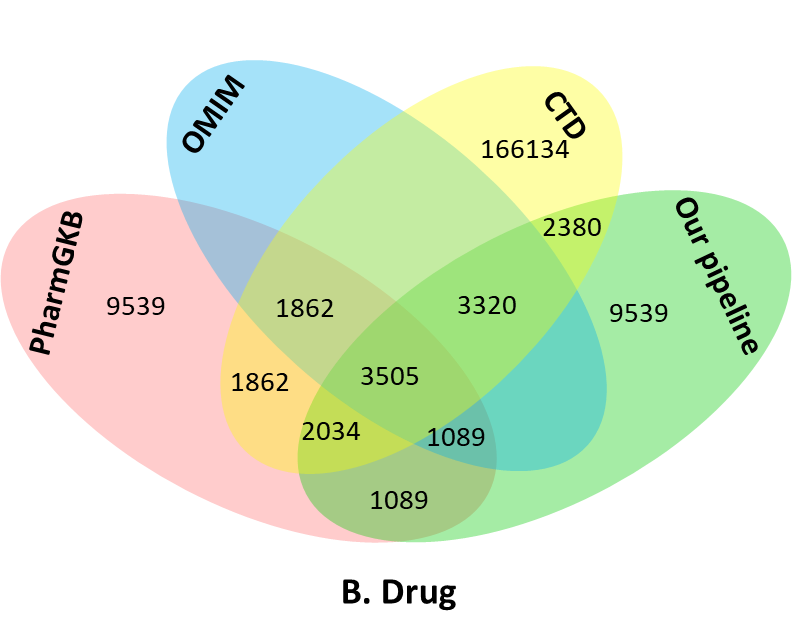

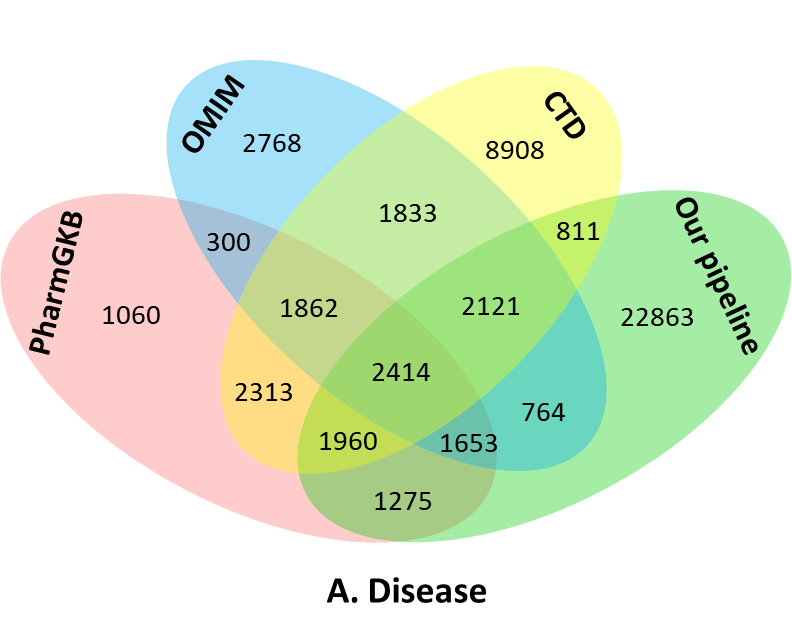


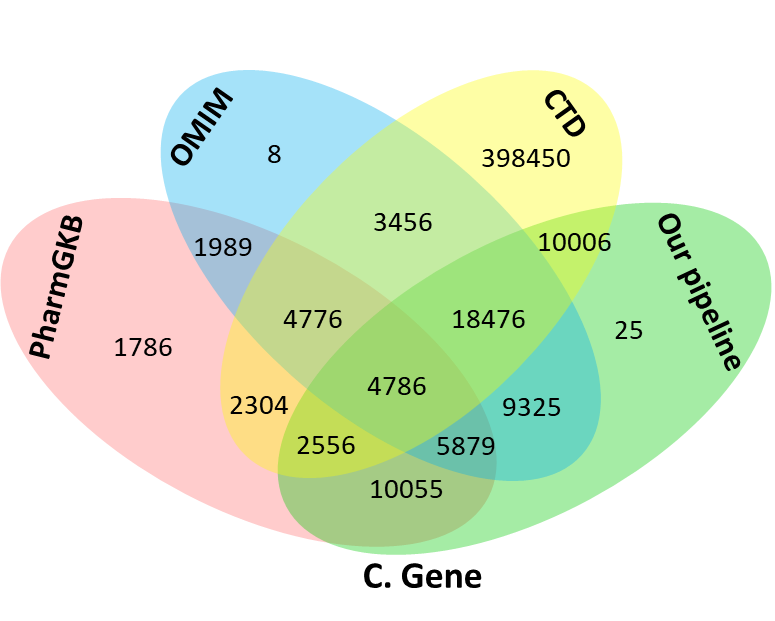


**Supplementary Figure 2:** Comparison between existing databases for pharmcogenomics relationships and that from our pipeline. The number of (A. Diseases, B. Drugs, C. Genes) related to pharmacogenomics from the three databases and our pipeline and common entities between them.

| **Sl. No.** | **Entity type** | **Total** | **Unique** | **PharmGKB** | **OMIM** | **CTD** |
| --- | --- | --- | --- | --- | --- | --- |
| 1 | Disease- Drug | 117834 | 26268 | 4343 | - | 113014 |
| 2 | Disease-Gene | 709987 | 102881 | 8147 | 2864 | 12546 |
| 3 | Drug-Gene | 191054 | 31593 | 6820 | - | 94788 |
| 4 | Drug-SNP | 10809 | 5491 | 6778 | - | - |
| 5 | Gene-SNP | 101477 | 21704 | 645 | 684 | - |
| 6 | Population- SNP | 12897 | 6765 | - | - | - |

**Supplementary Table 7:** **Number of PGx relationships as extracted by our proposed approach in 180078 abstracts**. Performance per relation type compared with other available datasets, as estimated by manual evaluation.

| - - 1. **Disease Normalization** | |
| --- | --- |
| **Causes** | **e.g. of evidence sentences** |
| **False negatives** | **Evidence from abstract** |
| Variations of disease based on different structural, morphological or pathological types | Parkinson’s disease plus syndromes are considered within Parkinson’s disease category |
| Segmentation of name failed | Nephropathy or kidney disorder |
| Syntactically unrelated | Gonococcal infection or gonorrhoea, osteomata or osteoma |
|  |  |
| **False positives** | **Evidence from abstract** |
| Unrecognized variations (symptoms, and other ambiguity not classified as disease) | Pain, and other symptoms not included as a disease |
| Context filtering | Hepatic disease or liver disease |
| Overlap of names not recognized | Oral tardive dyskinesia, Tardive dyskinesia, dyskinesia |
| Multiple identifiers for one name | Mesial temporal lobe epilepsy, temporal lobe epilepsy, temporal lobe seizure |
|  |  |
| - - 1. **Drug Normalization** | |
| **Causes** | **e.g. of evidence sentences** |
| **False negatives** | **Evidence from abstract** |
| Variations of drug names, metabolite or other unrecognized chemical names | N-acetyl isoniazid,  isonicotinylhydrazide, Isovit |
| Segmentation of name failed | Methotrexate Sodium or amethopterin |
| Syntactically unrelated | Valproic acid, sodium valproate |
|  |  |
| **False positives** | **Evidence from abstract** |
| Unrecognized variations (symptoms, and other ambiguity not classified as disease) | Levodopa, L-dopa, Sinemet |
| Triggered by wrong name boundary | Glucose, hormones, etc. |
| Context filtering | Vitamin A, retinoic acid |
| Overlap of names not recognized | retinoic acid or retinol |
| Multiple identifiers for one name | 25-Dihydroxyvitamin D3  or Vitamin D3 or Vitamin D |
|  |  |
| - - 1. **Gene Normalization** | |
| **Causes** | **e.g. of evidence sentences** |
| **False negatives** | **Evidence from abstract** |
| Unmatched, synonyms, withdrawn genes | ABHD11-AS2 |
| Pseudogenes, miRNA , orf and other non-protein coding genes | MIR4587 |
| Segmentation of name failed | hOBP (IIb)/hOBPIIb |
| Syntactically unrelated | polycomblike/PHD finger protein |
|  |  |
| **False positives** | **Evidence from abstract** |
| Unrecognized variations | DCoHm/DCOHM |
| Triggered by wrong name boundary | type II IL-1 receptor |
| Context filtering | CD4+, CD8+ |
| Overlap of names not recognized | T Brachyury |
| Multiple identifiers for one name | Multidrug resistance protein (MDR) |
|  |  |

**Supplementary Table 8:** **Sources of errors for entity normalization.** Analysis of errors that occurred during entity recognition using *pubtator*. All the error occurred due to false negatives and false positives, and examples of errors.

| **Sl. No.** | **Entity type** | **TP** | **FP** | **FN** | **Total** | **Precision** | **Recall** | **F-measure** |
| --- | --- | --- | --- | --- | --- | --- | --- | --- |
| 1 | Disease | 283 | 32 | 13 | 328 | 89.8 | 95.6 | 92.6 |
| 2 | Drug | 117 | 10 | 33 | 160 | 87.1 | 78.0 | 84.5 |
| 3 | Gene | 509 | 54 | 40 | 603 | 90.4 | 92.7 | 91.5 |
| 4 | SNP | 53 | 0 | 19 | 72 | 94.6 | 73.6 | 84.8 |
| 5 | Population | 30 | 0 | 7 | 37 | 98.0 | 81.1 | 89.6 |
|  | **Total** | 1134 | 101 | 125 | 1360 | 96.0 | 88.7 | 91.9 |

**Supplementary Table 9:** **Performance per entity type, as estimated by manual evaluation.** TP, true positive; FP, false positive; FN, false negative; precision, recall, f-score in %.

| **Sl. No.** | **Relation type** | **TP** | **FP** | **FN** | **Total** | **Precision** | **Recall** | **F-measure** |
| --- | --- | --- | --- | --- | --- | --- | --- | --- |
| 1 | Disease- Drug | 42 | 8 | 15 | 65 | 84.0 | 73.7 | 78.5 |
| 2 | Disease-Gene | 356 | 113 | 22 | 499 | 75.9 | 94.2 | 84.1 |
| 3 | Drug-Gene | 136 | 39 | 18 | 199 | 77.7 | 88.3 | 82.7 |
| 4 | Drug-SNP | 111 | 45 | 12 | 170 | 71.2 | 90.2 | 79.6 |
| 5 | Gene-SNP | 47 | 33 | 17 | 98 | 58.8 | 73.4 | 65.3 |
| 6 | Population- SNP | 13 | 9 | 2 | 25 | 59.1 | 86.7 | 70.3 |

**Supplementary Table 10:** **Performance per entity-entity relationship type, as estimated by manual evaluation.** TP, true positive; FP, false positive; FN, false negative; precision, recall, f-score in %.

| **Genetic variant** | **Gene** | **Drug** | **Disease** | **Evidences (PMIDs)** |
| --- | --- | --- | --- | --- |
| NAT2*5A | NAT2 | 1,7-dimethylxanthine | Psychiatric disorders | 17011540 |
| rs36029 | SLC6A2 | 3,4-methylenedioxymethamphetamine | Cancer | 29198060 |
| rs3093726 | LTA | abacavir | Viral infection | 16998491 |
| HLA-B*57:01 | HLA-B | abacavir | Viral infection | 28399804 |
| HLA-DQB1*03:03:03 | HLA-DQB1 | abacavir | Viral infection | 11888582 |
| HLA-B*57:01 | HLA-B | abacavir | Viral infection | 24625462 |
| Pl A1/A2 | ITGB3 | abciximab | heart attack, stroke | 20938371 |
| rs5918 | ITGB3 | abciximab | heart attack, stroke | 10704169 |
| rs5918 | ITGB3 | abciximab | heart attack, stroke | 20938371 |
| rs3828743 | TSPYL1 | abiraterone | Cancer | 29027195 |
| rs2486758 | CYP17A1 | abiraterone | Cancer | 25157341;27409606 |
| rs2058878 | GLRB | acamprosate | Alcoholism | 25290263 |
| rs145489027 | KCNIP4 | Ace Inhibitors, Plain | Hypertension | 26169577;28084903 |
| rs8012552, rs8016905 | BDKRB2 | Ace Inhibitors, Plain | Hypertension | 10904024;11699055;12522467;19744011;21052031;21832968 |
| rs7661530 | KCNIP4 | Ace Inhibitors, Plain | Hypertension | 26169577;28084903 |
| rs1495509 | KCNIP4 | Ace Inhibitors, Plain | Hypertension | 26169577;28084903 |
| rs7675300 | KCNIP4 | Ace Inhibitors, Plain | Hypertension | 26169577;28084903 |
| rs16870989 | KCNIP4 | Ace Inhibitors, Plain | Hypertension | 26169577;28084903 |
| rs1799722 | BDKRB2 | Ace Inhibitors, Plain | Hypertension | 10904024;11699055;12522467;19744011 |
| rs62151109 | CLASP1 | Ace Inhibitors, Plain | Hypertension | 28084903 |
| rs2016848 | MME | Ace Inhibitors, Plain | Hypertension | 21052031 |
| rs3815583, rs71647871 | CES1 | Ace Inhibitors, Plain | Hypertension | 26761119 |
| rs4459610 | ACE3P | Ace Inhibitors, Plain | Hypertension | 21832968 |
| rs6838116 | KCNIP4 | Ace Inhibitors, Plain | Hypertension | 26169577 |
| rs5182 | AGTR1 | Ace Inhibitors, Plain | Hypertension | 18347611 |
| rs55894764 | VKORC1 | acenocoumarol | Blood clots, thrombosis, stroke | 23691226 |
| K234N, N235T | SULT1A4 | acetaminophen | Pain symptom | 26049587;29705271 |
|  | SULT1E1 | acetaminophen | Pain symptom | 23462933;26049587 |
| UGT1A6*1a | UGT1A6 | acetaminophen | Pain symptom | 15761113;17164591;21666065 |
| HLA-A*33:03 | HLA-A | acetaminophen | Pain symptom | 21545408 |
| HLA-DQB1*02:02 | HLA-DQB1 | acetaminophen | Pain symptom | 21545408 |
| CYP2E1*1 | CYP2E1 | acetaminophen | Pain symptom | 8659683 |
| rs1902023 | UGT2B15 | acetaminophen | Pain symptom | 28663312 |
| HLA-B*59:01:01:01 | HLA-B | acetazolamide | Seizures, Epilepsy | 21342230 |
| rs5743890 | TOLLIP | acetylcysteine | Lung Diseases, emphysema, bronchitis, cystic fibrosis | 26331942 |
| EPHX1 poor metabolizer | EPHX1 | acetylcysteine | Lung Diseases, emphysema, bronchitis, cystic fibrosis | 25999707 |
| rs10919563 | PTPRC | adalimumab | Inflammation, arthritis | 20309874;23007924 |
| 308 G/G, 857 C/C | TNF | adalimumab | Inflammation, arthritis | 12190096;12759288;12847678;15834068;16720636;16909270;17343250;17673491;18050183;18438841;18713756;19365401;22129793;22760475;22960943;23057546;24192118;26244882 |
| rs7574865 | STAT4 | adalimumab | Inflammation, arthritis | 23007924;28107378 |
| rs3761847 | TRAF1 | adalimumab | Inflammation, arthritis | 23007924;25834819 |
| rs1801274 | FCGR2A | adalimumab | Inflammation, arthritis | 24048425;24667440;25823785;27044681 |
| rs3761847 | TRAF1 | adalimumab | Inflammation, arthritis | 23007924;25834819 |
| V158F, rs396991, rs1801274 | FCGR2A | adalimumab | Inflammation, arthritis | 24048425;24667440;25823785;27044681 |
| rs1800629 | LTA | adalimumab | Inflammation, arthritis | 12190096;12759288;12847678;15834068;16720636;16909270;17343250;17673491;18050183;18438841;18713756;19365401;22129793;22760475;22960943;23057546;24192118;26244882 |
| rs396991 | FCGR3A | adalimumab | Inflammation, arthritis | 24048425;25823782;27044681 |
| rs10919563 | PTPRC | adalimumab | Inflammation, arthritis | 20309874;23007924 |
| rs7574865 | STAT4 | adalimumab | Inflammation, arthritis | 23007924;28107378 |
| rs6920220 | AL356234.2 | adalimumab | Inflammation, arthritis | 23007924 |
| rs7574865 | STAT4 | adalimumab | Inflammation, arthritis | 25712183 |
| Codon 10C/T, Codon 25 G/C | TGFB1 | adalimumab | Inflammation, arthritis | 22129793 |
| rs3794271 | SLCO1C1 | adalimumab | Inflammation, arthritis | 23651021 |
| rs763780 | IL17F | adalimumab | Inflammation, arthritis | 26415694 |
| rs2071303 | HFE | adalimumab | Inflammation, arthritis | 27115882 |
| HLA-DRB1*04:01:05 | HLA-DRB1 | adalimumab | Inflammation, arthritis | 25919528 |
| rs1061622 | TNFRSF1B | adalimumab | Inflammation, arthritis | 25537528 |
| rs1050501 | FCGR2B | adalimumab | Inflammation, arthritis | 25823782 |
| rs1130864 | CRP | adalimumab | Inflammation, arthritis | 27096233 |
| rs7301582 | NKG2A | adalimumab | Inflammation, arthritis | 26453102 |
| rs6691117 | CR1 | adalimumab | Inflammation, arthritis | 23856853 |
| rs2228145 | IL6R | adalimumab | Inflammation, arthritis | 19926672 |
| rs1059510, HLA-E*01:03:01/01:03:01 | HLA-E | adalimumab | Inflammation, arthritis | 26307125 |
| rs3213094 | IL12B | adalimumab | Inflammation, arthritis | 27564082 |
| rs25648 | VEGFA | adalimumab | Inflammation, arthritis | 28639493 |
| rs1800795 | IL6 | adalimumab | Inflammation, arthritis | 24253594 |
| rs2233945 | PSORS1C2 | adalimumab | Inflammation, arthritis | 28107378 |
| rs1799724 | LTA | adalimumab | Inflammation, arthritis | 17673491 |
| rs767455 | TNFRSF1A | adalimumab | Inflammation, arthritis | 22480748 |
| rs11568626 | SLC22A6 | adefovir dipivoxil | Viral infection | 15914676 |
| rs34548976 | - | adrenergics, inhalants | Hypertension, cardiovascular diseases | 25918834 |
| rs17069895 | TNFRSF11A | alendronate | Bone disease, Paget's disease | 26426211 |
| rs2241529 | DKK1 | alendronate | Bone disease, Paget's disease | 26780085 |
| rs10161126 | - | alendronate | Bone disease, Paget's disease | 25223561 |
| rs1256049 | ESR2 | alendronate | Bone disease, Paget's disease | 12137804 |
| HLA-B*51:01:01 | HLA-B | allopurinol | High uric acid levels | 19696695;21912425;23669020 |
| HLA-B*07:02:01 | HLA-B | allopurinol | High uric acid levels | 21912425;23669020 |
| HLA-B*48:01 | HLA-B | allopurinol | High uric acid levels | 19002350;21912425;23669020;24858023 |
| HLA-B*40:01:01 | HLA-B | allopurinol | High uric acid levels | 19696695;21912425 |
| HLA-B*39:01:01:01 | HLA-B | allopurinol | High uric acid levels | 21912425;23669020 |
| HLA-B*54:01:01 | HLA-B | allopurinol | High uric acid levels | 21912425;23669020 |
| rs3815087 | PSORS1C1 | allopurinol | High uric acid levels | 21801394;29193002 |
| HLA-B*46:01:01 | HLA-B | allopurinol | High uric acid levels | 19696695;21912425;23669020 |
| HLA-C*08:01:01 | HLA-C | allopurinol | High uric acid levels | 21912425;24858023 |
| rs3099844 | HCP5 | allopurinol | High uric acid levels | 29193002;29392141 |
| HLA-B*52:01:01:01 | HLA-B | allopurinol | High uric acid levels | 21912425;23669020 |
| HLA-A*11:01:01 | HLA-A | allopurinol | High uric acid levels | 21912425;23669020 |
| HLA-B*56:01:01 | HLA-B | allopurinol | High uric acid levels | 21912425;23669020 |
| HLA-B*5801 | HLA-B | allopurinol | High uric acid levels | 21801394;29193002;29392141 |
| HLA-A*26:01:01 | HLA-A | allopurinol | High uric acid levels | 21912425;23669020 |
| HLA-A*31:01:02 | HLA-A | allopurinol | High uric acid levels | 21912425;23669020 |
| HLA-A*24:02:01:01 | HLA-A | allopurinol | High uric acid levels | 21912425;23669020 |
| HLA-B*48:01:01 | HLA-B | allopurinol | High uric acid levels | 19002350;24858023 |
| HLA-B*35:01:01:01 | HLA-B | allopurinol | High uric acid levels | 21912425;23669020 |
| HLA-A*33:01:01 | HLA-A | allopurinol | High uric acid levels | 17587850;23669020 |
| rs3099844 | HCP5 | allopurinol | High uric acid levels | 29193002 |
| HLA-B*37:01:01 | HLA-B | allopurinol | High uric acid levels | 23669020 |
| HLA-DRB1*14:01:01 | HLA-DRB1 | allopurinol | High uric acid levels | 24858023 |
| HLA-C*03:03:01 | HLA-C | allopurinol | High uric acid levels | 21912425 |
| rs3130501 | POU5F1 | allopurinol | High uric acid levels | 21801394 |
| rs1594 | CFLAR | allopurinol | High uric acid levels | 15743917 |
| rs9263726 | PSORS1C2 | allopurinol | High uric acid levels | 29392141 |
| HLA-B*58:01:01 | HLA-B | allopurinol | High uric acid levels | 28509689 |
| HLA-B*18:02 | HLA-B | allopurinol | High uric acid levels | 19696695 |
| HLA-C*14:03 | HLA-C | allopurinol | High uric acid levels | 21912425 |
| rs10011796 | ABCG2 | allopurinol | High uric acid levels | 26810134 |
| HLA-B*5801 | HLA-B | allopurinol | High uric acid levels | 21801394 |
| m.1494C>T | MT-RNR1 | amikacin | Bacterial meningitis | 10633132;11079536;11174059;11313749;11857751;12031626;12920080;14681830;14699607;14755216;15555598;15708009;15841390;15917167;16168391;16375862;16380089;16458854;16631122;16875663;16955413;17085680;17341440;17434445;17637808;17698030;17698299;17999439;18790089;18820594;18830133;19376484;19682603;19818876;20100600;20111055;20353758;20416460;21162657;21495045;21504270;21725156;22475488;24703164;25155176;25515069;25744662;27397648;27427311;7689389;8285309;8414970;9039999;9111378;9164619;9391883;9490575;9779807;9831149;9950117 |
| rs267606619 | MT-ND1 | amikacin | Bacterial meningitis | 14681830;16380089;17085680;17434445;17698030;17698299;18830133;19682603;20100600;20416460;25515069;27397648 |
| rs267606617 | MT-ND1 | Amikacin | Bacterial meningitis | 10633132;11174059;11857751;12031626;12920080;14699607;14755216;15708009;15841390;15917167;16168391;16375862;16458854;16631122;16955413;17341440;17637808;17999439;18790089;18820594;18830133;19376484;19818876;20100600;20111055;20353758;20416460;21162657;21495045;21504270;21725156;22475488;24703164;25155176;25515069;25744662;27427311;7689389;8285309;8414970;9039999;9111378;9164619;9391883;9490575;9779807;9831149;9950117 |
| rs267606618 | MT-ND1 | Amikacin | Bacterial meningitis | 11079536;11313749;15555598;15841390;16875663;25515069 |
| rs12720441 | KCNH2 | amiodarone | Cardiovascular disease | 11997281 |
| rs7566605 | - | amisulpride | Psychiatric Disorders | 20504252 |
| rs1079597 | DRD2 | amisulpride | Psychiatric Disorders | 25679126 |
| rs17782313 | - | amisulpride | Psychiatric Disorders | 23920449 |
| rs10042486 | HTR1A | amisulpride | Psychiatric Disorders | 22120873 |
| rs4148739 | ABCB1 | amitriptyline | Psychiatric Disorders | 18215618;24192121 |
| rs10280101 | ABCB1 | amitriptyline | Psychiatric Disorders | 18215618 |
| rs877087 | RYR3 | amlodipine | Cardiovascular disease | 22664477 |
| rs4291 | ACE | amlodipine | Cardiovascular disease | 20577119 |
| rs200148 | LINC01277 | amlodipine | Cardiovascular disease | 25171760 |
| rs5065 | CLCN6 | amlodipine | Cardiovascular disease | 18212314 |
| rs11122576 | AGT | amlodipine | Cardiovascular disease | 25278896 |
| rs3784943 | CDH13 | amphetamine | Psychiatric disorder, major depression | 27771748 |
| rs12364283 | DRD2 | amphetamine | Psychiatric disorder, major depression | 19968402 |
| rs2281617 | IPCEF1 | amphetamine | Psychiatric disorder, major depression | 21029375 |
| rs2070995 | KCNJ6-AS1 | Analgesics | Pain symptom | 19756153;20220551 |
| rs2070995 | KCNJ6 | Analgesics | Pain symptom | 19756153;20220551 |
| rs1611115 | DBH | Analgesics | Pain symptom | 25096645 |
| rs3732219 | UGT1A6 | anastrozole | Cancer | 23371966 |
| rs1256061 | ESR2 | anastrozole | Cancer | 23894347 |
| rs73511817 | - | anastrozole | Cancer | 25148458 |
| rs12956925 | TNFRSF11A | anastrozole | Cancer | 26218592 |
| rs13260300 | - | anastrozole | Cancer | 27758888 |
| rs749292 | CYP19A1 | anastrozole | Cancer | 27747906 |
| rs6493497 | GLDN | anastrozole | Cancer | 20048079 |
| rs7319981 | SLC10A2 | anthracyclines and related substances | Cancer | 21900104;23441093 |
| rs729147 | ADH7 | anthracyclines and related substances | Cancer | 21900104;23441093 |
| rs4148350 | ABCC1 | anthracyclines and related substances | Cancer | 21900104;23441093 |
| rs17645700 | - | anthracyclines and related substances | Cancer | 21900104;23441093 |
| rs1736557 | FMO3 | anthracyclines and related substances | Cancer | 21900104;23441093 |
| rs2108623 | AC005336.1 | anthracyclines and related substances | Cancer | 21900104;23441093 |
| rs885004 | SLC28A3 | anthracyclines and related substances | Cancer | 21900104;23441093 |
| rs2305364 | SLC28A1 | anthracyclines and related substances | Cancer | 21900104;23441093 |
| rs10426377 | SULT2B1 | anthracyclines and related substances | Cancer | 21900104;23441093 |
| rs17863783 | UGT1A6 | anthracyclines and related substances | Cancer | 21900104;23441093 |
| rs1056892 | CBR3 | anthracyclines and related substances | Cancer | 18457324;22124095 |
| rs4877847 | SLC28A3 | anthracyclines and related substances | Cancer | 21900104;23441093 |
| rs2290271 | SLC28A1 | anthracyclines and related substances | Cancer | 21900104;23441093 |
| rs17583889 | HNMT | anthracyclines and related substances | Cancer | 21900104;23441093 |
| rs2019604 | SPG7 | anthracyclines and related substances | Cancer | 21900104;23441093 |
| rs9514091 | SLC10A2 | anthracyclines and related substances | Cancer | 21900104;23441093 |
| rs7853758 | SLC28A3 | anthracyclines and related substances | Cancer | 21900104;23441093 |
| rs4261716 | UGT1A10 | anthracyclines and related substances | Cancer | 21900104;23441093 |
| rs1149222 | ABCB4 | anthracyclines and related substances | Cancer | 21900104;23441093 |
| rs11155012 | ECT2L | anthracyclines and related substances | Cancer | 25823661 |
| rs2232228 | HAS3 | anthracyclines and related substances | Cancer | 24470002 |
| rs28714259 | - | anthracyclines and related substances | Cancer | 27993963 |
| rs12468485 | GPR35 | anthracyclines and related substances | Cancer | 28961156 |
| rs700519 | CYP19A1 | Antiandrogens | Androgen dependent conditions, prostate cancer, benign prostatic hyperplasia | 23359804 |
| rs10033464 | LINC01438 | antiarrhythmics, class i and iii | Cardiovascular disease | 22726630 |
| rs622342 | SLC22A1 | Anticholinergics | Movement disorder, chronic obstructive pulmonary disorder | 20680652 |
| rs1439050 | NTRK2 | antidepressants | Psychiatric Disorders, Major Depression | 27378793;28244805 |
| rs17288723 | HTR2A | antidepressants | Psychiatric Disorders, Major Depression | 19924111 |
| rs365836 | CUX1 | antidepressants | Psychiatric Disorders, Major Depression | 22584459 |
| rs3824519 | NTRK2 | antidepressants | Psychiatric Disorders, Major Depression | 28244805 |
| rs2072446 | NGFR | antidepressants | Psychiatric Disorders, Major Depression | 27378793 |
| rs28364032 | CRHR1 | antidepressants | Psychiatric Disorders, Major Depression | 24422887 |
| rs1516338 | CHL1 | antidepressants | Psychiatric Disorders, Major Depression | 25943212 |
| rs35167514 | SLC22A1 | Antiemetics And Antinauseants | Nausea | 20921968 |
| rs3789243 | ABCB1 | antiepileptics | Seizures, Epilepsy | 17125969;19450124 |
| rs717620, rs3740066 | ABCC2 | antiepileptics | Seizures, Epilepsy | 22256867;22630058 |
| rs17183814 | SCN2A | antiepileptics | Seizures, Epilepsy | 19694741;21747585;23859570;25155934 |
| rs2304016 | SCN2A | antiepileptics | Seizures, Epilepsy | 18784617;25155934 |
| rs2804398 | ABCC2 | antiepileptics | Seizures, Epilepsy | 21449672 |
| rs487750 | KCNT1 | antiepileptics | Seizures, Epilepsy | 24279416 |
| rs717620 | ABCC2 | antiepileptics | Seizures, Epilepsy | 22256867 |
| rs1047891 | CPS1 | antiepileptics | Seizures, Epilepsy | 20456087 |
| rs2606345 | CYP1A1 | antiepileptics | Seizures, Epilepsy | 26951882 |
| rs1186745 | ABCB1 | antiepileptics | Seizures, Epilepsy | 17125969 |
| rs717620 | ABCC2 | antiepileptics | Seizures, Epilepsy | 22256867 |
| rs12817819 | ATP2B1 | Antihypertensives | Hypertension | 25385345 |
| rs3918242 | MMP9 | Antihypertensives | Hypertension | 21769110 |
| rs2965667 | - | Antiinflammatory agents, non-steroids | Inflammation | 25781442;29425227 |
| rs10306135 | PTGS1 | Antiinflammatory agents, non-steroids | Inflammation | 20538124;26067486 |
| rs7945189 | MMP1 | Antiinflammatory agents, non-steroids | Inflammation | 20538124 |
| rs1800896 | IL10 | Antiinflammatory agents, non-steroids | Inflammation | 28750137 |
| rs16973225 | AC104041.1 | Antiinflammatory agents, non-steroids | Inflammation | 25781442 |
| rs5789 | PTGS1 | Antiinflammatory agents, non-steroids | Inflammation | 26067486 |
| HLA-DRB1*11:01:01 | HLA-DRB1 | Antiinflammatory agents, non-steroids | Inflammation | 10200020 |
| rs1800566 | NQO1 | antiinflammatory and antirheumatic products, non-steroids | Inflammation | 17400324 |
| rs1760217 | DPYD | antineoplastic agents | Cancer | 21487324 |
| rs4910232 | LINC02752 | antineoplastic agents | Cancer | 26152742 |
| rs4606 | RGS2 | antipsychotics | Psychiatric Disorders | 17558307;18347610 |
| rs1468412 | GRM3 | antipsychotics | Psychiatric Disorders | 25096017;25209194 |
| rs6465084 | GRM3 | antipsychotics | Psychiatric Disorders | 25096017;25209194 |
| rs878949 | HSPG2 | antipsychotics | Psychiatric Disorders | 21808285 |
| rs3761656 | GCG | antipsychotics | Psychiatric Disorders | 24624910 |
| rs17793693 | PPARG | antipsychotics | Psychiatric Disorders | 24953421 |
| rs2513265 | - | antipsychotics | Psychiatric Disorders | 26788534 |
| rs10807344 | SLC25A27 | antipsychotics | Psychiatric Disorders | 21332312 |
| rs14240 | PDZD8 | antipsychotics | Psychiatric Disorders | 24018103 |
| rs1529461 | SLC1A3 | antipsychotics | Psychiatric Disorders | 25209194 |
| rs12467557 | NRXN1 | antipsychotics | Psychiatric Disorders | 24633560 |
| rs165815 | COMT | antipsychotics | Psychiatric Disorders | 27287786 |
| rs1933695 | AL035407.1 | antipsychotics | Psychiatric Disorders | 17558307 |
| rs10501087 | BDNF-AS | antipsychotics | Psychiatric Disorders | 23433505 |
| rs6977820 | DPP6 | antipsychotics | Psychiatric Disorders | 21826085 |
| rs4849127 | AC079753.1 | antipsychotics | Psychiatric Disorders | 25560300 |
| rs1059778 | IBA57 | antipsychotics | Psychiatric Disorders | 26323598 |
| rs8050136 | FTO | antipsychotics | Psychiatric Disorders | 24481458 |
| rs2445142 | HSPG2 | antipsychotics | Psychiatric Disorders | 21808285 |
| rs17300539 | ADIPOQ | antipsychotics | Psychiatric Disorders | 27741037 |
| rs1495741 | - | anti-TB | Tuberculosis | 23407048;27104815;29036176 |
| NAT2 slow acetylator | NAT2 | anti-TB | Tuberculosis | 24465778;24637014;28799976 |
| NAT2*5 | NAT2 | anti-TB | Tuberculosis | 10751073;11915035;12668988;16246623;16677176;16770646;17950035;18023090;18330759;18421452;18713495;19761367;20392357;21047300;21261721;21753138;21856096;22012226;22020825;22162992;22283902;22506592;22788240;22947533;23190413;23277397;23394127;23875638;24188272;24465778;24637014;24888881;26616266;27340556;29781872 |
| NAT2*14 | NAT2 | anti-TB | Tuberculosis | 10751073;11915035;12668988;16246623;16677176;16770646;17950035;18023090;18330759;18421452;18713495;19761367;20392357;21047300;21261721;21753138;21856096;22012226;22020825;22162992;22283902;22506592;22788240;22947533;23190413;23277397;23394127;23875638;24188272;24465778;24637014;24888881;26616266;27340556;29781872 |
| NAT2*6 | NAT2 | anti-TB | Tuberculosis | 10751073;11915035;12668988;16246623;16677176;16770646;17950035;18023090;18330759;18421452;18713495;19761367;20392357;21047300;21261721;21753138;21856096;22012226;22020825;22162992;22283902;22506592;22788240;22947533;23190413;23277397;23394127;23875638;24188272;24465778;24637014;24888881;26616266;27340556;29781872 |
| NAT2*7 | NAT2 | anti-TB | Tuberculosis | 10751073;11915035;12668988;16246623;16677176;16770646;17950035;18023090;18330759;18421452;18713495;19761367;20392357;21047300;21261721;21753138;21856096;22012226;22020825;22162992;22283902;22506592;22788240;22947533;23190413;23277397;23394127;23875638;24188272;24465778;24637014;24888881;26616266;27340556;29781872 |
| HLA-DQA1*01:02:01:01 | HLA-DQA1 | anti-TB | Tuberculosis | 12359646 |
| HLA-DQB1*05:01:01:01 | HLA-DQB1 | anti-TB | Tuberculosis | 25250564 |
| HLA-DQB1*02:01:01 | HLA-DQB1 | anti-TB | Tuberculosis | 12359646 |
| HLA-C*04:01:01:01 | HLA-C | anti-TB | Tuberculosis | 23153709 |
| rs1414334 | HTR2C | aripiprazole | Psychiatric Disorders | 20680028;27441116 |
| CYP2D6*6 | CYP2D6 | aripiprazole | Psychiatric Disorders | 17828532;17965519;21739267;24232129;26514968 |
| rs489693 | MC4R | aripiprazole | Psychiatric Disorders | 22566560;23799528;23920449;27217270 |
| CYP2D6*10 | CYP2D6 | aripiprazole | Psychiatric Disorders | 17828532;17965519;21739267;24232129;26514968 |
| rs1414334 | HTR2C | aripiprazole | Psychiatric Disorders | 20680028;27441116 |
| CYP2D6*41 | CYP2D6 | aripiprazole | Psychiatric Disorders | 17828532;17965519;21739267;24232129;26514968;29472872 |
| CYP2D6*2 | CYP2D6 | aripiprazole | Psychiatric Disorders | 17828532;17965519;21739267;24232129;26514968 |
| CYP2D6*1 | CYP2D6 | aripiprazole | Psychiatric Disorders | 17828532;17965519;21739267;24232129;26514968;29472872 |
| C957T | ANKK1 | aripiprazole | Psychiatric Disorders | 18855532;18926547 |
| *4/*41 and *3/*4 | CYP2D6 | aripiprazole | Psychiatric Disorders | 17828532;17965519;21739267;24232129;25868121;26514968;29472872 |
| CYP2D6*4 | CYP2D6 | aripiprazole | Psychiatric Disorders | 17828532;17965519;21739267;24232129;26514968;29472872 |
| rs1800497 | ANKK1 | aripiprazole | Psychiatric Disorders | 18855532;18926547 |
| rs1799732 | DRD2 | aripiprazole | Psychiatric Disorders | 11505224;15694263;15830237;16513877;17105675;18926547;20194480;28673279;9858029;9918131 |
| rs324420 | FAAH | aripiprazole | Psychiatric Disorders | 20631561;23799528 |
| -141C Ins/Del and Taq1A | DRD2 | aripiprazole | Psychiatric Disorders | 11505224; 11920858; 14610521; 15567074; 15694263; 15830237; 16123753; 16513877; 16973280; 17087792; 17105675; 18583979; 18687376; 18926547; 19158809; 19582781; 19900188; 20194480; 9713903; 9858029; 991813;10327430;11505224;15694263;15830237;16513877;17105675;18926547;20194480;28673279;9858029;9918131 |
| CYP2D6*5 | CYP2D6 | aripiprazole | Psychiatric Disorders | 17828532;17965519;21739267;24232129;26514968 |
| Pro129Thr | FAAH | aripiprazole | Psychiatric Disorders | 20631561;23799528 |
| rs1049353 | CNR1 | aripiprazole | Psychiatric Disorders | 20107430;20631561;23799528 |
| rs489693 | - | aripiprazole | Psychiatric Disorders | 22566560;23799528;23920449;27217270 |
| CYP3A4*22 | CYP3A4 | aripiprazole | Psychiatric Disorders | 25868121 |
| rs3813929 | HTR2C | aripiprazole | Psychiatric Disorders | 20680028 |
| CYP2D6*1xN | CYP2D6 | aripiprazole | Psychiatric Disorders | 29472872 |
| rs6280 | DRD3 | aripiprazole | Psychiatric Disorders | 19302829 |
| rs2005976 | DTNBP1 | aripiprazole | Psychiatric Disorders | 20829635 |
| rs3888190 | SH2B1 | aripiprazole | Psychiatric Disorders | 28694205 |
| CYP2D6*21 | CYP2D6 | aripiprazole | Psychiatric Disorders | 21739267 |
| rs2306987 | FAT1 | aripiprazole | Psychiatric Disorders | 19893579 |
| 1199G > A | #N/A | aripiprazole | Psychiatric Disorders | 29443543 |
| rs7412 | APOE | aripiprazole | Psychiatric Disorders | 28605058 |
| rs4305746 | TAAR6 | aripiprazole | Psychiatric Disorders | 19345712 |
| rs12970134 | - | aripiprazole | Psychiatric Disorders | 22566560 |
| CYP2D6*3 | CYP2D6 | aripiprazole | Psychiatric Disorders | 24232129 |
| rs6280 | DRD3 | aripiprazole | Psychiatric Disorders | 19302829 |
| rs2391191 | DAOA | aripiprazole | Psychiatric Disorders | 20471098 |
| rs646749 | - | aripiprazole | Psychiatric Disorders | 22566560 |
| rs1801028 | DRD2 | aripiprazole | Psychiatric Disorders | 18926547 |
| rs1050828 | G6PD | artemisinin and derivatives | Malaria | 22940027 |
| G6PD A- | G6PD | artesunate | Malaria | 20194698 |
| rs738409 | PNPLA3 | asparaginase | Cancer | 28090653;28744905 |
| rs738409 | PNPLA3 | asparaginase | Cancer | 28090653;28744905 |
| rs199695765 | CPA2 | asparaginase | Cancer | 27114598 |
| rs6021191 | NFATC2 | asparaginase | Cancer | 25987655 |
| rs7142143 | PYGL | asparaginase | Cancer | 23007406 |
| rs4958381 | - | asparaginase | Cancer | 20592726 |
| 14bp tandem repeat | ASNS | asparaginase | Cancer | 24268318 |
| HLA-DQB1*06:09 | HLA-DQB1 | aspirin | Inflammation, anti platelet drug | 15784113;19392989 |
| rs6065 | SLC25A11 | aspirin | Inflammation, anti platelet drug | 17245331;18417193 |
| 924T and 1018C alleles | GP1BA | aspirin | Inflammation, anti platelet drug | 17245331;18417193 |
| rs730012 | MGAT4B | aspirin | Inflammation, anti platelet drug | 15100686;16433794;19862937 |
| rs1126643 | ITGA2 | aspirin | Inflammation, anti platelet drug | 15205592;16214444;16493486;17157856;22940005 |
| E237G | FCER1G | aspirin | Inflammation, anti platelet drug | 18534082;18595682 |
| rs4523 | TBXA2R | aspirin | Inflammation, anti platelet drug | 17245331;23688183 |
| HLA-DPB1*03:01:01 | HLA-DPB1 | aspirin | Inflammation, anti platelet drug | 15007363;15784113;19392989;9179433 |
| rs3842787 | PTGS1 | aspirin | Inflammation, anti platelet drug | 17184645;19350112 |
| rs11587213 | FCER1G | aspirin | Inflammation, anti platelet drug | 18534082;18595682 |
| (-444)C | LTC4S | aspirin | Inflammation, anti platelet drug | 15100686;16433794;19862937 |
| DPB1*0301 | HLA-DPB1 | aspirin | Inflammation, anti platelet drug | 15007363;15784113;19392989;25372592;9179433 |
| rs1131882 | TBXA2R | aspirin | Inflammation, anti platelet drug | 15898979;27995340 |
| rs12042763 | PTGS2 | aspirin | Inflammation, anti platelet drug | 20688905 |
| rs6949799 | COL26A1 | aspirin | Inflammation, anti platelet drug | 21086123 |
| rs1613662 | GP6 | aspirin | Inflammation, anti platelet drug | 16493486 |
| rs1126510 | PTGIR | aspirin | Inflammation, anti platelet drug | 17496729 |
| rs1042136 | HLA-DPB1 | aspirin | Inflammation, anti platelet drug | 25372592 |
| rs11264580 | PEAR1 | aspirin | Inflammation, anti platelet drug | 26962983 |
| rs2071888 | TAPBP | aspirin | Inflammation, anti platelet drug | 23736108 |
| rs6809699 | MED12L | aspirin | Inflammation, anti platelet drug | 15933261 |
| rs5050 | AGT | aspirin | Inflammation, anti platelet drug | 20824505 |
| rs4292 | ACE | aspirin | Inflammation, anti platelet drug | 18727619 |
| rs2427827 | FCER1A | aspirin | Inflammation, anti platelet drug | 18595682 |
| rs1060463 | CYP4F11 | aspirin | Inflammation, anti platelet drug | 24367646 |
| HLA-DQB1*06:04:01 | HLA-DQB1 | aspirin | Inflammation, anti platelet drug | 15784113 |
| rs13431554 | IRS1 | aspirin | Inflammation, anti platelet drug | 27005817 |
| rs1061781 | B4GALT2 | aspirin | Inflammation, anti platelet drug | 27213804 |
| rs959 | PTGER3 | aspirin | Inflammation, anti platelet drug | 20587336 |
| rs7179742 | FSIP1 | aspirin | Inflammation, anti platelet drug | 20513247 |
| rs557881 | SLC6A12 | aspirin | Inflammation, anti platelet drug | 20597903 |
| rs8069732 | ITGB3 | aspirin | Inflammation, anti platelet drug | 22940005 |
| rs1050891 | HNMT | aspirin | Inflammation, anti platelet drug | 19178400 |
| rs6962291 | TBXAS1 | aspirin | Inflammation, anti platelet drug | 21449675 |
| rs1062535 | ITGA2 | aspirin | Inflammation, anti platelet drug | 17157856 |
| 1405C>T, A102A | ADORA1 | aspirin | Inflammation, anti platelet drug | 19019667 |
| rs7572857 | CEP68 | aspirin | Inflammation, anti platelet drug | 21072201 |
| rs5758 | TBXA2R | aspirin | Inflammation, anti platelet drug | 25860557 |
| rs2243250 | IL4 | aspirin | Inflammation, anti platelet drug | 20921925 |
| rs2243093 | SLC25A11 | aspirin | Inflammation, anti platelet drug | 18417193 |
| rs3856806 | PPARG | aspirin | Inflammation, anti platelet drug | 20224667 |
| HLA-DPB1*04:01:01:01 | HLA-DPB1 | aspirin | Inflammation, anti platelet drug | 9179433 |
| rs3798220 | LPA | aspirin | Inflammation, anti platelet drug | 18775538 |
| HLA-DQB1*03:02:01 | HLA-DQB1 | aspirin | Inflammation, anti platelet drug | 26366802 |
| rs1800469 | TGFB1 | aspirin | Inflammation, anti platelet drug | 19138248 |
| rs113993959 | CFTR | ataluren | Muscular dystrophy | 18722008;20622033;21233271;24836205 |
| rs75039782 | CFTR | ataluren | Muscular dystrophy | 18722008;20622033 |
| rs74767530 | CFTR | ataluren | Muscular dystrophy | 24836205 |
| CYP3A5*1A | CYP3A5 | atazanavir | Viral infection | 19710077;23033116;26892777 |
| CYP3A5*7 | CYP3A5 | atazanavir | Viral infection | 19710077;23033116;26892777 |
| rs1523130 | NR1I2 | atazanavir | Viral infection | 18831695;25151207;26892777 |
| CYP3A5*6 | CYP3A5 | atazanavir | Viral infection | 19710077;23033116;26892777 |
| rs887829 | UGT1A6 | atazanavir | Viral infection | 24557078;26180834;29117017;29737521 |
| UGT1A1*28 | UGT1A1 | atazanavir | Viral infection | 17058217;19710077;20504240;21317582;21348813;23548653;24516079;25151207 |
| UGT1A1*1 | UGT1A1 | atazanavir | Viral infection | 21317582;21348813;25151207 |
| rs8175347 | UGT1A6 | atazanavir | Viral infection | 16170755;17058217;17148966;19710077;20504240;21288825;21317582;21348813;22179231;22661571;23148286;23548653;23782005;24516079;25622064;26360703;26857335;29737521 |
| UGT1A1*6 , UGT1A1*27, UGT1A1*35 | UGT1A1 | atazanavir | Viral infection | 10591539; 11037804; 12019197; 12181419; 12181437; 12969965; 14647407; 15007088; 15280927; 15297419; 15709193; 16118329; 16170755; 16456808; 1651156; 16809730; 17058217; 17728214; 18349289; 18466101; 19364970; 19371317; 19390945; 19517472; 19852077; 20335017; 20504240; 2054240; 20562211; 22050734; 23386248; 7565971; 9653159;10381366;16170755;17058217;17148966;19710077;20504240;21288825;21317582;21348813;22179231;22661571;23148286;23548653;23782005;23886114;24516079;24557078;25151207;25622064;26180834;26360703;26857335;29117017;29737521 |
| ABCB1*2 | ABCB1 | atazanavir | Viral infection | 19710077;22394315; 11503014 |
| rs2472677 | NR1I2 | atazanavir | Viral infection | 18831695;20921307;21288825;25151207;26174719;26857335;26892777 |
| UGT1A3 (-66C) | UGT1A3 | atazanavir | Viral infection | 17058217;23782005 |
| ABCB1*1 | ABCB1 | atazanavir | Viral infection | 19710077;22394315 |
| UGT1A7 (-57G) promoter variant | UGT1A7 | atazanavir | Viral infection | 17058217;23782005 |
| TA-repeat polymorphism | UGT1A10 | atazanavir | Viral infection | 23782005 |
| rs2740574 | CYP3A4 | atazanavir | Viral infection | 22394315 |
| rs8330 | UGT1A6 | atazanavir | Viral infection | 25151207 |
| UGT1A1*6 | UGT1A1 | atazanavir | Viral infection | 20504240 |
| rs72554665 | G6PD | atazanavir | Viral infection | 22179231 |
| rs3806596 | UGT1A6 | atazanavir | Viral infection | 17058217 |
| CYP3A4*1B | CYP3A | atazanavir | Viral infection | 22394315 |
| rs776746, rs10264272 | CYP3A5 | atazanavir | Viral infection | 26892777 |
| rs292449 | NEDD4L | atenolol | Cardiovascular disease | 23353631 |
| rs2144300 | GALNT2 | atenolol | Cardiovascular disease | 24116192 |
| rs4762 | AGT | atenolol | Cardiovascular disease | 11593098 |
| rs5051 | AGT | atenolol | Cardiovascular disease | 14700505 |
| rs2106809 | ACE2 | atenolol | Cardiovascular disease | 17473847 |
| rs2932538 | CAPZA1 | atenolol | Cardiovascular disease | 23087401 |
| rs201279313 | SLC25A31 | atenolol | Cardiovascular disease | 26729753 |
| rs2514036 | ACY3 | atenolol | Cardiovascular disease | 28353407 |
| rs11124945 | PLEKHH2 | atenolol | Cardiovascular disease | 27670767 |
| rs11064426 | GNB3 | atenolol | Cardiovascular disease | 20235788 |
| rs11649514 | PRKCB | atenolol | Cardiovascular disease | 26946962 |
| 486V homozygotes | GRK4 | atenolol | Cardiovascular disease | 22949529 |
| rs340874 | PROX1 | atenolol | Cardiovascular disease | 24122840 |
| rs261316 | ALDH1A2 | atenolol | Cardiovascular disease | 29097388 |
| Ser49-Arg389 haplotype | ADRB1H1 | atenolol | Cardiovascular disease | 18615004 |
| rs1051375 | CACNA1C | atenolol | Cardiovascular disease | 20031608 |
| rs2106809 | ACE2 | atenolol | Cardiovascular disease | 17473847 |
| rs4331 | ACE | atenolol | Cardiovascular disease | 14700505 |
| rs12708954 | SLC6A2 | atomoxetine | Psychiatric Disorders | 19387424 |
| CYP2D6*94 | CYP2D6 | atomoxetine | Psychiatric Disorders | 26666748 |
| CYP2D6*7 | CYP2D6 | atomoxetine | Psychiatric Disorders | 25919121 |
| CYP2D6 poor metabolizer phenotype | CYP2D6 | atomoxetine | Psychiatric Disorders | 19387424 |
| rs3785143 | SLC6A2 | atomoxetine | Psychiatric Disorders | 23266789 |
| rs20455 | KIF6 | atorvastatin | High Cholesterol levels | 18222353;18222355;20215968;20403483;20886236 |
| rs17671591 | NA | atorvastatin | High Cholesterol levels | 20031582;26408409 |
| rs662799 | ZPR1 | atorvastatin | High Cholesterol levels | 19530961;29695967 |
| ABCG8 | ABCG8 | atorvastatin | High Cholesterol levels | 15262185;16103896;20592455 |
| (A-204C) | CYP7A1 | atorvastatin | High Cholesterol levels | 15262185;16103896;21128988;26932749 |
| rs20455 | KIF6 | atorvastatin | High Cholesterol levels | 18222353;18222355;20215968;20403483;20886236 |
| rs11887534 | ABCG8 | atorvastatin | High Cholesterol levels | 15262185;16103896;20592455 |
| rs3808607 | CYP7A1 | atorvastatin | High Cholesterol levels | 15262185;16103896;21128988;26932749 |
| rs705379 | PON1 | atorvastatin | High Cholesterol levels | 16238680;19904013 |
| rs1440451 | HTR5A | atorvastatin | High Cholesterol levels | 17600820 |
| Lys656Asn | LEPR | atorvastatin | High Cholesterol levels | 19023160 |
| rs8014194 | CLMN | atorvastatin | High Cholesterol levels | 20339536 |
| rs9370867 | MYLIP | atorvastatin | High Cholesterol levels | 25171759 |
| rs4986910 | CYP3A4 | atorvastatin | High Cholesterol levels | 16103896 |
| rs4238001 | SCARB1 | atorvastatin | High Cholesterol levels | 20064494 |
| UGT1A3*1 | UGT1A3 | atorvastatin | High Cholesterol levels | 22555810 |
| rs60282872 | SREBF1 | atorvastatin | High Cholesterol levels | 18435918 |
| SLCO1B1*1 | SLCO1B1 | atorvastatin | High Cholesterol levels | 19833260 |
| rs17367421 | FDPS | atorvastatin | High Cholesterol levels | 24311107 |
| rs12003906 | #N/A | atorvastatin | High Cholesterol levels | 20031551 |
| rs17161788 | CYP3A5 | atorvastatin | High Cholesterol levels | 18727922 |
| rs9471077 | KIF6 | atorvastatin | High Cholesterol levels | 20886236 |
| rs5925 | LDLR | atorvastatin | High Cholesterol levels | 25860945 |
| rs1800591 | MTTP | atorvastatin | High Cholesterol levels | 15864113 |
| rs10474433 | NA | atorvastatin | High Cholesterol levels | 20031582 |
| rs7552841 | PCSK9 | atorvastatin | High Cholesterol levels | 26408409 |
| rs2250242 | ORM2 | atrasentan | Cancer | 24619498 |
| rs11549465 | HIF1A | axitinib | Cancer | 25816720 |
| CDA poor metabolizers | CDA | azacitidine | Cancer | 25850965 |
| TPMT*16 | TPMT | azathioprine | Rheumatoid arthritis | 12172211;12835738;13679074;15083071;15226673;15652243;16220112;16396707;16917910;17885628;18602085;18605963;18708949;22838948;23252716;9246020;9711875;9931346 |
| TPMT*22 | TPMT | azathioprine | Rheumatoid arthritis | 12172211;12835738;13679074;15083071;15226673;15652243;16220112;16396707;16917910;17885628;18602085;18605963;18708949;22838948;23252716;9246020;9711875;9931346 |
| rs55754655 | AOX1 | azathioprine | Rheumatoid arthritis | 19500084;22495427 |
| TPMT*1S | TPMT | azathioprine | Rheumatoid arthritis | 12172211;12835738;13679074;15083071;15226673;15652243;16220112;16396707;16917910;17885628;18602085;18605963;18708949;19252404;22838948;23252716;9246020;9711875;9931346 |
| rs3765534 | ABCC4 | azathioprine | Rheumatoid arthritis | 18593894;20393862 |
| TPMT*9 | TPMT | azathioprine | Rheumatoid arthritis | 12172211;12835738;13679074;15083071;15226673;15652243;16220112;16396707;16917910;17885628;18602085;18605963;18708949;22838948;23252716;9246020;9711875;9931346 |
| TPMT*11 | TPMT | azathioprine | Rheumatoid arthritis | 12172211;12835738;13679074;15083071;15226673;15652243;16220112;16396707;16917910;17885628;18602085;18605963;18708949;22838948;23252716;9246020;9711875;9931346 |
| TPMT*14 | TPMT | azathioprine | Rheumatoid arthritis | 12172211;12835738;13679074;15083071;15226673;15652243;16220112;16396707;16917910;17885628;18602085;18605963;18708949;22838948;23252716;9246020;9711875;9931346 |
| TPMT*23 | TPMT | azathioprine | Rheumatoid arthritis | 12172211;12835738;13679074;15083071;15226673;15652243;16220112;16396707;16917910;17885628;18602085;18605963;18708949;22838948;23252716;9246020;9711875;9931346 |
| TPMT*8 | TPMT | azathioprine | Rheumatoid arthritis | 12172211;12835738;13679074;15083071;15226673;15652243;16220112;16396707;16917910;17885628;18602085;18605963;18708949;22838948;23252716;9246020;9711875;9931346 |
| TPMT*12 | TPMT | azathioprine | Rheumatoid arthritis | 12172211;12835738;13679074;15083071;15226673;15652243;16220112;16396707;16917910;17885628;18602085;18605963;18708949;22838948;23252716;9246020;9711875;9931346 |
| TPMT*17 | TPMT | azathioprine | Rheumatoid arthritis | 12172211;12835738;13679074;15083071;15226673;15652243;16220112;16396707;16917910;17885628;18602085;18605963;18708949;22838948;23252716;9246020;9711875;9931346 |
| 94C>A | ITPA | azathioprine | Rheumatoid arthritis | 21961091;26360046;26674571 |
| TPMT intermediate metabolizer phenotype | TPMT | azathioprine | Rheumatoid arthritis | 12269967;20136357;20308917 |
| TPMT*18 | TPMT | azathioprine | Rheumatoid arthritis | 12172211;12835738;13679074;15083071;15226673;15652243;16220112;16396707;16917910;17885628;18602085;18605963;18708949;22838948;23252716;9246020;9711875;9931346 |
| TPMT*20 | TPMT | azathioprine | Rheumatoid arthritis | 12172211;12835738;13679074;15083071;15226673;15652243;16220112;16396707;16917910;17885628;18602085;18605963;18708949;22838948;23252716;9246020;9711875;9931346 |
| TPMT*21 | TPMT | azathioprine | Rheumatoid arthritis | 12172211;12835738;13679074;15083071;15226673;15652243;16220112;16396707;16917910;17885628;18602085;18605963;18708949;22838948;23252716;9246020;9711875;9931346 |
| TPMT*7 | TPMT | azathioprine | Rheumatoid arthritis | 12172211;12835738;13679074;15083071;15226673;15652243;16220112;16396707;16917910;17885628;18602085;18605963;18708949;22838948;23252716;9246020;9711875;9931346 |
| TPMT*6 | TPMT | azathioprine | Rheumatoid arthritis | 12172211;12835738;13679074;15083071;15226673;15652243;16220112;16396707;16917910;17885628;18602085;18605963;18708949;22838948;23252716;9246020;9711875;9931346 |
| NUDT15*3 | NUDT15 | azathioprine | Rheumatoid arthritis | 28172964 |
| TPMT low activity | TPMT | azathioprine | Rheumatoid arthritis | 15570193 |
| rs594445 | MOCOS | azathioprine | Rheumatoid arthritis | 22495427 |
| TPMT*3A, TPMT*3B, TPMT*3C | TPMT | azathioprine | Rheumatoid arthritis | 26674571 |
| rs2834826 | RUNX1 | azathioprine | Rheumatoid arthritis | 27558924 |
| TPMT poor metabolizer phenotype | TPMT | azathioprine | Rheumatoid arthritis | 20136357 |
| TPMT*3E | TPMT | azathioprine | Rheumatoid arthritis | 23400745 |
| rs2647087 | AL662789.1 | azathioprine | Rheumatoid arthritis | 25217962 |
| rs4407290 | XDH | azathioprine | Rheumatoid arthritis | 22535280 |
| TPMT*3D | TPMT | azathioprine | Rheumatoid arthritis | 12172211 |
| rs7079 | AGT | benazepril | Hypertension | 17261659;21449848 |
| rs2106089 | SPTA1 | benazepril | Hypertension | 21449848 |
| rs2229437 | PRCP | benazepril | Hypertension | 20079160 |
| rs4253778 | PPARA | Beta Blocking Agents | Hypertension | 18855529 |
| rs800292 | CFH | bevacizumab | cancer | 22594510;22840423;23204795;23337555;23559864;23584701;23842101;24070809;26411831;26439641 |
| rs11200638 | HTRA1 | bevacizumab | cancer | 22594510;23337555;23559864;23842101 |
| rs10490924 | ARMS2 | bevacizumab | cancer | 22594510;23204795;23337555;23584701;24070809 |
| rs11200638 | HTRA1 | bevacizumab | cancer | 22594510;23337555;23559864;23842101 |
| rs2230199 | C3 | bevacizumab | cancer | 22594510;23337555;23584701 |
| rs10490924 | ARMS2 | bevacizumab | cancer | 22594510;23204795;23337555;23584701;24070809 |
| rs833069 | VEGFA | bevacizumab | cancer | 22594510;28045923 |
| rs3025033 | VEGFA | bevacizumab | cancer | 23584701;26100253 |
| rs1061170 | CFH | bevacizumab | cancer | 22594510;22840423;23204795;23337555;23559864;23584701;23842101;24070809;26411831;26439641 |
| rs2230199 | C3 | bevacizumab | cancer | 23337555;23584701 |
| rs9679290 | EPAS1 | bevacizumab | cancer | 24070809;26100253 |
| C+785T | CXCR2 | bevacizumab | cancer | 19010874 |
| rs1617640 | EPO | bevacizumab | cancer | 23584701 |
| rs2228014 | CXCR4 | bevacizumab | cancer | 27503580 |
| rs10898563 | PRSS23 | bevacizumab | cancer | 24070809 |
| rs699947 | VEGFB | bevacizumab | cancer | 20124951 |
| rs4953344 | EPAS1 | bevacizumab | cancer | 26100253 |
| rs6929249 | SLC29A1 | bevacizumab | cancer | 29871907 |
| rs2515462 | ANGPT2 | bevacizumab | cancer | 28045923 |
| rs1410996 | CFH | bevacizumab | cancer | 22594510 |
| rs5370 | EDN1 | bevacizumab | cancer | 27139155 |
| rs2230205 | C3 | bevacizumab | cancer | 22594510 |
| rs1049550 | ANXA11 | bevacizumab | cancer | 21239504 |
| rs3025000 | VEGFA | bevacizumab | cancer | 23149126 |
| rs9381299 | HSP90AB1 | bevacizumab | cancer | 29871907 |
| rs800292 | CFH | bevacizumab | cancer | 22594510;22840423;23204795;23337555;23559864;23584701;23842101;24070809;26411831;26439641 |
| rs5128 | APOC3 | bezafibrate | Billiary Cirrhosis | 12059987 |
| rs11563250 | UGT1A6 | bilirubin | liver disorder | 25778466 |
| rs11264359 | FDPS | Bisphosphonates | Bone disease, Paget's disease | 21151198;24311107 |
| rs17024608 | RBMS3 | Bisphosphonates | Bone disease, Paget's disease | 22267851 |
| rs11064477 | PTPN6 | Bisphosphonates | Bone disease, Paget's disease | 22267851 |
| rs1050565 | BLMH | bleomycin | Cancer | 18398146 |
| rs1050565 | BLMH | bleomycin | Cancer | 18398146 |
| rs4553808 | CTLA4 | bortezomib | Cancer | 21228734 |
| CYP2C9*3 | CYP2C9 | bosentan | Hypertension | 23863877;24048276;24842639 |
| CYP2C9*2 | CYP2C9 | bosentan | Hypertension | 23863877;24048276;24842639 |
| CYP2C9*2 | CYP2C9 | bosentan | Hypertension | 23863877;24048276;24842639 |
| CYP2C9*1 | CYP2C9 | bosentan | Hypertension | 23863877;24048276;24842639 |
| rs4149056, rs2306283 | SLCO1B1 | bosentan | Hypertension | 17496208;24842639 |
| rs2306283 | SLCO1B1 | bosentan | Hypertension | 24842639 |
| rs3957357 | GSTA1 | brostallicin | Cancer | 24215845 |
| HLA-DRB1*08:02:01 | HLA-DRB1 | bucillamine | Inflammation | 24899791 |
| CYP2D6*50 | CYP2D6 | bufuralol | Hypertension, cardiovascular diseases | 11470994;11950793;12152006;17470523;18784265;19158312;24647041;25469868;26310775;7935325;8946471;9089660;9415713 |
| CYP2D6*54 | CYP2D6 | bufuralol | Hypertension, cardiovascular diseases | 11470994;11950793;12152006;17470523;18784265;19158312;24647041;25469868;26310775;7935325;8946471;9089660;9415713 |
| CYP2D6*37 | CYP2D6 | bufuralol | Hypertension, cardiovascular diseases | 11470994;11950793;12152006;17470523;18784265;19158312;24647041;25469868;26310775;7935325;8946471;9089660;9415713 |
| CYP2D6*48 | CYP2D6 | bufuralol | Hypertension, cardiovascular diseases | 11470994;11950793;12152006;17470523;18784265;19158312;24647041;25469868;26310775;7935325;8946471;9089660;9415713 |
| CYP2D6*51 | CYP2D6 | bufuralol | Hypertension, cardiovascular diseases | 11470994;11950793;12152006;17470523;18784265;19158312;24647041;25469868;26310775;7935325;8946471;9089660;9415713 |
| CYP2D6*72 | CYP2D6 | bufuralol | Hypertension, cardiovascular diseases | 11470994;11950793;12152006;17470523;18784265;19158312;24647041;25469868;26310775;7935325;8946471;9089660;9415713 |
| CYP2D6*49 | CYP2D6 | bufuralol | Hypertension, cardiovascular diseases | 11470994;11950793;12152006;17470523;18784265;19158312;24647041;25469868;26310775;7935325;8946471;9089660;9415713 |
| CYP2D6*27 | CYP2D6 | bufuralol | Hypertension, cardiovascular diseases | 11470994;11950793;12152006;17470523;18784265;19158312;24647041;25469868;26310775;7935325;8946471;9089660;9415713 |
| CYP2D6*62 | CYP2D6 | bufuralol | Hypertension, cardiovascular diseases | 11470994;11950793;12152006;17470523;18784265;19158312;24647041;25469868;26310775;7935325;8946471;9089660;9415713 |
| CYP2D6*55 | CYP2D6 | bufuralol | Hypertension, cardiovascular diseases | 11470994;11950793;12152006;17470523;18784265;19158312;24647041;25469868;26310775;7935325;8946471;9089660;9415713 |
| CYP2D6*53 | CYP2D6 | bufuralol | Hypertension, cardiovascular diseases | 11470994;11950793;12152006;17470523;18784265;19158312;24647041;25469868;26310775;7935325;8946471;9089660;9415713 |
| CYP2D6*40 | CYP2D6 | bufuralol | Hypertension, cardiovascular diseases | 11470994;11950793;12152006;17470523;18784265;19158312;24647041;25469868;26310775;7935325;8946471;9089660;9415713 |
| CYP2D6*14B | CYP2D6 | bufuralol | Hypertension, cardiovascular diseases | 11470994;11950793;12152006;17470523;18784265;19158312;24647041;25469868;26310775;7935325;8946471;9089660;9415713 |
| CYP2D6*39 | CYP2D6 | bufuralol | Hypertension, cardiovascular diseases | 11470994;11950793;12152006;17470523;18784265;19158312;24647041;25469868;26310775;7935325;8946471;9089660;9415713 |
| CYP2D6*57 | CYP2D6 | bufuralol | Hypertension, cardiovascular diseases | 11470994;11950793;12152006;17470523;18784265;19158312;24647041;25469868;26310775;7935325;8946471;9089660;9415713 |
| CYP2D6*18 | CYP2D6 | bufuralol | Hypertension, cardiovascular diseases | 11470994;11950793;12152006;17470523;18784265;19158312;24647041;25469868;26310775;7935325;8946471;9089660;9415713 |
| CYP2D6*47 | CYP2D6 | bufuralol | Hypertension, cardiovascular diseases | 11470994;11950793;12152006;17470523;18784265;19158312;24647041;25469868;26310775;7935325;8946471;9089660;9415713 |
| CYP2D6*71 | CYP2D6 | bufuralol | Hypertension, cardiovascular diseases | 11470994;11950793;12152006;17470523;18784265;19158312;24647041;25469868;26310775;7935325;8946471;9089660;9415713 |
| CYP2D6*75 | CYP2D6 | bufuralol | Hypertension, cardiovascular diseases | 11470994;11950793;12152006;17470523;18784265;19158312;24647041;25469868;26310775;7935325;8946471;9089660;9415713 |
| CYP2D6*12 | CYP2D6 | bufuralol | Hypertension, cardiovascular diseases | 11470994;11950793;12152006;17470523;18784265;19158312;24647041;25469868;26310775;7935325;8946471;9089660;9415713 |
| rs745746329 | CYP2D6 | bufuralol | Hypertension, cardiovascular diseases | 26310775 |
| rs28371703 | CYP2D6 | bufuralol | Hypertension, cardiovascular diseases | 2211621 |
| rs28371704 | CYP2D6 | bufuralol | Hypertension, cardiovascular diseases | 2211621 |
| rs1529927 | SLC12A3 | bumetanide | Hypertension | 17460608;20877298 |
| rs34902660 | SLC17A3 | bumetanide | Hypertension | 21282933 |
| rs5723 | SCNN1G | bumetanide | Hypertension | 17460608 |
| rs529520 | OPRD1 | buprenorphine | Pain symptom | 23612435;24126707 |
| rs581111 | OPRD1 | buprenorphine | Pain symptom | 23612435;24126707 |
| rs678849 | OPRD1 | buprenorphine | Pain symptom | 23612435 |
| rs7438135 | UGT2B7 | buprenorphine | Pain symptom | 19841060 |
| rs10485058 | OPRM1 | buprenorphine | Pain symptom | 27958381 |
| CYP2B6*18 | CYP2B6 | bupropion | Psychiatric Disorders | 14515060;15083067;15722458;23149928;23344581;26580670;29756345 |
| CYP2B6*4 | CYP2B6 | bupropion | Psychiatric Disorders | 14515060;15083067;15722458;23149928;23344581;26580670;29756345 |
| CYP2B6*2 | CYP2B6 | bupropion | Psychiatric Disorders | 14515060;15083067;15722458;23149928;23344581;26580670;29756345 |
| CYP2B6*22 | CYP2B6 | bupropion | Psychiatric Disorders | 14515060;15083067;15722458;23149928;23344581;26580670;29756345 |
| CYP2B6*5 | CYP2B6 | bupropion | Psychiatric Disorders | 14515060;15083067;15722458;23149928;23344581;26580670;29756345 |
| rs12721655 | CYP2B6 | bupropion | Psychiatric Disorders | 21659470 |
| rs2742417, rs2251954, rs2742421, rs2742423, rs1969624, rs2673057, rs2742431, rs2742435, rs2245705, rs2742390 | SACM1L | bupropion | Psychiatric Disorders | 22041458 |
| rs6702335 | EPB41 | bupropion | Psychiatric Disorders | 21808284 |
| rs11746641 | DRD1 | bupropion | Psychiatric Disorders | 22495174 |
| rs2056527 | - | bupropion | Psychiatric Disorders | 24733007 |
| rs2013249 | AKR7A3 | bupropion | Psychiatric Disorders | 28685396 |
| rs2742435 | SACM1L | bupropion | Psychiatric Disorders | 22041458 |
| rs12021667, rs12027267, rs6702335, rs12039988 | EPB41 | bupropion | Psychiatric Disorders | 21808284 |
| CYP2B6*6B | CYP2B6 | bupropion | Psychiatric Disorders | 15083067 |
| rs2717162 | GALR1 | bupropion | Psychiatric Disorders | 22373943 |
| rs2868177 | POR | bupropion | Psychiatric Disorders | 26580670 |
| rs1908557 | AC093866.1 | bupropion | Psychiatric Disorders | 27622933 |
| rs3957356 | GSTA1 | busulfan | Cancer | 26419450 |
| rs1021737 | CTH | busulfan | Cancer | 27779248 |
| rs4148405 | ABCC3 | busulfan | Cancer | 23677058 |
| rs4715354 and rs7746993 | GSTA5 | busulfan | Cancer | 24192531 |
| rs7141505 | SLC7A8 | busulfan | Cancer | 24192117 |
| rs6070697, rs463312 | TUBB1 | cabazitaxel | Cancer | 27020167 |
| CYP1A2*1F | CYP1A2 | caffeine | Psychiatric disorder, mental alertness, migraine | 11755554;15849225;17370067 |
| rs2069514 | CYP1A2 | caffeine | Psychiatric disorder, mental alertness, migraine | 10101295;21593735;29282363 |
| CYP1A2*1K | CYP1A2 | caffeine | Psychiatric disorder, mental alertness, migraine | 12920202 |
| rs8192766 | CYP2E1 | caffeine | Psychiatric disorder, mental alertness, migraine | 18798002 |
| rs5760405 | SPECC1L | caffeine | Psychiatric disorder, mental alertness, migraine | 20520601 |
| rs1110976 | DRD2 | caffeine | Psychiatric disorder, mental alertness, migraine | 18305461 |
| rs2198843 | - | caffeine | Psychiatric disorder, mental alertness, migraine | 18798002 |
| rs35694136 | CYP1A2 | caffeine | Psychiatric disorder, mental alertness, migraine | 18759349 |
| rs4410790 | AHR | caffeine | Psychiatric disorder, mental alertness, migraine | 21490707 |
| rs11023374 | CYP2R1 | calcidiol | Bone density conservation agent | 26661839 |
| rs2278294 | IMPDH1 | Calcineurin inhibitors | Atopic dermatitis. | 22960765 |
| rs118192167 | RYR1 | calcium | Bone disease | 11063719 |
| rs2239128 | CACNA1C | calcium channel blockers | Hypertension | 16610939 |
| rs10898815 | NUMA1 | calcium channel blockers | Hypertension | 24192120 |
| rs3758785 | GPR83 | candesartan | Hypertension | 22566498 |
| rs3758785 | GPR83 | candesartan | Hypertension | 22566498 |
| rs602950 | CDA | capecitabine | Cancer | 23736036;28139840 |
| rs3215400 | CDA | capecitabine | Cancer | 18473752;21325291;23736036 |
| rs532545 | CDA | capecitabine | Cancer | 21325291;23736036;28139840 |
| rs11075646 | CES2 | capecitabine | Cancer | 18473752;23588952 |
| rs576523 | AL121985.3 | capecitabine | Cancer | 25815774 |
| rs1048977 | CDA | capecitabine | Cancer | 28347776 |
| rs7187684 | CES1P1 | capecitabine | Cancer | 28139840 |
| rs191934521 | KIAA0922 | capecitabine | Cancer | 28715540 |
| rs7548189 | DPYD | capecitabine | Cancer | 24647007 |
| rs2290272 | SLC28A1 | capecitabine | Cancer | 23588952 |
| rs291592 | DPYD | capecitabine | Cancer | 28481884 |
| rs9937 | RRM1 | capecitabine | Cancer | 22026922 |
| rs8192924 | CES2 | capecitabine | Cancer | 18473752 |
| rs1800440 | RMDN2 | capecitabine | Cancer | 22026922 |
| rs9936750 | - | capecitabine | Cancer | 24595012 |
| p.Gln334Arg, p.Arg465Ter | DPYS | capecitabine | Cancer | 25915935 |
| rs2279020 | GABRA1 | carbamazepine | Seizures, Epilepsy | 21747585;24061200 |
| rs211037 | GABRG2 | carbamazepine | Seizures, Epilepsy | 21747585;24061200 |
| HLA-A*02:01:01:01 | HLA-A | carbamazepine | Seizures, Epilepsy | 21917426;22211527;23830818 |
| rs2290732 | GABRA1 | carbamazepine | Seizures, Epilepsy | 21747585;22591328;24061200;24236484 |
| rs1051740 | EPHX1 | carbamazepine | Seizures, Epilepsy | 15692831;19620853;22188362;23252947;26314341;26555147 |
| HLA-B*15:11:01 | HLA-B | carbamazepine | Seizures, Epilepsy | 21204807;21917426;22348435;24336023 |
| rs2234922 | EPHX1 | carbamazepine | Seizures, Epilepsy | 15692831;19620853;22188362;23252947;25495409;26555147 |
| c.588C>T | GABRG2 | carbamazepine | Seizures, Epilepsy | 21747585;24061200 |
| rs2071197 | HNF4A | carbamazepine | Seizures, Epilepsy | 24384557;28837897 |
| HLA-B*15:21 | HLA-B | carbamazepine | Seizures, Epilepsy | 28358139;29053440 |
| rs28365062 | UGT2B7 | carbamazepine | Seizures, Epilepsy | 22188362 |
| rs4688040 | NR1I2 | carbamazepine | Seizures, Epilepsy | 23252947 |
| rs4828696 | GABRA3 | carbamazepine | Seizures, Epilepsy | 24236484 |
| HLA-A*74:01 | HLA-A | carbamazepine | Seizures, Epilepsy | 21244392 |
| HLA-DRB1*03:01:01:01 | HLA-DRB1 | carbamazepine | Seizures, Epilepsy | 23830818 |
| 1911 C/G, +438 C/T | HSPA1A | carbamazepine | Seizures, Epilepsy | 16538175 |
| rs3130690 | HLA-B | carbamazepine | Seizures, Epilepsy | 16538176 |
| rs2687116 | CYP3A4 | carbamazepine | Seizures, Epilepsy | 28343093 |
| rs3738046 | EPHX1 | carbamazepine | Seizures, Epilepsy | 25495409 |
| rs4646440 | CYP3A4 | carbamazepine | Seizures, Epilepsy | 26421491 |
| rs3219151 | GABRA6 | carbamazepine | Seizures, Epilepsy | 24061200 |
| rs3740067 | ABCC2 | carbamazepine | Seizures, Epilepsy | 24567120 |
| rs2290732 | GABRA1 | carbamazepine | Seizures, Epilepsy | 22591328 |
| rs1633021 | - | carbamazepine | Seizures, Epilepsy | 21149285 |
| HLA-C*07:04:01 | HLA-C | carbamazepine | Seizures, Epilepsy | 19694795 |
| HLA-B*38:02:01 | HLA-B | carbimazole | Antithyroid agent | 26151496;26599303 |
| rs2494752 | ZBTB42 | carboplatin | Cancer | 22901187 |
| rs1656402 | EIF4E2 | carboplatin | Cancer | 21079520 |
| rs17309872 | ACSS2 | carboplatin | Cancer | 21636554 |
| rs2849380 | BCL2 | carboplatin | Cancer | 23963862 |
| rs1453542 | OR4D6 | carboplatin | Cancer | 26378035 |
| rs1801368 | MAD1L1 | carboplatin | Cancer | 23407047 |
| rs3803304 | AKT1 | carboplatin | Cancer | 20447721 |
| rs16886403 | MAP3K1 | carboplatin | Cancer | 21636554 |
| rs3740556 | EIF3A | carboplatin | Cancer | 23127338 |
| Gln27 | ADRB1H3 | carvedilol | Hypertension | 21599570 |
| rs12233719 | UGT2B7 | carvedilol | Hypertension | 17329852 |
| Arg389Gly | ADRB1 | catecholamines | Cardiovascular disease, allergy | 17541557 |
| T44M (c.131C>T), T400I (c.1199C>T) | SLC22A3 | catecholamines | Cardiovascular disease, allergy | 20859243 |
| rs11568482 | SLC22A8 | cefotaxime | Infection | 23649425 |
| CACNA1I | CACNA1I | celecoxib | Inflammation | 22336956 |
| CYP2C19*14 | CYP2C19 | celecoxib | Inflammation | 21692664 |
| BCAR1 | BCAR1 | celecoxib | Inflammation | 22336956 |
| CDKN1A | CDKN1A | celecoxib | Inflammation | 22336956 |
| rs7518660 | IL23R | celecoxib | Inflammation | 23778325 |
| rs112445441 | KRAS | cetuximab | Cancer | 20978259;22537608;22734028;23071293;23090619;26371285;26623049;26812186 |
| rs4444903 | EGF | cetuximab | Cancer | 16788380;18349392;21673069;23959273;27897268 |
| A870G | CCND1 | cetuximab | Cancer | 16788380;18349392;22117530 |
| L858R | EGFR | cetuximab | Cancer | 12648464; 14990632; 14990633; 16257339; 16377102; 16467544; 18003960; 18316547; 18375904; 18681783; 18784101; 19339720; 20551942; 22594511; 23022519; 6298788;11255078;16788380;22486600;23959273;24513691;28031227 |
| rs61764370 | KRAS | cetuximab | Cancer | 23324806;24727325;25183481;25210463;26162609;27897268 |
| rs9344 | CCND1 | cetuximab | Cancer | 16788380;18349392;22117530 |
| rs2227983 | EGFR | cetuximab | Cancer | 16788380;23959273;28031227 |
| p.G13D | KRAS | cetuximab | Cancer | 20978259;22537608;22734028;23071293;23090619;23324806;24727325;25183481;25210463;26162609;26371285;26438111;26623049;26812186;27897268 |
| A61G | EGF | cetuximab | Cancer | 16788380;18349392;21673069;23959273;27897268 |
| rs712830 | EGFR | cetuximab | Cancer | 22486600 |
| rs3913032 | - | cetuximab | Cancer | 23959273 |
| rs885036 | MGAT4A | cetuximab | Cancer | 26222057 |
| rs121913529 | KRAS | cetuximab | Cancer | 22734028 |
| exons 2 (codons 12 and 13), 3 (codons 59 and 61), and 4 (codons 117 and 146) | NRAS | cetuximab | Cancer | 26438111 |
| rs11568315 | EGFR | cetuximab | Cancer | 24513691 |
| rs1570360 | VEGFA | cetuximab | Cancer | 26615857 |
| rs186983396 | RYR1 | chlorocresol | Infection | 29344738 |
| rs118204423 | RYR1 | chlorocresol | Infection | 22415532 |
| rs61824877 | LINC00862 | chlorothiazide | Hypertension | 29523524 |
| rs1415744 | EPM2A | chlorpromazine | Psychiatric Disorders | 27092952 |
| rs3025058 | MMP3 | chlorthalidone | Hypertension | 21183746 |
| rs36210421 | KCNH2 | cisapride | Gastrointestinal agent | 14975928 |
| rs1805123 | KCNH2 | cisapride | Gastrointestinal agent | 14975928 |
| rs2075252 | LRP2 | cisplatin | cancer | 17457342;23274376 |
| rs1051640 | ABCC3 | cisplatin | cancer | 23588304;25141953;27457817;28448657 |
| rs2075252 | LRP2 | cisplatin | cancer | 17457342;23274376 |
| intron 6 AGG/AGG genotype | GSTM3 | cisplatin | cancer | 19786980;22188361 |
| rs4148737 | ABCB1 | cisplatin | cancer | 22016816;29938344 |
| rs10981694 | FKBP15 | cisplatin | cancer | 22516052;28448657 |
| rs1872328 | ACYP2 | cisplatin | cancer | 25665007;26928270;28445188 |
| rs12201199 | TPMT | cisplatin | cancer | 19898482;23588304;23820299;24642735;25141953;25551397;28445188;28448657 |
| rs4646316 | COMT | cisplatin | cancer | 19898482;23588304;23820299;25141953;25551397;28445188;28448657;28560854 |
| rs25489 | XRCC1 | cisplatin | cancer | 21902499;22188361 |
| rs9332377 | COMT | cisplatin | cancer | 19898482;23588304;23820299;24642735;25141953;25551397;28445188;28448657;28560854 |
| rs1799735 | 0 | cisplatin | cancer | 19786980;22188361 |
| rs10276036 | ABCB1 | cisplatin | cancer | 22016816;29938344 |
| rs6848982 | ABHD18 | cisplatin | cancer | 21902499 |
| rs11226 | RAD52 | cisplatin | cancer | 24533712 |
| rs232043 | RRM1 | cisplatin | cancer | 21642870 |
| rs2228171 | LRP2 | cisplatin | cancer | 23274376 |
| rs465646 | REV3L | cisplatin | cancer | 25748439 |
| rs244898 | RARS | cisplatin | cancer | 27150640 |
| rs4788863 | SLC16A5 | cisplatin | cancer | 28448657 |
| rs2166219 | - | cisplatin | cancer | 23478653 |
| 10920CG | UBE2I | cisplatin | cancer | 19859084 |
| rs2804402 | ABCC2 | cisplatin | cancer | 22534871 |
| rs60369023 | CDA | cisplatin | cancer | 15814642 |
| rs2720376 | CASP3 | cisplatin | cancer | 25829401 |
| rs6721961 | NFE2L2 | cisplatin | cancer | 27457817 |
| rs4148416 | ABCC3 | cisplatin | cancer | 22016816 |
| rs7483 | GSTM3 | cisplatin | cancer | 22188361 |
| rs77124181, rs2291767 | OTOS | cisplatin | cancer | 25410892 |
| rs2284449 | RRM1 | cisplatin | cancer | 21642870 |
| Asn118Asn | LARP1B | cisplatin | cancer | 21902499 |
| rs560018 | GSTM4 | cisplatin | cancer | 20200426 |
| 7632T>A, Ile105Val | CYP2E1, GSTP1 | cisplatin | cancer | 22188361 |
| rs7103411 | BDNF | citalopram | Psychiatric Disorders, Major Depression | 19236730;22965830;29748862 |
| rs7124442 | BDNF | citalopram | Psychiatric Disorders, Major Depression | 19236730;22965830 |
| rs25531 | SLC6A4 | citalopram | Psychiatric Disorders, Major Depression | 18618621;19996755;23973251 |
| rs25531 | SLC6A4 | citalopram | Psychiatric Disorders, Major Depression | 17606812;19996755 |
| rs1800532 | TPH1 | citalopram | Psychiatric Disorders, Major Depression | 19590397;23221997;23733030 |
| rs2709376, rs2253206, rs7569963, rs7594560, and rs4675690 | CREB1 | citalopram | Psychiatric Disorders, Major Depression | 17548750;20643483 |
| rs7569963 | METTL21A | citalopram | Psychiatric Disorders, Major Depression | 17548750;20643483 |
| rs25531C | SLC6A4 | citalopram | Psychiatric Disorders, Major Depression | 17606812;19996755 |
| rs6966038 | AC006967.1 | citalopram | Psychiatric Disorders, Major Depression | 19846067 |
| rs2518224 | GRIK2 | citalopram | Psychiatric Disorders, Major Depression | 17898344 |
| rs520210 | NEDD4L | citalopram | Psychiatric Disorders, Major Depression | 23809733 |
| rs1386494 | TPH2 | citalopram | Psychiatric Disorders, Major Depression | 19590397 |
| rs17135437 | COL26A1 | citalopram | Psychiatric Disorders, Major Depression | 22760553 |
| rs334558 | GSK3B | citalopram | Psychiatric Disorders, Major Depression | 18195729 |
| rs28401781 | ABCB1 | citalopram | Psychiatric Disorders, Major Depression | 24192121 |
| rs2227631 | SERPINE1 | citalopram | Psychiatric Disorders, Major Depression | 18794724 |
| rs929493 | SLC18A2 | citalopram | Psychiatric Disorders, Major Depression | 18797399 |
| rs10903034 | PAPLN | citalopram | Psychiatric Disorders, Major Depression | 19724244 |
| rs495794, rs153549, rs153560 | REEP5 | citalopram | Psychiatric Disorders, Major Depression | 22795047 |
| rs363390 | AL731557.1 | citalopram | Psychiatric Disorders, Major Depression | 18797399 |
| rs1006737 | CACNA1C | citalopram | Psychiatric Disorders, Major Depression | 19388002 |
| rs2270007 | CRHR2 | citalopram | Psychiatric Disorders, Major Depression | 17467808 |
| rs585719 | LINC01198 | citalopram | Psychiatric Disorders, Major Depression | 23158458 |
| rs4675690 | - | citalopram | Psychiatric Disorders, Major Depression | 17548750 |
| rs4570625 | TPH2 | citalopram | Psychiatric Disorders, Major Depression | 23510446 |
| rs6296 | HTR1B | citalopram | Psychiatric Disorders, Major Depression | 27324805 |
| rs1374385 | PER2 | citalopram | Psychiatric Disorders, Major Depression | 19481584 |
| HLA-DRB5*01:01:01 | HLA-DRB3 | clavulanate | Infection | 10535882 |
| HLA-B*18:01:01 | HLA-B | clavulanate | Infection | 23874514 |
| rs9274407 | HLA-DQB1 | clavulanate | Infection | 21570397 |
| c.3184 A> G | SCN1A | clobazam | Seizures, Epilepsy | 21747585;23859570;25155934;28753467 |
| rs2298771 | SCN1A | clobazam | Seizures, Epilepsy | 21747585;23859570;25155934;28753467 |
| rs1057868 | POR | clobazam | Seizures, Epilepsy | 24345815 |
| -511 CC | IL1B | clodronate | Bone disease, Paget's disease | 16257277 |
| rs1080985 | CYP2D6 | clomipramine | Psychiatric Disorders, Major Depression | 27800629 |
| rs6113 | SERPINA5 | clomipramine | Psychiatric Disorders, Major Depression | 21625751 |
| rs29000568 | ADRA2B | clonidine | Hypertension | 20833658 |
| CYP2C19*23 | CYP2C19 | clopidogrel | heart attack, stroke | 24945780;25001882 |
| rs28399504 | CYP2C19 | clopidogrel | heart attack, stroke | 19106083;19106084;19463375;20801498;20965456;21358751;26265231 |
| CYP2C19*5 | CYP2C19 | clopidogrel | heart attack, stroke | 19106083;19106084;20801498;21345843;21511217;21689142;21806387;22007612;22374717;22464343;22723959;22955794;22974728;23001453;23150151;23428009;23429358;23517020;23556336;23640828;23692149;23726091;23809542;24019397;24019752;24080325;24088578;24214141;24262617;24336898;24353446;24402637;24418943;24535487;24710841;24762860;24782221;24821368;24856643;24996381;25001882;25258374;25329996;25529343;25828136;25850030;25851472;25877345;26071277;26265231;26526111;29102571;29653637; 23470885 |
| CYP2C19*22 | CYP2C19 | clopidogrel | heart attack, stroke | 24945780;25001882 |
| CYP2C19*26 | CYP2C19 | clopidogrel | heart attack, stroke | 24945780;25001882 |
| P2RY12 H1 | P2RY12 | clopidogrel | heart attack, stroke | 21349573;27756942 |
| CYP2C19*28 | CYP2C19 | clopidogrel | heart attack, stroke | 24945780;25001882 |
| rs16846673 | MED12L | clopidogrel | heart attack, stroke | 19106083;26362473 |
| CYP2C19*24 | CYP2C19 | clopidogrel | heart attack, stroke | 24945780;25001882 |
| rs9859538 | MED12L | clopidogrel | heart attack, stroke | 27450232;28091702 |
| P2RY12 F | P2RY12 F | clopidogrel | heart attack, stroke | 20031628 |
| rs6798637 | MED12L | clopidogrel | heart attack, stroke | 27450232 |
| rs12456693, rs2487032 | SLC14A2 | clopidogrel | heart attack, stroke | 27981573 |
| rs16863323 | MED12L | clopidogrel | heart attack, stroke | 28091702 |
| CYP2C19*2D | CYP2C19 | clopidogrel | heart attack, stroke | 24504666 |
| rs659366 | UCP2 | clopidogrel | heart attack, stroke | 23621569 |
| Thr715Pro | CD62p | clopidogrel | heart attack, stroke | 15968882 |
| rs11568732 | CYP2C19 | clopidogrel | heart attack, stroke | 29260275 |
| rs71647872 | 0 | clopidogrel | heart attack, stroke | 23275066 |
| CYP2C9*8 | CYP2C9 | clopidogrel | heart attack, stroke | 24762860 |
| CYP2C19 extensive metabolizers | CYP2C19 | clopidogrel | heart attack, stroke | 24262617 |
| rs3785161 | CES1P1 | clopidogrel | heart attack, stroke | 24535487 |
| rs1934980 | CYP2C8 | clopidogrel | heart attack, stroke | 26961113 |
| CYP2C19 intermediate metabolizer genotype | CYP2C19 | clopidogrel | heart attack, stroke | 20978260 |
| rs3732759 | MED12L | clopidogrel | heart attack, stroke | 27566695 |
| rs2134688 | ARNT | clopidogrel | heart attack, stroke | 24353446 |
| rs2307240 | CES1 | clopidogrel | heart attack, stroke | 28775293 |
| rs1330344 | PTGS1 | clopidogrel | heart attack, stroke | 26870959 |
| rs4793665 | ABCC3 | clopidogrel | heart attack, stroke | 23692149 |
| rs187941554 | RAD18 | clopidogrel active metabolite | heart attack, stroke | 28207573 |
| rs686 | DRD1 | clozapine | Psychiatric Disorders, Major Depression | 17092969;21332319 |
| rs1062613 | HTR3A | clozapine | Psychiatric Disorders, Major Depression | 20168265;22700043 |
| rs2276302 | HTR3A | clozapine | Psychiatric Disorders, Major Depression | 20168265;22700043 |
| rs518147 | HTR2C | clozapine | Psychiatric Disorders, Major Depression | 17016522;17632216;19434072;21121776;21994515;22967772;25152019;27217270 |
| rs806378 | CNR1 | clozapine | Psychiatric Disorders, Major Depression | 20107430;23799528 |
| rs265981 | DRD1 | clozapine | Psychiatric Disorders, Major Depression | 17092969 |
| rs2514218 | DRD2 | clozapine | Psychiatric Disorders, Major Depression | 26666695 |
| rs2333227 | MPO | clozapine | Psychiatric Disorders, Major Depression | 27168101 |
| rs3766522 | PRKAB2 | clozapine | Psychiatric Disorders, Major Depression | 22305490 |
| rs3780412 | SLC1A1 | clozapine | Psychiatric Disorders, Major Depression | 19884611 |
| rs4567028 | GFRA2 | clozapine | Psychiatric Disorders, Major Depression | 20116071 |
| rs8087522 | MC4R | clozapine | Psychiatric Disorders, Major Depression | 22310352 |
| rs742105 | DTNBP1 | clozapine | Psychiatric Disorders, Major Depression | 19369910 |
| rs11146020 | GRIN1 | clozapine | Psychiatric Disorders, Major Depression | 21332319 |
| rs9852 | TBC1D1 | clozapine | Psychiatric Disorders, Major Depression | 23364847 |
| rs2277984 | C3 | clozapine | Psychiatric Disorders, Major Depression | 26503818 |
| rs149104283 | NA | clozapine | Psychiatric Disorders, Major Depression | 27400856 |
| rs1800234 | PPARA | clozapine | Psychiatric Disorders, Major Depression | 21994515 |
| rs472660 | CYP3A43 | clozapine | Psychiatric Disorders, Major Depression | 25150845 |
| rs13064530 | HRH1 | clozapine | Psychiatric Disorders, Major Depression | 22722500 |
| rs1079598 | DRD2 | clozapine | Psychiatric Disorders, Major Depression | 20714340 |
| rs72547517 | CYP1A2 | clozapine | Psychiatric Disorders, Major Depression | 20125119 |
| rs1072388 | GRIN2B | clozapine | Psychiatric Disorders, Major Depression | 26876050 |
| rs10465180 | NTRK2 | clozapine | Psychiatric Disorders, Major Depression | 25751398 |
| rs11872992 | MC4R | clozapine | Psychiatric Disorders, Major Depression | 22310352 |
| rs2929183 | CCKBR | clozapine | Psychiatric Disorders, Major Depression | 20732371 |
| HLA-DRB5*02:02 | HLA-DRB3 | clozapine | Psychiatric Disorders, Major Depression | 11146763 |
| rs72547516 | CYP1A2 | clozapine | Psychiatric Disorders, Major Depression | 20125119 |
| rs11146020 | GRIN1 | clozapine | Psychiatric Disorders, Major Depression | 21332319 |
| HLA-DRB3*02:02:01:01 | HLA-DRB3 | clozapine | Psychiatric Disorders, Major Depression | 11266078 |
| HLA-DQB1*03:01:01:01 | HLA-DQB1 | clozapine | Psychiatric Disorders, Major Depression | 2222133 |
| rs2535629 | ITIH3 | clozapine | Psychiatric Disorders, Major Depression | 27396837 |
| rs4436578 | DRD2 | clozapine | Psychiatric Disorders, Major Depression | 20375926 |
| C-G-Cys23, -759C allele | HTR2C | clozapine | Psychiatric Disorders, Major Depression | 21121776 |
| rs2740204 | AVP | clozapine | Psychiatric Disorders, Major Depression | 20196918 |
| Pro200Leu | GPX1 | clozapine | Psychiatric Disorders, Major Depression | 19946932 |
| rs806368 | CNR1 | cocaine | Psychiatric disorder | 21790903 |
| rs62638690 | OPRM1 | cocaine | Psychiatric disorder | 23454283 |
| rs684513 | CHRNA5 | cocaine | Psychiatric disorder | 20485328 |
| rs4263329 | BCHE | cocaine | Psychiatric disorder | 24312228 |
| CYP2D6*2A | CYP2D6 | codeine | Pain symptom | 16920476;18719619 |
| rs61737947 | 0 | codeine | Pain symptom | 18838503 |
| CYP2D6*1,*10,*17, *2, *3, *4, *40, *41, *5, *6 | CYP2D6 | codeine | Pain symptom | 22492761,24122716,18334103,10850391,9357098, 22398969 |
| rs1876828 | CRHR1 | corticosteroids | Inflammation, arthritis, asthma | 15128701;18539200;19210659;22248724;22370858;22641026;27523435 |
| rs242941 | CRHR1 | corticosteroids | Inflammation, arthritis, asthma | 15128701;27523435 |
| rs1042713 | ADRB2 | corticosteroids | Inflammation, arthritis, asthma | 19932356;24279851;26774659 |
| rs242 | CRHR1 | corticosteroids | Inflammation, arthritis, asthma | 15128701;18539200;19210659;22248724;22370858;22641026;27523435 |
| rs3213729 | DCAF4 | corticosteroids | Inflammation, arthritis, asthma | 22366774 |
| rs4713916 | FKBP5 | corticosteroids | Inflammation, arthritis, asthma | 21788965 |
| GLCCI1 | GLCCI1 | corticosteroids | Inflammation, arthritis, asthma | 27523435 |
| rs138335 | ST13 | corticosteroids | Inflammation, arthritis, asthma | 25616159 |
| rs1741981 | LCK | corticosteroids | Inflammation, arthritis, asthma | 24307847 |
| rs2241843 | BCL2L11 | corticosteroids | Inflammation, arthritis, asthma | 29282361 |
| rs28364072 | FCER2 | corticosteroids | Inflammation, arthritis, asthma | 21958076 |
| CYP3A7*1C | CYP3A7 | cortisone acetate | Inflammation | 21915484 |
| rs13253389 | NAT1 | cotinine | Psychiatric disorders | 25156213 |
| rs77107237 | AC021146.11 | cotinine | Psychiatric disorders | 26833182 |
| rs12209447 | OPRM1 | cotinine | Psychiatric disorders | 23223006 |
| CYP2A6*17 | CYP2A6 | cotinine | Psychiatric disorders | 23714690 |
| rs2942857 | UGT2B10 | cotinine | Psychiatric disorders | 28178031 |
| rs563649 | OPRM1 | cotinine | Psychiatric disorders | 23223006 |
| rs28399433 | CYP2A6 | coumarin | Blood clots, thrombosis, stroke | 11394901;16041240;21521021 |
| rs1801272 | CYP2A6 | coumarin | Blood clots, thrombosis, stroke | 9409631;9827545 |
| CYP2A6*9A | CYP2A6 | coumarin | Blood clots, thrombosis, stroke | 12844137;14583682 |
| CYP2A6*19 | CYP2A6 | coumarin | Blood clots, thrombosis, stroke | 15900015 |
| CYP2A6*20 | CYP2A6 | coumarin | Blood clots, thrombosis, stroke | 15993850 |
| CYP2A6*12A | CYP2A6 | coumarin | Blood clots, thrombosis, stroke | 12325023 |
| CYP2A6*5 | CYP2A6 | coumarin | Blood clots, thrombosis, stroke | 10544257 |
| CYP2A6*4B | CYP2A6 | coumarin | Blood clots, thrombosis, stroke | 14583682 |
| CYP2A6*10 | CYP2A6 | coumarin | Blood clots, thrombosis, stroke | 11779172 |
| CYP2A6*6 | CYP2A6 | coumarin | Blood clots, thrombosis, stroke | 11278503 |
| G1202R, S1206Y | ALK | crizotinib | Cancer | 25588040; 25945060;22162641;24433361;25945060 |
| W1282X | CFTR | curcumin | Inflammation | 27007499 |
| rs4673 | CYBA | cyclophosphamide | Cancer | 19448608;25823784 |
| rs1143684 | NQO2 | cyclophosphamide | Cancer | 21946896;29938344 |
| rs1143684 | NQO2 | cyclophosphamide | Cancer | 21946896;29938344 |
| rs12210538 | SLC22A16 | cyclophosphamide | Cancer | 20179710;28036387 |
| rs4673 | CYBA | cyclophosphamide | Cancer | 19448608;25823784 |
| rs4802101 | CYP2B6 | cyclophosphamide | Cancer | 26456622 |
| rs639174 | DROSHA | cyclophosphamide | Cancer | 24614921 |
| rs3213619, rs9501929 | ABCB1, TUBB2A | cyclophosphamide | Cancer | 24599932 |
| rs6907567 | SLC22A16 | cyclophosphamide | Cancer | 20179710 |
| CYP2C9 poor metabolizer | CYP2C9 | cyclophosphamide | Cancer | 26894931 |
| rs2889517 | ABCC1 | cyclophosphamide | Cancer | 23999597 |
| s13058338 | RAC2 | cyclophosphamide | Cancer | 25823784 |
| rs246221 | ABCC1 | cyclophosphamide | Cancer | 26222310 |
| rs2916733 | MCPH1 | cyclophosphamide | Cancer | 25008867 |
| rs9908694 | IKZF3 | cyclophosphamide | Cancer | 24549002 |
| rs1883112 | NCF4 | cyclophosphamide | Cancer | 19448608 |
| CYP3A4*18B | CYP3A4 | cyclosporine | transplant rejection | 23557867;24691060;25976223 |
| rs17514110 | ATIC | cyclosporine | transplant rejection | 25425682 |
| rs2257401 | CYP3A7 | cyclosporine | transplant rejection | 28952408 |
| NOD2 | NOD2 | cyclosporine | transplant rejection | 26107754 |
| rs1800872 | IL10 | cyclosporine | transplant rejection | 18444945 |
| rs28371759 | CYP3A4 | cyclosporine | transplant rejection | 23557867 |
| CYP3A4*1A | CYP3A4 | cyclosporine | transplant rejection | 29043387 |
| rs2167270 | LEP | cyclosporine | transplant rejection | 26282401 |
| rs4643786 | DCK | cytarabine | cancer | 15564883;21521023;27548009 |
| rs2897047 | - | cytarabine | cancer | 28570300 |
| rs11191612 | - | cytarabine | cancer | 21712425 |
| rs9993633 | DCK | cytarabine | cancer | 21521023 |
| rs3750117 | NT5C3A | cytarabine | cancer | 25000516 |
| rs2306744 | DCK | cytarabine | cancer | 15564883 |
| rs1163075 | - | cytarabine | cancer | 21712425 |
| rs3734703 | SLC29A1 | cytarabine | cancer | 27422302 |
| rs4643786 | DCK | cytarabine | cancer | 27548009 |
| rs2244613 | CES1 | Dabigatran | Blood clots, thrombosis, stroke | 23467860;27261537 |
| rs8192935 | CES1 | Dabigatran | Blood clots, thrombosis, stroke | 27261537 |
| rs113488022 | BRAF | dabrafenib | Cancer | 23844038 |
| CYP1A2*11 | CYP1A2 | dacarbazine | Blood glucose lowering agent | 27428168 |
| rs1805087 | MTR | dactinomycin | Infection | 19159907;21605004 |
| A2756G | MTR | dactinomycin | Infection | 19159907;21605004 |
| rs1967309 | ADCY9 | dalcetrapib | High Cholesterol levels | 25583994;27418594 |
| rs1967309 | ADCY9 | dalcetrapib | High Cholesterol levels | 25583994;27418594 |
| rs117714106 | - | darapladib | atherosclerosis | 28753643 |
| rs4294800 | SLCO3A1 | darunavir | Viral infection | 23494984 |
| rs121913459 | ABL1 | dasatinib | Cancer | 22587422;25132497 |
| A142T | AKR1C4 | daunorubicin | Cancer | 20837989 |
| rs4982753, rs4149178 | SLC22A17, SLC22A7 | daunorubicin | Cancer | 26230641 |
| rs6603859 | SZRD1 | daunorubicin | Cancer | 18451141 |
| rs41557318 | CBR1 | daunorubicin | Cancer | 19204081 |
| rs2835285 | CBR3 | daunorubicin | Cancer | 20007405 |
| rs120525235 and rs3750518 | NA | daunorubicin | Cancer | 18451141 |
| rs25678 | CBR1 | daunorubicin | Cancer | 27738808 |
| 8620 AG/GG | CYP24A1 | deferasirox | high iron level | 29099735;29160302 |
| rs2248359 | CYP24A1 | deferasirox | high iron level | 29099735;29160302 |
| rs10929302 | UGT1A6 | deferasirox | high iron level | 26403473;29737521 |
| rs1983023 | UGT1A6 | deferasirox | high iron level | 27043265 |
| rs927650 | CYP24A1 | deferasirox | high iron level | 29160302 |
| rs887929 | DPP6 | deferasirox | high iron level | 27193993 |
| rs2228570 | VDR | deferasirox | high iron level | 29099735 |
| rs2070959 | UGT1A6 | deferiprone | high iron level | 24036429 |
| rs118192175 | RYR1 | desflurane | Anesthetic | 10484775;12124989;9497245 |
| rs118192122 | RYR1 | desflurane | Anesthetic | 10051009;10484775;11575529;12059893;12709367;15448513;16163667;16917943;17081152;19191333;19648156;20681998;21455645;23558838;24433488;25960145;26951757;27382027 |
| rs193922807 | RYR1 | desflurane | Anesthetic | 14641996;24433488 |
| rs118192178 | RYR1 | desflurane | Anesthetic | 16621918;16732084;16917943;19685112;26381711;27918309 |
| rs193922818 | RYR1 | desflurane | Anesthetic | 16621918;16732084;16835904;16917943;17483490;21282829;25960145;26381711 |
| rs118192124 | RYR1 | desflurane | Anesthetic | 10823104;12059893;14985404;22030266;24433488;25086907 |
| rs121918592 | RYR1 | desflurane | Anesthetic | 10051009;12059893;12124989;12151923;12411788;15731587;16163667;16732084;16917943;17710899;18564801;19648156;20681998;21455645;23558838;24433488;25268394;25960145;29101530;8012359;8825043;8828983;8902717;9106529;9334205;9497245;9543323 |
| rs121918596 | RYR1 | desflurane | Anesthetic | 11389482;27857962 |
| rs121918595 | RYR1 | desflurane | Anesthetic | 10888602;16163667;16917943;18564801;19648156;25960145 |
| rs118192176 | RYR1 | desflurane | Anesthetic | 10484775;11575529;11668625;12059893;12411786;12709367;15448513;16163667;16244001;16917943;17710899;19191333;19648156;20681998;21455645;23558838;24433488;25466363;25735680;25989378;9497245 |
| rs1800559 | CACNA1S | desflurane | Anesthetic | 11260227;12411788;15201141;16163667;9199552 |
| rs28933396 | RYR1 | desflurane | Anesthetic | 12059893;12124989;15108991;16163667;16732084;16835904;16917943;17081152;17710899;19513315;19648156;20681998;21965348;25256590;25989378;8220422 |
| rs1801086 | RYR1 | desflurane | Anesthetic | 11575529;12124989;1354642;15448513;16917943;18564801;19346234;19648156;21804810;23558838;25989378 |
| rs193922753 | RYR1 | desflurane | Anesthetic | 16163667;16917943;25658027 |
| rs193922802 | RYR1 | desflurane | Anesthetic | 11525881;15448513;15731587;16163667;16917943;19648156;24433488;25960145;26951757 |
| rs28933397 | RYR1 | desflurane | Anesthetic | 10051009;11668625;12208234;15731587;16163667;16835904;20681998;24433488;9450902;9497245 |
| rs121918593 | RYR1 | desflurane | Anesthetic | 10484775;10700782;10793526;11575529;11668625;12059893;12124989;12151923;12208234;12411786;15448513;15731587;16163667;16732128;16835904;16917943;17081152;18502356;18564801;18945287;19648156;20681998;21455645;21965348;23558838;23628358;24433488;25268394;25735680;25960145;26994242;27382027;27646467;28063098;28326467 |
| rs193922816 | RYR1 | desflurane | Anesthetic | 10484775;10612851;12411788;16163667;20681998;24433488;25611019;25989378;29608462 |
| rs112563513 | RYR1 | desflurane | Anesthetic | 16917943;19191329;19648156;21455645;23558838;23736090;24433488;28403410 |
| rs193922747 | RYR1 | desflurane | Anesthetic | 16163667;17710899;20681998;9066328;9334205;9497245 |
| rs63749869 | RYR1 | desflurane | Anesthetic | 14985404;17081152;17226826;23558838;25989378 |
| rs118192161 | RYR1 | desflurane | Anesthetic | 11575529;12059893;12124989;12151923;12208234;12411788;15448513;15731587;16163667;16732084;16835904;16917943;17122579;18564801;19648156;21455645;21965348;23159934;23558838;25989378;7547049;7889656;8220423;8592342;9334205;9497245 |
| rs118192170 | RYR1 | desflurane | Anesthetic | 10097181;17081152;25989378 |
| rs193922770 | RYR1 | desflurane | Anesthetic | 16244001;16917943;25658027;25989378;9138151;9334205;9497245 |
| rs121918594 | RYR1 | desflurane | Anesthetic | 14985404;16732084;16917943;18564801;19648156;22415532;9450902 |
| rs772226819 | CACNA1S | desflurane | Anesthetic | 19825159;22547813 |
| rs193922772 | RYR1 | desflurane | Anesthetic | 10051009;10484775;16163667;17710899;19346234;20681998;24433488;25989378;9334205;9389851;9497245 |
| rs118192177 | RYR1 | desflurane | Anesthetic | 10484775;11575529;12059893;12208234;12220451;15448513;15731587;16163667;16244001;16835904;16917943;17081152;18564801;19648156;19919814;23558838;24433488;25268394;25735680;25960145;25989378;9497245 |
| rs193922878 | RYR1 | desflurane | Anesthetic | 11928716;16732084;16917943;18306019;19191329 |
| p.Arg174Trp | CACNA1S | desflurane | Anesthetic | 11260227;12411788;15201141;16163667;19825159;22547813;9199552 |
| rs118192163 | RYR1 | desflurane | Anesthetic | 12124989;12208234;12411786;15731587;16732084;16835904;16917943;19648156;21455645;23558838;26951757;9497245 |
| rs193922803 | RYR1 | desflurane | Anesthetic | 12123492;15210166;19648156;23558838;24361844;25256590;28403410 |
| rs118192162 | RYR1 | desflurane | Anesthetic | 16163667;16284304;19020143;7829078 |
| rs193922809 | RYR1 | desflurane | Anesthetic | 12059893;16163667 |
| rs111888148 | RYR1 | desflurane | Anesthetic | 16917943;19191329;19191333 |
| rs118192116 | RYR1 | desflurane | Anesthetic | 8220423 |
| rs193922876 | RYR1 | desflurane | Anesthetic | 18212565 |
| rs61888800 | BDNF | desipramine | Psychiatric Disorders, Major Depression | 19414708 |
| rs885479, rs2228479, rs2228478 | MC1R | desipramine | Psychiatric Disorders, Major Depression | 21052032 |
| rs2298805 | FCER1A | desloratadine | Inflammation, urticaria | 25412950 |
| rs2306862 | LRP5 | dexamethasone | Inflammation | 18285546 |
| rs735482 | ERCC1 | dexamethasone | Inflammation | 19504558 |
| rs6254 | PTH | dexamethasone | Inflammation | 18285546 |
| rs2243057 | F2RL1 | dexamethasone | Inflammation | 28628558 |
| rs9922316 | PRKCB | dexmedetomidine | Pain symptom | 23337848 |
| rs2484516 | ADRA2A | dexmedetomidine | Pain symptom | 23873118 |
| CYP2D6*100 | CYP2D6 | dextromethorphan | Common cough | 18797858;20473659;23394389;8554938 |
| CYP2D6*56 | CYP2D6 | dextromethorphan | Common cough | 12152006;12811367;16415111;16679388;17392730 |
| CYP2D6*44 | CYP2D6 | dextromethorphan | Common cough | 10471060;15618746;18784265;8946471 |
| CYP2D6*42 | CYP2D6 | dextromethorphan | Common cough | 12152006;12811367;16415111;17392730 |
| CYP2D6*101 | CYP2D6 | dextromethorphan | Common cough | 18797858;20473659;23394389;8554938 |
| CYP2D6*15 | CYP2D6 | dextromethorphan | Common cough | 11741249;8807669 |
| CYP2D6*13 | CYP2D6 | dextromethorphan | Common cough | 18797858;20473659;23394389;8554938 |
| CYP2D6*69 | CYP2D6 | dextromethorphan | Common cough | 18797858;20473659;23394389;8554938 |
| rs74966855 | CYP2D6 | dextromethorphan | Common cough | 25141893 |
| CYP2D6*84 | CYP2D6 | dextromethorphan | Common cough | 28290770 |
| CYP2D6*10x2 | CYP2D6 | dextromethorphan | Common cough | 15149890 |
| CYP2D6*59 | CYP2D6 | dextromethorphan | Common cough | 26335396 |
| rs1046428 | GSTZ1 | dichloroacetic acid | familial hyperlipidaemia | 21642471 |
| rs17036170 | PPARG | diclofenac | Pain symptom | 22968431 |
| CYP2C9*59 | CYP2C9 | diclofenac | Pain symptom | 25994031 |
| rs4655226 | CDA | difluorodeoxyuridine | cancer | 22838949 |
| rs10494366 | NOS1AP | Dihydropyridine derivatives | Hypertension | 19076153 |
| rs7756992 | CDKAL1 | Dipeptidyl peptidase 4 (DPP-4) inhibitors | Diabetes | 27139004 |
| rs4149601 | NEDD4L | diuretics | Hypertension | 18591455;21052022;23353631 |
| G460W | ADD1 | diuretics | Hypertension | 11926892;17189961 |
| rs230119 | GNB3 | diuretics | Hypertension | 16314886;17189961 |
| rs4149601 | NEDD4L | diuretics | Hypertension | 18591455;21052022;23353631 |
| I/D polymorphism | ACE | diuretics | Hypertension | 16314886;17189961 |
| rs1801252 | ADRB1 | diuretics | Hypertension | 22543981 |
| D85N | KCNE1 | diuretics | Hypertension | 22100668 |
| rs16960228 | PRKCA | diuretics | Hypertension | 23753411 |
| rs1042714 | ADRB2 | diuretics | Hypertension | 22543981 |
| rs10800397 | NOS1AP | diuretics | Hypertension | 22682551 |
| rs702553 | PDE4D | diuretics | Hypertension | 23778321 |
| rs2301339 | GNB3 | diuretics | Hypertension | 17189961 |
| rs62205366 | GNAS | dobutamine | Hypertension, cardiovascular diseases | 19542315 |
| rs12762549 | - | docetaxel | cancer | 18294295;23188068 |
| rs11045585 | SLCO1B3 | docetaxel | cancer | 18294295;21995462;23188068 |
| rs12960 | SPG7 | docetaxel | cancer | 20038957 |
| rs1382368 | XRCC4 | docetaxel | cancer | 20157331 |
| rs1937840 | AKR1C3 | docetaxel | cancer | 23116553 |
| rs11185648 | RXRA | docetaxel | cancer | 24193570 |
| rs1005230 | VEGFA | docetaxel | cancer | 24061601 |
| rs875858 | VAC14 | docetaxel | cancer | 27143689 |
| rs3212935 | ERCC1 | docetaxel | cancer | 27574448 |
| rs875858 | VAC14 | docetaxel | cancer | 27143689 |
| rs4149118 | SLCO1B3 | docetaxel | cancer | 21995462 |
| rs767531 | C18orf21 | dofetilide | Cardiovascular disease | 24223155 |
| UGT1A1*36 | UGT1A1 | dolutegravir | Viral infection | 24329186 |
| rs2571598 | ACHE | donepezil | Dementia, Alzheimer' Disease | 18780301;25730470 |
| rs2177369 | CHAT | donepezil | Dementia, Alzheimer' Disease | 18780301;25730470 |
| rs2177370 | CHAT | donepezil | Dementia, Alzheimer' Disease | 25730470 |
| rs2177369 | CHAT | donepezil | Dementia, Alzheimer' Disease | 18780301 |
| rs6720975 | PRKCE | donepezil | Dementia, Alzheimer' Disease | 23374588 |
| rs1355534 | BCHE | donepezil | Dementia, Alzheimer' Disease | 18780301 |
| CYP2D6*35xN | CYP2D6 | doxepin | Psychiatric Disorders | 16007002 |
| rs1056892 | CBR3 | doxorubicin | Cancer | 21048526 |
| rs10865801 | AC092422.1 | doxorubicin | Cancer | 26799497 |
| rs1533682 | ABCC5 | doxorubicin | Cancer | 26975227 |
| rs45511401 | ABCC1 | doxorubicin | Cancer | 16330681 |
| rs8133052 | CBR3 | doxorubicin | Cancer | 18551042 |
| rs1058808 | ERBB2 | doxorubicin | Cancer | 28763429 |
| c.146A>G and c.1226T>C | SLC22A16 | doxorubicinol | Cancer | 17559346;20179710 |
| rs714368 | SLC22A16 | doxorubicinol | Cancer | 17559346;20179710 |
| rs20572 | CBR1 | doxorubicinol | Cancer | 19016765 |
| rs7725278 | - | drotrecogin alfa | Blood clots, thrombosis, stroke | 22310353 |
| R506Q | F5 | drotrecogin alfa | Blood clots, thrombosis, stroke | 15118525 |
| rs61908407 | ANO2 | duloxetine | Psychiatric Disorders | 29407288 |
| CYP2D6 extensive metabolizers | CYP2D6 | duloxetine | Psychiatric Disorders | 23545896 |
| rs6313 | HTR2A | duloxetine | Psychiatric Disorders | 24394202 |
| rs324026 | DRD3 | duloxetine | Psychiatric Disorders | 22249355 |
| rs2066992 | IL6 | duloxetine | Psychiatric Disorders | 26556688 |
| rs963468 | DRD3 | duloxetine | Psychiatric Disorders | 22249355 |
| p.Arg885His, p.Arg885Cys | C5 | eculizumab | Cancer | 24521109 |
| rs2279345 | CYP2B6 | efavirenz | Viral infection | 22471906;24293076;24477223;24492364 |
| rs28399454 | CYP2A6 | efavirenz | Viral infection | 19371316;19779319;20860463;22354160;23172109;24316028;26774523;26779253 |
| CYP2B6*11A | CYP2B6 | efavirenz | Viral infection | 19916993;23734829 |
| rs707265 | CYP2B6 | efavirenz | Viral infection | 26779253;28960269 |
| rs8192726 | CYP2A6 | efavirenz | Viral infection | 19371316;19779319;20860463;23172109;23859571;26774523 |
| rs3003596 | NR1I3 | efavirenz | Viral infection | 23173844;23734829 |
| rs35303484 | CYP2B6 | efavirenz | Viral infection | 20860463;25303294;26779253;28886044 |
| rs8192719 | CYP2B6 | efavirenz | Viral infection | 22471906 |
| NAT2 poor metabolizer genotype | NAT2 | efavirenz | Viral infection | 25722197 |
| rs1054190 | GSK3B | efavirenz | Viral infection | 26779253 |
| CYP2B6*27 | CYP2B6 | efavirenz | Viral infection | 17235330 |
| rs7246465 | CYP2B6 | efavirenz | Viral infection | 28960269 |
| rs7438284 | UGT2B7 | efavirenz | Viral infection | 20860463 |
| rs36118214 | CYP2B6 | efavirenz | Viral infection | 19659438 |
| CYP2B6*38 | CYP2B6 | efavirenz | Viral infection | 23640958 |
| rs374527 | RP1 | efavirenz | Viral infection | 25461247 |
| rs8100458 | CYP2B6 | efavirenz | Viral infection | 22471906 |
| CYP2B6*16 | CYP2B6 | efavirenz | Viral infection | 16495778 |
| CYP2B6*26 | CYP2B6 | efavirenz | Viral infection | 17918089 |
| rs73420732 | EGFR | egfr inhibitors | Cancer | 26419366 |
| HLA-A*02:01 | HLA-A | egfr inhibitors | Cancer | 23057547 |
| rs11646054 | SLC5A2 | empagliflozin | Blood glucose lowering agent, diuretics | 28134748 |
| rs8176746 | ABO | enalapril | Hypertension | 24743543 |
| rs699947, rs1570360 | VEGFA | enalapril | Hypertension | 26002049 |
| rs2070950 | NR3C2 | enalapril | Hypertension | 24059494 |
| rs61722009 | NOS3 | enalapril | Hypertension | 22706620 |
| rs2010963 | VEGFA | enalapril | Hypertension | 26002049 |
| CYP2D6*35 | CYP2D6 | endoxifen | Psychiatric Disorders | 16815318 |
| SULT1A2*1 | SULT1A2*1 | endoxifen | Psychiatric Disorders | 23922954 |
| rs5758550 | WBP2NL | endoxifen | Psychiatric Disorders | 28745555 |
| CYP2D6*8 | CYP2D6 | endoxifen | Psychiatric Disorders | 14652237 |
| CYP2D6 poor metabolizers and intermediate metabolizers | CYP2D6 | endoxifen | Psychiatric Disorders | 21768473 |
| extensive metabolizer and ultra-metabolizer | extensive metabolizer and ultra-metabolizer | endoxifen | Psychiatric Disorders | 27226358 |
| rs4386686 | LINC02057 | endoxifen | Psychiatric Disorders | 24699530 |
| rs3832043 | UGT1A10 | entacapone | Neurodegenerative disease, Parkinson's Disease | 22527346 |
| rs1042719 | ADRB2 | ephedrine | Hypertension, cardiovascular diseases | 26771271 |
| rs1799945 | HFE | epoetin alfa | Anemia | 18025780 |
| CYP2C19*4 | CYP2C19 | escitalopram | Psychiatric Disorders, Major Depression | 11802100;12968986;12975335;15168101;16418702;16855453;17625515;18382661;18654768;19404631;19593158;19773541;19840783;19841156;20350136;20531370;21192344;21926427;23545896;23981149;24014145;24257813;24858822;25122046;25154506;8451774;8835706 |
| rs12248560 | CYP2C19 | escitalopram | Psychiatric Disorders, Major Depression | 16413245;17625515;20531370 |
| SLC6A4 HTTLPR short form (S allele) | SLC6A4 | escitalopram | Psychiatric Disorders, Major Depression | 19272758;19375170;19567893;20212518;21388237;23095326;24014145 |
| SLC6A4 HTTLPR long form (L allele) | SLC6A4 | escitalopram | Psychiatric Disorders, Major Depression | 19272758;19375170;19567893;20212518;21388237;23095326;24014145 |
| CYP2C19 poor metabolizer | CYP2C19 | escitalopram | Psychiatric Disorders, Major Depression | 24257813;24858822 |
| rs1065852 | CYP2D6 | escitalopram | Psychiatric Disorders, Major Depression | 24302953;24528284 |
| rs25531 | SLC6A4 | escitalopram | Psychiatric Disorders, Major Depression | 19272758;19375170;19567893;20212518;21388237;23095326;24014145 |
| CYP2C19 poor metabolizers | CYP2C19 | escitalopram | Psychiatric Disorders, Major Depression | 19404631;24014145;24257813;25122046;25761838;27524899 |
| rs2069526 | CYP1A2 | escitalopram | Psychiatric Disorders, Major Depression | 23859573 |
| rs4986893 | CYP2C19 | escitalopram | Psychiatric Disorders, Major Depression | 17625515 |
| rs3892097 | CYP2D6 | escitalopram | Psychiatric Disorders, Major Depression | 18070221 |
| rs915120 | GRK5 | escitalopram | Psychiatric Disorders, Major Depression | 22907730 |
| rs11580409 | ERICH3 | escitalopram | Psychiatric Disorders, Major Depression | 26903268 |
| C825T | GNB3 | escitalopram | Psychiatric Disorders, Major Depression | 20826553 |
| rs10975641 | GLDC | escitalopram | Psychiatric Disorders, Major Depression | 21107318 |
| CYP2D6 intermediate metabolizers | CYP2D6 | escitalopram | Psychiatric Disorders, Major Depression | 21926427 |
| rs11568817 | HTR1B | escitalopram | Psychiatric Disorders, Major Depression | 22717018 |
| rs1126757 | IL11 | escitalopram | Psychiatric Disorders, Major Depression | 23142150 |
| rs12054895 | - | escitalopram | Psychiatric Disorders, Major Depression | 23377640 |
| CYP2C19 heterozygous extensive metabolizer | CYP2C19 | escitalopram | Psychiatric Disorders, Major Depression | 24858822 |
| rs6318 | HTR2C | escitalopram | Psychiatric Disorders, Major Depression | 21614492 |
| rs962369 | BDNF | escitalopram | Psychiatric Disorders, Major Depression | 19641488 |
| rs9380524 | FKBP5 | escitalopram | Psychiatric Disorders, Major Depression | 23324805 |
| rs57098334 | SLC6A4 | escitalopram | Psychiatric Disorders, Major Depression | 24014145 |
| rs1074145 | - | escitalopram | Psychiatric Disorders, Major Depression | 24528284 |
| rs7997012 | HTR2A | escitalopram | Psychiatric Disorders, Major Depression | 26262902 |
| CYP2C19 ultrarapid metabolizer phenotype | CYP2C19 | escitalopram | Psychiatric Disorders, Major Depression | 24257813 |
| rs41271330 | BMP5 | escitalopram | Psychiatric Disorders, Major Depression | 23926243 |
| rs5443 | GNB3 | escitalopram | Psychiatric Disorders, Major Depression | 20826553 |
| rs2032583 | ABCB1 | escitalopram | Psychiatric Disorders, Major Depression | 26704739 |
| CYP2C19*1A | CYP2C19 | esomeprazole | Stomach ulcers | 19166419 |
| CYP3A5, CYP2C19, CYP2C9, CYP2D6 | CYP3A5 | esomeprazole | Stomach ulcers | 18695978 |
| rs2369049 | TCL1A | estradiol | Menopause symptoms | 22405131 |
| rs1864729 | TSPYL5 | estradiol | Menopause symptoms | 23518928 |
| rs1800630 | LTA | etanercept | Rheumatoid arthritis | 15695296 |
| rs13437088 | - | etanercept | Rheumatoid arthritis | 28470127 |
| rs28362491 | NFKB1 | etanercept | Rheumatoid arthritis | 27348478 |
| rs2070672 | CYP2E1 | ethambutol | Tuberculosis | 19891553;20941486 |
| NAT2*7A | NAT2 | ethambutol | Tuberculosis | 20392357;21753138 |
| rs1799930 | NAT2 | ethambutol | Tuberculosis | 18421452;19891553;20941486;21856096;22506592;29036176 |
| rs9332096 | CYP2C9 | ethambutol | Tuberculosis | 19891553;20941486 |
| rs4646244 | NAT2 | ethambutol | Tuberculosis | 19891553;20941486 |
| rs1041983 | NAT2 | ethambutol | Tuberculosis | 18421452;22506592;29036176 |
| rs1799931 | NAT2 | ethambutol | Tuberculosis | 11595069;19686464;19891553;20392357;20941486;22506592;29036176 |
| rs4646267 | NAT2 | ethambutol | Tuberculosis | 19891553;20941486 |
| rs2008584 | UGT1A6 | ethambutol | Tuberculosis | 19891553 |
| STAT3 TCG | STAT3 TCG | ethambutol | Tuberculosis | 25789467 |
| NAT2*5D | NAT2 | ethambutol | Tuberculosis | 20392357 |
| rs4149032 | SLCO1B1 | ethambutol | Tuberculosis | 21709081 |
| rs283411 | ADH1C | ethanol | Psychiatric disorders | 19193628 |
| rs13280604 | CHRNB3 | ethanol | Psychiatric disorders | 18055561 |
| rs2975226 | SLC6A3 | ethanol | Psychiatric disorders | 28182634 |
| K172N | TAS2R16 | ethanol | Psychiatric disorders | 16385453 |
| rs1229984 | ADH1B | ethanol | Psychiatric disorders | 23240771 |
| rs11575893 | CARTPT | ethanol | Psychiatric disorders | 20362026 |
| rs1229967 | ADH1A | ethanol | Psychiatric disorders | 19193628 |
| rs2232165 | GHSR | ethanol | Psychiatric disorders | 18828808 |
| rs1176744 | HTR3B | ethanol | Psychiatric disorders | 20838391 |
| rs11940694 | KLB | ethanol | Psychiatric disorders | 27911795 |
| ALDH2*2 | ALDH2 | ethanol | Psychiatric disorders | 25365528 |
| rs1799972 | OPRM1 | ethanol | Psychiatric disorders | 21070507 |
| rs446112 | ZNF663P | etoposide | Cancer | 17537913 |
| rs10515074 | PIK3R1 | everolimus | Cancer | 28727815 |
| MTOR | MTOR | everolimus | Cancer | 25893859 |
| CYP1A1*2A | CYP1A1 | exemestane | Osteoporosis | 27549341 |
| rs11253043 | AKR1C4 | exemestane | Osteoporosis | 27111237 |
| rs934635 | MIR4713HG | exemestane | Osteoporosis | 24590773 |
| rs4870061 | ESR1 | exemestane | Osteoporosis | 26536870 |
| rs9322336 | ESR1 | exemestane | Osteoporosis | 23546553 |
| rs1884725, rs13415401 | XDH | febuxostat | Rheumatoid arthritis | 24915143;27798726 |
| L162V, P207L | LPL | fenofibrate | High Cholesterol levels | 12042669;19207029 |
| rs3135506 | ZPR1 | fenofibrate | High Cholesterol levels | 17431185;19056598;19057464 |
| rs1800206 | PPARA | fenofibrate | High Cholesterol levels | 12042669;15608561 |
| rs2230808 | ABCA1 | fenofibrate | High Cholesterol levels | 20346718 |
| rs2727786 | APOC3 | fenofibrate | High Cholesterol levels | 19207029 |
| rs3091244 | CRP | fenofibrate | High Cholesterol levels | 18285551 |
| rs135550 | PPARA | fenofibrate | High Cholesterol levels | 22547144 |
| rs11216158 | APOA1 | fenofibrate | High Cholesterol levels | 19057464 |
| rs675 | APOA4 | fenofibrate | High Cholesterol levels | 19057464 |
| rs9626730 | PPARA | fenofibrate | High Cholesterol levels | 22547144 |
| rs2241883 | FABP1 | fenofibrate | High Cholesterol levels | 15249972 |
| rs320 | LPL | fenofibrate | High Cholesterol levels | 19207029 |
| rs1801177 | LPL | fenofibrate | High Cholesterol levels | 12042669 |
| rs964184 | ZPR1 | fenofibrate | High Cholesterol levels | 23119086 |
| rs676210 | APOB | fenofibrate | High Cholesterol levels | 20724655 |
| SLC22A1*4 | SLC22A1 | fenoterol | Asthma | 28791698 |
| rs12211463 | - | fentanyl | Pain symptom | 21622719 |
| rs9384179 | OPRM1 | fentanyl | Pain symptom | 25615449 |
| rs2076222 | CAMK1G | fentanyl | Pain symptom | 26566055 |
| rs2473967 | - | fentanyl | Pain symptom | 21622719 |
| rs6853 | MYD88 | fentanyl | Pain symptom | 26332828 |
| rs2306168 | SLCO2B1 | fexofenadine | Allergy | 21280267 |
| rs80338792 | SCN4A | flecainide | Cardiovascular disease | 23052413 |
| rs10937275 | ST6GAL1 | flucloxacillin | Infection | 19483685 |
| HLA-B*57:01:01 | HLA-B | flucloxacillin | Infection | 19483685 |
| rs1131873 | EPHX1 | fluindione | Thrombosis | 21883387 |
| rs2292566 | EPHX1 | fluindione | Thrombosis | 21883387 |
| rs9923231 | VKORC1 | fluindione | Thrombosis | 22130800 |
| rs2305089 | TBXT | flunisolide | Inflammation | 22538805 |
| rs1801131 | C1orf167 | fluorouracil | cancer | 17700593;18245544;20078613;20385995;20714149;20819423;22045187;23407049;23736036;23775025;24167597;25331073;26967565;27557140;27738344;27864592 |
| rs1799983 | NOS3 | fluorouracil | cancer | 19671875;25545243;29938344 |
| 295-298delTCAT, 1897delC | DPYD | fluorouracil | cancer | 10027340;10071185;10234617;10803677;11156223;11350878;11555601;11895907;11953843;11988088;12209976;12360106;14635116;15017333;15132136;15591715;15858133;15944764;16115930;16151913;16361556;17000685;17064846;17121937;17165084;17203168;17335544;17417073;17700593;17848752;18299612;18443386;18452418;18537153;18600527;19104657;19473056;19530960;19795123;19822137;19858398;20204365;20385995;20507294;20530282;20601926;20665215;20714149;20803296;20809970;20819423;21077799;21410976;21498394;22310351;22490566;23328581;23481061;23585145;23588312;23588952;23603345;23736036;23930673;23942539;23988873;24037119;24107927;24167597;24388031;24434920;24590654;24647007;24648345;24700034;24817302;24923815;25381393;25410891;25677447;25782327;25796495;26099996;26216193;26265035;26265346;26330892;26392323;26603945;26658227;26792652;26794347;26804652;26846104;26967565;27454530;27544765;27738344;27752409;27864592;27995989;28278081;28295243;28395758;28427087;28481884;28614820;28929491;29065426;29134491;29769267;3335642;7964939;8698850;9266349;9323575;9439663;9470816;9472650;9865912 |
| rs3918290 | DPYD | fluorouracil | cancer | 10071185;10803677;11156223;11350878;11555601;11895907;11953843;11988088;12209976;12360106;15017333;15858133;16151913;16361556;17000685;17064846;17121937;17165084;17203168;17335544;17700593;18299612;18443386;18600527;19104657;19473056;19530960;19795123;19858398;20385995;20507294;20530282;20803296;20809970;20819423;21410976;21498394;23328581;23481061;23585145;23603345;23736036;23930673;24167597;24590654;24647007;24700034;24817302;24923815;25381393;25410891;25677447;26099996;26216193;26265035;26603945;26792652;26794347;26804652;26846104;26967565;27454530;27738344;27864592;28295243;28481884;28929491;29065426;29134491;8698850;9323575;9439663;9470816 |
| rs45589337 | DPYD | fluorouracil | cancer | 14635116;18299612;24648345;24923815 |
| rs2010963 | VEGFA | fluorouracil | cancer | 24090479;25955730 |
| DPYD deficiency | DPYD | fluorouracil | cancer | 10027340;11156223;12209976;20204365;3335642;7964939 |
| rs56038477 | DPYD | fluorouracil | cancer | 16115930;18299612;19473056;19530960;20803296;21498394;23588312;24167597;24590654;24647007;24923815;26265346;26603945;27995989;28481884 |
| rs1042522 | TP53 | fluorouracil | cancer | 18357466;19052714;19786980;20638924;22188361;26696550 |
| rs1801160 | DPYD | fluorouracil | cancer | 10803677;11156223;16115930;18299612;19473056;19530960;21498394;22490566;23328581;23588312;24590654;24647007;24923815;26099996;26216193;26265035;26330892;26794347;26804652;26846104;28481884;28614820;29065426;29769267 |
| rs699947 | VEGFA | fluorouracil | cancer | 20125120;25955730 |
| DPYD*9B | DPYD | fluorouracil | cancer | 10071185;9266349;9439663 |
| rs72549307 | DPYD | fluorouracil | cancer | 24648345;24923815 |
| rs1801019 | UMPS | fluorouracil | cancer | 16818689;20647221;20665215;24167597 |
| rs1801265 | DPYD | fluorouracil | cancer | 10071185;10234617;10803677;11156223;11895907;11988088;14635116;15591715;16115930;16361556;17064846;17121937;17848752;18299612;18452418;18537153;19473056;19530960;20530282;20714149;20819423;21498394;23328581;23588312;24037119;24434920;24590654;24647007;24923815;25410891;25677447;26216193;26265035;26330892;26794347;26804652;26846104;27752409;29065426;9439663 |
| TYMP | TYMP | fluorouracil | cancer | 20601926;24167597 |
| DPYD*1 | DPYD | fluorouracil | cancer | 10071185;11953843;15591715;15858133;19473056;20385995;20530282;20714149;23481061;23942539;24037119;24700034;25381393;25796495;26330892;27752409;29134491;8698850;9266349;9439663;9470816 |
| rs25487 | XRCC1 | fluorouracil | cancer | 16875718;20530282;21057378;21167658;23314736;25232828 |
| rs72549306 | DPYD | fluorouracil | cancer | 24648345;29769267;9865912 |
| rs895819 | ZSWIM4 | fluorouracil | cancer | 24401318;25655103;25782327;26804235 |
| rs1870377 | KDR | fluorouracil | cancer | 24090479;25589620 |
| rs188052243 | DPYD | fluorouracil | cancer | 24648345;29769267 |
| rs148994843 | DPYD | fluorouracil | cancer | 24648345;29769267 |
| DPYD*4 | DPYD | fluorouracil | cancer | 15591715;18299612;19473056;19530960;20803296;23736036;26603945;26792652;9472650 |
| Arg72Pro | TP53 | fluorouracil | cancer | 18357466;19052714;19786980;20638924;22188361;26696550;29593529 |
| DPYD*13 | DPYD | fluorouracil | cancer | 11895907;25796495;26265346;26603945 |
| rs115232898 | DPYD | fluorouracil | cancer | 16361556;23588312;24037119;24107927;24388031;24648345 |
| rs4646 | CYP19A1 | fluorouracil | cancer | 25793413;26191232 |
| rs2669429 | DPYS | fluorouracil | cancer | 23736036;26244261 |
| rs2070474 | UPB1 | fluorouracil | cancer | 23238479;26244261 |
| rs2070474 | UPB1 | fluorouracil | cancer | 23238479;26244261 |
| rs13181 | ERCC2 | fluorouracil | cancer | 18267032;20078613;20385995;21449681;23962907;25232828;28796378 |
| rs67376798 | DPYD | fluorouracil | cancer | 11156223;11988088;16115930;17064846;17121937;17700593;18299612;19104657;19795123;20819423;21077799;21410976;21498394;23603345;23736036;23930673;24167597;24590654;24647007;24648345;24923815;25381393;25410891;25677447;26099996;26216193;26265035;26265346;26603945;26794347;26804652;27454530;27995989;28295243;28427087;28481884;29065426 |
| rs17376848 | DPYD | fluorouracil | cancer | 10803677;14635116;16361556;18299612;18452418;19473056;19530960;20819423;21498394;23942539;24923815;25677447;25782327;26099996;26794347;28481884;29065426 |
| rs2297595 | DPYD | fluorouracil | cancer | 11156223;11895907;14635116;16115930;18299612;19104657;19473056;19530960;20819423;21498394;22490566;23588312;23736036;24590654;24647007;24648345;24923815;25410891;25677447;25782327;26099996;26216193;26265035;26794347;26804652;28481884;29065426 |
| rs72549303 | DPYD | fluorouracil | cancer | 10071185;24648345;9266349;9439663 |
| rs59086055 | DPYD | fluorouracil | cancer | 24648345;29769267 |
| rs1801268 | DPYD | fluorouracil | cancer | 10071185;24648345 |
| Glu298Asp | NOS3 | fluorouracil | cancer | 19671875;25545243;29938344 |
| 2251A>C | ERCC2 | fluorouracil | cancer | 18267032;20078613;20385995;21449681;23962907;25232828;28796378 |
| (-241)C/C | XRCC3 | fluorouracil | cancer | 17549067;22868256 |
| rs147601618 | DPYD | fluorouracil | cancer | 19822137;24648345 |
| rs1059829 | SPARC | fluorouracil | cancer | 22491017;28687963 |
| DPYD*2A | DPYD | fluorouracil | cancer | 11156223;11350878;11895907;11953843;12360106;15858133;18299612;19104657;20385995;20530282;20803296;23481061;23736036;24700034;25381393;26265346;26603945;26792652;29134491;8698850;9470816 |
| rs61622928 | DPYD | fluorouracil | cancer | 23588312;24648345 |
| DPYD*6 | DPYD | fluorouracil | cancer | 11156223;18299612;19530960;26330892 |
| rs2669429 | DPYS | fluorouracil | cancer | 23736036;26244261 |
| rs78060119 | DPYD | fluorouracil | cancer | 24648345;26846104 |
| rs56293913 | DPYD | fluorouracil | cancer | 19104657;19530960;23588312 |
| rs55886062 | DPYD | fluorouracil | cancer | 10803677;11895907;11988088;16361556;17121937;19530960;19795123;21410976;21498394;23328581;23588312;23736036;24647007;24923815;25381393;25410891;25796495;26099996;26265035;26603945;26794347;28295243;28427087 |
| DPYD*5 | DPYD | fluorouracil | cancer | 11156223;15591715;17417073;18299612;19473056;19530960;20530282;20714149;20803296;23942539;24037119 |
| R399Q | XRCC1 | fluorouracil | cancer | 16875718;20530282;21057378;21167658;23314736;25232828 |
| rs1801266 | DPYD | fluorouracil | cancer | 10071185;20819423;24648345;9266349;9439663 |
| ABCC5 | ABCC5 | fluorouracil | cancer | 20601926;26352872 |
| rs75017182 | DPYD | fluorouracil | cancer | 20803296;23736036;24167597;24923815;25410891;25782327;26658227;26794347;27544765;28295243;28427087;28614820;29065426 |
| rs25648 | KDR | fluorouracil | cancer | 24090479;25589620 |
| rs1979277 | SHMT1 | fluorouracil | cancer | 22044939;24167597 |
| rs1801159 | DPYD | fluorouracil | cancer | 10803677;11156223;14635116;15132136;15591715;16115930;16361556;17417073;17848752;18299612;18452418;18537153;19530960;20530282;20665215;20714149;21498394;22490566;23328581;23588312;23588952;23942539;24037119;24590654;24647007;24923815;25410891;25677447;26099996;26216193;26265035;26794347;26804652;28481884;29065426;9472650 |
| rs1801158 | DPYD | fluorouracil | cancer | 10803677;14635116;15591715;16115930;18299612;19530960;21498394;23328581;23588312;23736036;24590654;24647007;24923815;25410891;26099996;26216193;26265035;26603945;26794347;26804652;27995989;28427087;28481884;29065426;9472650 |
| rs3025039 | VEGFA | fluorouracil | cancer | 24090479;25955730 |
| DPYD*9A | DPYD | fluorouracil | cancer | 11156223;15591715;18299612;19530960;20530282;20714149;20803296;20819423;23481061;24037119;26330892;27752409 |
| rs2236722 | CYP19A1 | fluorouracil | cancer | 25372392;25793413;26191232 |
| rs17822471 | ABCC11 | fluorouracil | cancer | 24024896;27001120 |
| rs72549309 | DPYD | fluorouracil | cancer | 10071185;24648345 |
| rs6752303 | GALNT14 | fluorouracil | cancer | 21635146 |
| rs181501757 | TOP2A | fluorouracil | cancer | 29593529 |
| rs1801265 | DPYD | fluorouracil | cancer | 20601926 |
| rs2298881, rs10040363, rs2075685 | ERCC1, XRCC4 | fluorouracil | cancer | 28796378 |
| rs2286455 | PROM1 | fluorouracil | cancer | 22231565 |
| rs4073 | CXCL8 | fluorouracil | cancer | 25589620 |
| rs2465403 | COLEC10 | fluorouracil | cancer | 22310351 |
| CYP3A4*1B | CYP3A4 | fluorouracil | cancer | 20530282 |
| rs2854744 | IGFBP3 | fluorouracil | cancer | 20860465 |
| rs225440 | ABCG1 | fluorouracil | cancer | 26352872 |
| rs7121 | GNAS | fluorouracil | cancer | 19274060 |
| rs17822471 | ABCC11 | fluorouracil | cancer | 27001120 |
| -rs4426527, -rs34116584 and del-74 bp | AGXT | fluorouracil | cancer | 22868256 |
| rs17109924 | LGR5 | fluorouracil | cancer | 25665511 |
| rs72547601 | DPYD | fluorouracil | cancer | 24648345 |
| rs163182 | KCNQ1 | fluorouracil | cancer | 26115082 |
| rs3218592 | REV3L | fluorouracil | cancer | 25372392 |
| rs56005131 | DPYD | fluorouracil | cancer | 29769267 |
| rs1610696 | HLA-G | fluorouracil | cancer | 26633805 |
| rs2959023 | DPYS | fluorouracil | cancer | 26244261 |
| rs56276561 | DPYD | fluorouracil | cancer | 19530960 |
| rs2290344 | PIGB | fluorouracil | cancer | 28537877 |
| TPMT*2 | TPMT | fluorouracil | cancer | 26792652 |
| rs861539 | KLC1 | fluorouracil | cancer | 17549067 |
| rs1051266 | SLC19A1 | fluorouracil | cancer | 24143213 |
| rs25648 | FLT4 | fluorouracil | cancer | 24090479 |
| XPD Lys751Gln-GSTP1 105Val haplotype | GSTP1 | fluorouracil | cancer | 21449681 |
| rs854560 | PON1 | fluorouracil | cancer | 25545243 |
| DPYD poor metabolizer | DPYD | fluorouracil | cancer | 26392323 |
| rs376073289 | DPYD | fluorouracil | cancer | 18299612 |
| rs3917412 | SELE | fluorouracil | cancer | 24980946 |
| rs10065756 | SPARC | fluorouracil | cancer | 28687963 |
| Arg388 | FGFR4 | fluorouracil | cancer | 16822847 |
| rs470119 | TYMP | fluorouracil | cancer | 20665215 |
| rs1056836 | RMDN2 | fluorouracil | cancer | 24958282 |
| rs115632870 | DPYD | fluorouracil | cancer | 23588312 |
| rs7699188 | ABCG2 | fluorouracil | cancer | 24018773 |
| rs1127648 | CSPG4 | fluorouracil | cancer | 25069475 |
| rs9561778 | ABCC4 | fluorouracil | cancer | 19696793 |
| rs11479 | TYMP | fluorouracil | cancer | 24167597 |
| rs1799793 | ERCC2 | fluorouracil | cancer | 23962907 |
| p.L254P | NT5C1A | fluorouracil | cancer | 26906009 |
| rs2811178 | DPYD | fluorouracil | cancer | 19530960 |
| rs1801267 | DPYD | fluorouracil | cancer | 26794347 |
| rs56160474 | DPYD | fluorouracil | cancer | 22490566 |
| rs2853542 | TYMS | fluorouracil | cancer | 27001118 |
| rs2070744 | NOS3 | fluorouracil | cancer | 19671875 |
| rs3130 | PROM1 | fluorouracil | cancer | 22231565 |
| rs3812718 | SCN1A | fluorouracil | cancer | 23752739 |
| rs11671784 | ZSWIM4 | fluorouracil | cancer | 25655103 |
| rs16857540, rs2465403, rs10876844, rs10784749, rs17626122, rs7325568 and rs4243761 | COLEC10 | fluorouracil | cancer | 22310351 |
| rs1059829 | SPARC | fluorouracil | cancer | 22491017 |
| UPP1 | UPP1 | fluorouracil | cancer | 20601926 |
| DPYD*12 | DPYD | fluorouracil | cancer | 9865912 |
| rs142244113 | INSR | fluorouracil | cancer | 29593529 |
| rs3212948 | ERCC1 | fluorouracil | cancer | 20385995 |
| rs2293347 | EGFR | fluorouracil | cancer | 23816762 |
| rs61764370 | LYRM5 | fluorouracil | cancer | 23324806 |
| rs2010851 | WNT5B | fluorouracil | cancer | 23817222 |
| rs183205964 | TYMS | fluorouracil | cancer | 26189437 |
| rs7194667 | ABCC11 | fluorouracil | cancer | 24024896 |
| rs12659 | SLC19A1 | fluorouracil | cancer | 16875718 |
| DPYD*11 | DPYD | fluorouracil | cancer | 9865912 |
| rs10209881 | GALNT14 | fluorouracil | cancer | 21635146 |
| DPYD*2B | DPYD | fluorouracil | cancer | 9472650 |
| rs148013902 | MAST3 | fluorouracil | cancer | 29593529 |
| rs367619008 | DPYD | fluorouracil | cancer | 19473056 |
| rs9361233 | HTR1B | fluoxetine | Psychiatric Disorders | 28025020 |
| rs10008257 | - | fluoxetine | Psychiatric Disorders | 24040476 |
| rs230504 | NFKB1 | flupenthixol | Psychiatric Disorders | 27992301 |
| HLA-DRB1*16:01:01 | HLA-DRB1 | flupirtine | Pain symptom | 26959717 |
| rs1876831 | CRHR1 | fluticasone propionate | Allergy | 22370858 |
| rs2872507 | - | fluticasone propionate | Allergy | 22986918 |
| rs1786929 | CTNNA3 | fluticasone propionate | Allergy | 24407380 |
| rs5882 | CETP | fluvastatin | High Cholesterol levels | 16002074 |
| SLCO1B1*14 | SLCO1B1 | fluvastatin | High Cholesterol levels | 18781850 |
| rs1449683 | FGF2 | fluvoxamine | Psychiatric Disorders | 25710119 |
| rs11942223 | SLC2A9 | furosemide | Hypertension | 28951782 |
| rs17268282 | ABCC4 | furosemide | Hypertension | 26927285 |
| CYP2D6*4xN | CYP2D6 | gefitinib | cancer | 16719544;21502555;23207012;23664723;25554506;26323212;26898617 |
| rs121434569 | EGFR | gefitinib | cancer | 15737014;16258541;16912157;17020982;17085664;18093943;18596266;18981003;18992959;19381876;19589612;20129249;21233402;21248300;21430269;21921847;22215752 |
| CYP2D6*14A | CYP2D6 | gefitinib | cancer | 16719544;21502555;23207012;23664723;25554506;26323212;26898617 |
| CYP3A4*1G | CYP3A4 | gefitinib | cancer | 25554506;26898617 |
| rs121434568 | EGFR | gefitinib | cancer | 15118073;15897572;16115929;16203769;16204011;16865253;16956694;17047654;17106442;17192902;17285735;17317677;17387341;17429313;17473659;19692680;20022809;20038723;20573926;21900837;22215752;22370314;22581822;22760226;26490356 |
| CYP2D6*98 | CYP2D6 | gefitinib | cancer | 28461741 |
| rs12145722 | - | gefitinib | cancer | 21787189 |
| rs2622604 | ABCG2 | gefitinib | cancer | 21332310 |
| CYP2D6*36 | CYP2D6 | gefitinib | cancer | 26898617 |
| rs72552713 | ABCG2 | gefitinib | cancer | 20035425 |
| rs1060896 | SLC28A2 | gemcitabine | cancer | 18538445;20665488;21590444 |
| rs12046844 | - | gemcitabine | cancer | 22293537;24361227 |
| Asn58Asp | DCTD | gemcitabine | cancer | 16551864;18538445;20665488;22838950 |
| rs4694362 | DCK | gemcitabine | cancer | 20665488;24361227 |
| rs2242048 | SLC28A1 | gemcitabine | cancer | 18538445;20665488 |
| rs2242047 | SLC28A1 | gemcitabine | cancer | 18538445;22838950 |
| rs4742 | DCTD | gemcitabine | cancer | 18538445;20665488 |
| rs8187758 | SLC28A1 | gemcitabine | cancer | 18538445;20665488 |
| rs2242046 | SLC28A1 | gemcitabine | cancer | 18538445;21590444 |
| rs11141915 | DAPK1 | gemcitabine | cancer | 22293537;24361227 |
| rs11141915 | DAPK1 | gemcitabine | cancer | 22293537;24361227 |
| rs12648166 | DCK | gemcitabine | cancer | 20665488;24361227 |
| rs11719165 | - | gemcitabine | cancer | 22293537 |
| rs9937 | PDE4B | gemcitabine | cancer | 24361227 |
| rs35932500 | DCTD | gemcitabine | cancer | 16551864 |
| rs1705772 | ALG10 | gemcitabine | cancer | 24225399 |
| rs780668 | SLC29A3 | gemcitabine | cancer | 29751792 |
| rs10883617 | BTRC | gemcitabine | cancer | 22142827 |
| rs760370 | SLC29A2 | gemcitabine | cancer | 25162786 |
| rs7543016 | CMPK1 | gemcitabine | cancer | 22838950 |
| CDA+435 C>T | CDA | gemcitabine | cancer | 18538445 |
| rs4925193 | CDH4 | gemcitabine | cancer | 27749787 |
| rs11853372 | SLC28A1 | gemcitabine | cancer | 24300978 |
| rs17215836 | SLC28A1 | gemcitabine | cancer | 18538445 |
| rs7771466, rs763780 | IL17F | gemcitabine | cancer | 22142827 |
| rs66878317 | DCK | gemcitabine | cancer | 23230131 |
| rs1901440 | - | gemcitabine | cancer | 22293537 |
| rs324148 | SLC29A1 | gemcitabine | cancer | 20665488 |
| rs73748206 | FKBP5 | gemcitabine | cancer | 23936393 |
| rs2070673 | CYP2E1 | gemtuzumab ozogamicin | Cancer | 22584460 |
| rs12459419 | CD33 | gemtuzumab ozogamicin | Cancer | 23444229 |
| rs2515641 | CYP2E1 | gemtuzumab ozogamicin | Cancer | 22584460 |
| UGT1A3*2 | UGT1A3 | gepirone hydrochloride | Psychiatric Disorders | 25192553 |
| rs1012335 | IFNAR1 | glatiramer acetate | Multiple sclerosis | 22111603 |
| rs1051922 | IFNB1 | glatiramer acetate | Multiple sclerosis | 22111603 |
| DRB1*15 + TGFB1*T + CCR5*d + IFNAR1*G and DRB1*15 + TGFB1*T + CCR5*d | NA | glatiramer acetate | Multiple sclerosis | 22111603 |
| rs12459996 | AXL | glatiramer acetate | Multiple sclerosis | 28829817 |
| rs4148776 | ABCB11 | glibenclamide | Cancer | 27403931 |
| rs2237897 | KCNQ1 | gliclazide | Diabetes | 26866747 |
| rs2298826 | SLC6A5 | haloperidol | Psychiatric Disorders | 20859245;27023437 |
| NTS | NTS | haloperidol | Psychiatric Disorders | 27023437 |
| CYP2D6*10A | CYP2D6 | haloperidol | Psychiatric Disorders | 12919180 |
| rs2298826 | SLC6A5 | haloperidol | Psychiatric Disorders | 20859245 |
| rs2412459 | EIF2AK4 | haloperidol | Psychiatric Disorders | 24751813 |
| rs1003641 | ANKK1 | haloperidol | Psychiatric Disorders | 22893251 |
| CYP2D6*1A | CYP2D6 | haloperidol | Psychiatric Disorders | 9352580 |
| rs8450 | CRTC2 | hdl cholesterol | High Cholesterol levels | 26644205 |
| HLA-DRB3*01:01:02:01 | HLA-DRB3 | heparin | Blood clots, thrombosis, stroke | 28688202 |
| rs2237562 | GRM3 | heroin | Pain symptom | 24498053 |
| rs9288993 | DRD3 | heroin | Pain symptom | 25521358 |
| rs5746136 | SOD2 | heroin | Pain symptom | 29459008 |
| rs17244841 | HMGCR | hmg coa reductase inhibitors | High Cholesterol levels | 15199031;18332269 |
| rs10455872 | LPA | hmg coa reductase inhibitors | High Cholesterol levels | 22331829;23903772;25350695;27045730;29703846 |
| rs17238540 | HMGCR | hmg coa reductase inhibitors | High Cholesterol levels | 15199031;16103896;17563401;18261733;18332269;18815589 |
| rs9806699 | C15orf48 | hmg coa reductase inhibitors | High Cholesterol levels | 23995691;25863251;27839692 |
| rs1532624 | CETP | hmg coa reductase inhibitors | High Cholesterol levels | 20195290 |
| rs137852808 | CHRNA1 | hmg coa reductase inhibitors | High Cholesterol levels | 22688219 |
| rs9930761 | CETP | hmg coa reductase inhibitors | High Cholesterol levels | 24080640 |
| rs629301 | CELSR2 | hmg coa reductase inhibitors | High Cholesterol levels | 27648687 |
| HLA-DRB1*01:01:01 | HLA-DRB1 | hmg coa reductase inhibitors | High Cholesterol levels | 27839692 |
| rs118192172 | RYR1 | hmg coa reductase inhibitors | High Cholesterol levels | 21795085 |
| rs352046 | CXCL5 | hmg coa reductase inhibitors | High Cholesterol levels | 18769620 |
| rs4823613 | PPARA | hmg coa reductase inhibitors | High Cholesterol levels | 23930676 |
| rs12654264 | HMGCR | hmg coa reductase inhibitors | High Cholesterol levels | 20403997 |
| rs11591147 | PCSK9 | hmg coa reductase inhibitors | High Cholesterol levels | 26902539 |
| rs247616 | - | hmg coa reductase inhibitors | High Cholesterol levels | 27587472 |
| rs17171676 | SUGCT | hmg coa reductase inhibitors | High Cholesterol levels | 27045730 |
| rs708272 | CETP | hmg coa reductase inhibitors | High Cholesterol levels | 14660992 |
| rs13064411 | CFAP44 | hmg coa reductase inhibitors | High Cholesterol levels | 25602530 |
| rs1050450 | RHOA | hmg coa reductase inhibitors | High Cholesterol levels | 22890915 |
| rs1799963 | F2 | hormonal contraceptives for systemic use | Pain symptoms | 12069454;15208046;16769590;28750087 |
| rs8176719 | ABO | hormonal contraceptives for systemic use | Pain symptoms | 28750087 |
| rs7297610 | - | hydrochlorothiazide | Hypertension | 18591461;22350108 |
| rs12279250 | NELL1 | hydrochlorothiazide | Hypertension | 23400010 |
| rs1159744 | WNK1 | hydrochlorothiazide | Hypertension | 16172412 |
| rs4506565 | TCF7L2 | hydrochlorothiazide | Hypertension | 24128935 |
| rs880054 | WNK1 | hydrochlorothiazide | Hypertension | 18591455 |
| rs11189015 | SLIT1 | hydrochlorothiazide | Hypertension | 25695618 |
| rs177852 | TTC6 | hydrochlorothiazide | Hypertension | 27802415 |
| rs658903 | KCNJ1 | hydrochlorothiazide | Hypertension | 22907731 |
| rs10752271 | CAMK1D | hydrochlorothiazide | Hypertension | 25410890 |
| rs11065987 | - | hydrochlorothiazide | Hypertension | 29925376 |
| rs10995 and rs10156 | VASP | hydrochlorothiazide | Hypertension | 28115488 |
| rs6947309 | FMC1 | hydrochlorothiazide | Hypertension | 24612202 |
| rs10792367 | SLC22A8 | hydrochlorothiazide | Hypertension | 21164499 |
| CYP2D6 extensive metabolizer phenotype | CYP2D6 | hydrocodone | Pain symptom | 7693389 |
| rs2372536 | ATIC | hydroxychloroquine | Malaria | 18322994 |
| rs9483947 & rs9376230 | MAP3K5 | hydroxyurea | Cancer | 23556445 |
| rs10901080 | ASS1 | hydroxyurea | Cancer | 26895070 |
| rs4671393 | BCL11A | hydroxyurea | Cancer | 28639471 |
| rs7166737 | SIN3A | hydroxyurea | Cancer | 27767389 |
| V600E | BTK | ibrutinib | Cancer | 24697238;24869598 |
| CYP2C9*3 | CYP2C9 | ibuprofen | Inflammation | 25502615 |
| rs4933824 | NRG3 | iloperidone | Psychiatric Disorders | 18521091 |
| rs1800169 | ZFP91 | iloperidone | Psychiatric Disorders | 18303965 |
| rs993648 | CERKL | iloperidone | Psychiatric Disorders | 18521091 |
| rs1050152 | SLC22A4 | imatinib | Cancer | 22875622;23127916 |
| rs1050152 | SLC22A4 | imatinib | Cancer | 22875622;23127916 |
| rs683369 | SLC22A1 | imatinib | Cancer | 24589908;28289867;28762371 |
| exons 11 (L576P) and 13 (K642E) | KIT | imatinib | Cancer | 24531699;11526490 |
| rs2631367 and rs2631372 | SLC22A5 | imatinib | Cancer | 23127916;28762371 |
| GSTM1del | GSTM1 | imatinib | Cancer | 25188725 |
| rs1058930 | CYP2C8 | imatinib | Cancer | 28383355 |
| rs10258429 | EGFR | imatinib | Cancer | 28762371 |
| rs9561765 | ABCC4 | imatinib | Cancer | 23127916 |
| rs2725252 | ABCG2 | imatinib | Cancer | 24123600 |
| G15631T | CYP2B6 | imatinib | Cancer | 24293093 |
| rs4148978 | SLCO1A2 | imatinib | Cancer | 21633340 |
| rs58818712 | ABCG2 | imatinib | Cancer | 24322003 |
| FIP1L1-PDGFR | FIP1L1 | imatinib | Cancer | 24433361 |
| rs150929 | ABCA3 | imatinib | Cancer | 25056761 |
| rs724710 | BCL2L11 | imatinib | Cancer | 24223824 |
| rs2290573 | ULK3 | imatinib | Cancer | 15073101 |
| rs121913512 | KIT | imatinib | Cancer | 11526490 |
| rs2282143 | SLC22A1 | imatinib | Cancer | 22875622 |
| rs1800750 | LTA | infliximab | Cancer | 22960943 |
| rs13266634 | SLC30A8 | insulin recombinant | Diabetes | 25348609 |
| rs34241435 | SCNN1B | Insulins And Analogues | Diabetes | 18004211 |
| rs10494227 | GAPDHP33 | interferon beta-1a | Infection | 27001119 |
| rs4961252 | - | interferon beta-1a | Infection | 21502966 |
| rs12044852 | CD58 | interferon beta-1a | Infection | 25685741 |
| rs1367117 | APOB | irbesartan | Hypertension | 15453913 |
| rs72558187 | CYP2C9 | irbesartan | Hypertension | 21842338 |
| rs1801701 | APOB | irbesartan | Hypertension | 15614026 |
| rs17287570 | ABCC1 | irinotecan | Cancer | 19696792 |
| rs6072262 | TOP1 | irinotecan | Cancer | 18347181 |
| rs3918305 | SVOP | irinotecan | Cancer | 25127363 |
| rs212090 | ABCC1 | irinotecan | Cancer | 27845419 |
| rs1517114 | C8orf34 | irinotecan | Cancer | 22664479 |
| rs10841661 | SLCO1B3 | irinotecan | Cancer | 19696792 |
| rs10934498 | NR1I2 | irinotecan | Cancer | 27116457 |
| rs425215 | ABCG1 | irinotecan | Cancer | 21892003 |
| rs3087465 | TGFBR2 | irinotecan | Cancer | 27160286 |
| rs4149015 | SLCO1B1 | irinotecan | Cancer | 18221820 |
| rs11574077 | VDR | irinotecan | Cancer | 29706892 |
| rs118192123 | RYR1 | isoflurane | Anesthetic | 15108991 |
| NAT2*12A | NAT2 | isoniazid | Tuberculosis | 21558457;24533708 |
| rs11125883 | XPO1 | isoniazid | Tuberculosis | 22341855;29036176 |
| rs11125883 | XPO1 | isoniazid | Tuberculosis | 22341855;29036176 |
| rs4720833 | MAFK | isoniazid | Tuberculosis | 22341855;29036176 |
| rs11080344 | NOS2 | isoniazid | Tuberculosis | 22341855;29036176 |
| NAT2*13A | NAT2 | isoniazid | Tuberculosis | 21558457;24533708 |
| rs2070401 | BACH1 | isoniazid | Tuberculosis | 22341855;29036176 |
| rs2070401 | BACH1 | isoniazid | Tuberculosis | 22341855;29036176 |
| rs4147581 | GSTP1 | isoniazid | Tuberculosis | 27281183 |
| NAT2*14A | NAT2 | isoniazid | Tuberculosis | 21558457 |
| NAT2*6J | NAT2 | isoniazid | Tuberculosis | 24533708 |
| rs1968753 | CES1 | isoniazid | Tuberculosis | 29036176 |
| NAT2*19 | NAT2 | isoniazid | Tuberculosis | 23150149 |
| rs2266782 | FMO3 | itopride | Gastrointestinal disease | 25224784 |
| rs80282562 | CFTR | ivacaftor | cystic fibrosis | 22293084;25266159 |
| rs121909041 | CFTR | ivacaftor | cystic fibrosis | 22293084;25266159 |
| rs121909005 | CFTR | ivacaftor | cystic fibrosis | 22293084;25266159;26474553 |
| rs121908757 | CFTR | ivacaftor | cystic fibrosis | 22293084;25266159;26474553;28947035 |
| rs121909047 | CFTR | ivacaftor | cystic fibrosis | 23891399;24902474 |
| rs77932196 | CFTR | ivacaftor | cystic fibrosis | 23891399;27334259;27812499 |
| rs113993960 | CFTR | ivacaftor | cystic fibrosis | 19846789;21602569;22293084;22383668;22942289;23891399;25148434;26968770;27214033;28325531;28930490;29099333;29099344 |
| rs74503330 | CFTR | ivacaftor | cystic fibrosis | 22293084;25266159;27160424 |
| rs75527207 | CFTR | ivacaftor | cystic fibrosis | 19846789;21083385;22047557;22293084;22942289;23313410;23590265;23757359;23757361;23891399;24066763;24461666;25049054;25145599;25148434;25171465;25311995;25473543;25682022;25755212;26135562;26568242;27158673;27413118;27745802;27773592;28611235;28651844;28711222;28930490 |
| rs121909013 | CFTR | ivacaftor | cystic fibrosis | 22293084;25266159 |
| rs121909011 | CFTR | ivacaftor | cystic fibrosis | 23891399;27334259;27812499 |
| rs397508442 | CFTR | ivacaftor | cystic fibrosis | 23891399;29279204 |
| rs74551128 | CFTR | ivacaftor | cystic fibrosis | 23891399;27334259 |
| rs193922525 | CFTR | ivacaftor | cystic fibrosis | 22293084;25266159 |
| rs121908755 | CFTR | ivacaftor | cystic fibrosis | 22293084;23027855;24081349;25266159;28371569 |
| rs78655421 | CFTR | ivacaftor | cystic fibrosis | 23891399;25698453;26070913;26324139;28419121 |
| rs368505753 | CFTR | ivacaftor | cystic fibrosis | 23891399 |
| rs397508602 | CFTR | ivacaftor | cystic fibrosis | 27334259 |
| rs267606723 | CFTR | ivacaftor | cystic fibrosis | 22293084 |
| 508del(CTT) | CFTR | ivacaftor | cystic fibrosis | 27160424 |
| rs7512462 | SLC26A9 | ivacaftor | cystic fibrosis | 28171547 |
| rs397508453 | CFTR | ivacaftor | cystic fibrosis | 25266159 |
| rs1019385 | GRIN2B | ketamine | Anesthetic | 28252572 |
| rs2562456 | LINC00664 | ketorolac | Inflammation | 19207018 |
| rs212091 | ABCC1 | lamivudine | Viral infection | 23996099 |
| HLA-B*35:05:01 | HLA-B | lamivudine | Viral infection | 25361850 |
| rs145450955 | SLC22A2 | lamivudine | Viral infection | 23252721 |
| HLA-C*07:18 | HLA-C | lamotrigine | Seizures, Epilepsy | 19668019 |
| UGT1A4*2 | UGT1A4 | lamotrigine | Seizures, Epilepsy | 22047493 |
| HLA-A*68:01:01:01 | HLA-A | lamotrigine | Seizures, Epilepsy | 19668019 |
| HLA-A*23:01:01 | HLA-A | lamotrigine | Seizures, Epilepsy | 25495410 |
| UGT1A4*1a | UGT1A4 | lamotrigine | Seizures, Epilepsy | 22047493 |
| HLA-DRB1*07:01:01:01 | HLA-DRB1 | lapatinib | Cancer | 24687830;25987243;28786423 |
| HLA-DQA1*02:01 | HLA-DQA1 | lapatinib | Cancer | 21245432;24687830 |
| HLA-DQA1*02:01 | HLA-DQA1 | lapatinib | Cancer | 21245432;24687830 |
| exon 9 (E542K, E545K, E547K, A533V, A533T), exon 20(H104R, H104L, G1007S) | PIK3CA | lapatinib | Cancer | 25199759 |
| rs2279238 | NR1H3 | laropiprant | High Cholesterol levels | 26226939 |
| rs3766355 | PTGFR | latanoprost | Glaucoma | 25339146;27336732 |
| rs3753380, rs3766355 | PTGFR | latanoprost | Glaucoma | 17467803;25339146;27336732 |
| rs3753380 | PTGFR | latanoprost | Glaucoma | 17467803;25339146 |
| rs10306114 | PTGS1 | latanoprost | Glaucoma | 25339146 |
| rs3213422 | DHODH | leflunomide | Rheumatoid arthritis | 19207032 |
| rs4135385 | CTNNB1 | lenalidomide | Multiple Myeloma | 26521987 |
| rs308395 | FGF2 | lenalidomide | Multiple Myeloma | 28373444 |
| CYP2A6*35A | CYP2A6 | letrozole | Cancer | 21975350 |
| rs3759811 | CYP19A1 | letrozole | Cancer | 26463708 |
| CYP2A6*1B1 | CYP2A6 | letrozole | Cancer | 21494765 |
| CYP2A6*23 | CYP2A6 | letrozole | Cancer | 21975350 |
| 638G>C | OPRT | leucovorin | Cancer | 20647221 |
| rs4704559 | HOMER1 | levodopa | Neurodegenerative disease, Parkinson's Disease | 24126708 |
| rs225014 | DIO2 | levothyroxine | hypothyroidism | 24910925 |
| rs117412760 | NOX3 | levothyroxine | hypothyroidism | 28486791 |
| rs10305420 | GLP1R | liraglutide | Diabetes | 25991051 |
| rs12941497 | NR1D1 | lithium | Psychiatric disorders | 19818381;20348464 |
| rs1801260 | CLOCK | lithium | Psychiatric disorders | 21047205;21781277 |
| rs2071427 | NR1D1 | lithium | Psychiatric disorders | 20348464;21781277 |
| rs2284017 | CACNG2 | lithium | Psychiatric disorders | 18408563;21047205 |
| rs6438552 | GSK3B | lithium | Psychiatric disorders | 21781277;23021822 |
| rs1387923 | NTRK2 | lithium | Psychiatric disorders | 21047205;23315174 |
| rs2314339 | NR1D1 | lithium | Psychiatric disorders | 20348464 |
| rs228642 | PER3 | lithium | Psychiatric disorders | 21781277 |
| rs2769605 | - | lithium | Psychiatric disorders | 23315174 |
| rs3919583 | - | lithium | Psychiatric disorders | 29121268 |
| rs3730353 | FYN | lithium | Psychiatric disorders | 21047205 |
| rs9315885 | DGKH | lithium | Psychiatric disorders | 19818381 |
| rs4512905 | SCN8A | lithium | Psychiatric disorders | 21961650 |
| rs74795342 | AL157359.1 | lithium | Psychiatric disorders | 26806518 |
| rs2284018 | CACNG2 | lithium | Psychiatric disorders | 18408563 |
| rs4948102 | PSPH | l-methionine | liver disorder, viral infections | 24651765 |
| rs11623866 | FNTB | lonafarnib | Cancer | 26033044 |
| rs4149045 | SLCO1B1 | lopinavir | Viral infection | 27142945 |
| CYP2C9*13 | CYP2C9 | lornoxicam | Inflammation | 15764711;16182270 |
| rs1934969 | CYP2C9 | losartan | Hypertension | 22294058;25303293 |
| CYP2C9*5 | CYP2C9 | losartan | Hypertension | 15197523;15289788;22735459 |
| rs3814995 | NPHS1 | losartan | Hypertension | 25622599;28353407 |
| rs3814995 | NPHS1 | losartan | Hypertension | 25622599;28353407 |
| rs1529909 | SLC22A12 | losartan | Hypertension | 26086348 |
| rs12721226 | AGTR1 | losartan | Hypertension | 20436376 |
| CYP2C9*30 | CYP2C9 | losartan | Hypertension | 18971529 |
| rs6749447 | STK39 | losartan | Hypertension | 21993215 |
| rs9332174 | CYP2C9 | losartan | Hypertension | 22294058 |
| rs2291073 | SLCO1B1 | lovastatin | High Cholesterol levels | 16103896 |
| rs41303343 | CYP3A5 | lumefantrine | Malaria | 28673292 |
| HLA-DQA1*01:01:01 | HLA-DQA1 | lumiracoxib | Inflammation | 20639878 |
| rs4240803 | SLC7A5 | melphalan | Cancer | 24704384 |
| BCHE-K allele | BCHE | memantine | Dementia, Alzheimer' Disease | 25376930 |
| rs41291556 | CYP2C19 | mephenytoin | Seizures, Epilepsy | 10411572;21325430 |
| CYP2C19*33 | CYP2C19 | mephenytoin | Seizures, Epilepsy | 26153442 |
| rs55948420 | CYP2C19 | mephenytoin | Seizures, Epilepsy | 19661214 |
| TPMT*33 | TPMT | mercaptopurine | Cancer | 12172211;13679074;15083071;15652243;16220112;18602085;18708949;19660010;19945438;20881512;20945351;23252716;24710034;25441457;9246020;9711875 |
| TPMT*13 | TPMT | mercaptopurine | Cancer | 12172211;13679074;15083071;15652243;16220112;18602085;18708949;19660010;19945438;20881512;20945351;23252716;24710034;25441457;9246020;9711875 |
| TPMT*25 | TPMT | mercaptopurine | Cancer | 12172211;13679074;15083071;15652243;16220112;18602085;18708949;19660010;19945438;20881512;20945351;23252716;24710034;25441457;9246020;9711875 |
| TPMT*10 | TPMT | mercaptopurine | Cancer | 12172211;13679074;15083071;15652243;16220112;18602085;18708949;19660010;19945438;20881512;20945351;23252716;24710034;25441457;9246020;9711875 |
| rs2413739 | PACSIN2 | mercaptopurine | Cancer | 22846425;27452984 |
| TPMT*15 | TPMT | mercaptopurine | Cancer | 12172211;13679074;15083071;15652243;16220112;18602085;18708949;19660010;19945438;20881512;20945351;23252716;24710034;25441457;9246020;9711875 |
| TPMT*28 | TPMT | mercaptopurine | Cancer | 12172211;13679074;15083071;15652243;16220112;18602085;18708949;19660010;19945438;20881512;20945351;23252716;24710034;25441457;9246020;9711875 |
| NUDT15*4 | NUDT15 | mercaptopurine | Cancer | 26878724;28146264;28445187;28659275;29923122 |
| rs2413739 | PACSIN2 | mercaptopurine | Cancer | 22846425;27452984 |
| TPMT*34 | TPMT | mercaptopurine | Cancer | 12172211;13679074;15083071;15652243;16220112;18602085;18708949;19660010;19945438;20881512;20945351;23252716;24710034;25441457;9246020;9711875 |
| TPMT*37 | TPMT | mercaptopurine | Cancer | 12172211;13679074;15083071;15652243;16220112;18602085;18708949;19660010;19945438;20881512;20945351;23252716;24710034;25441457;9246020;9711875 |
| TPMT*32 | TPMT | mercaptopurine | Cancer | 12172211;13679074;15083071;15652243;16220112;18602085;18708949;19660010;19945438;20881512;20945351;23252716;24710034;25441457;9246020;9711875 |
| TPMT*1A | TPMT | mercaptopurine | Cancer | 12172211;13679074;15083071;15652243;16220112;18602085;18708949;19660010;19945438;20881512;20945351;23252716;24710034;25441457;9246020;9711875 |
| TPMT*26 | TPMT | mercaptopurine | Cancer | 12172211;13679074;15083071;15652243;16220112;18602085;18708949;19660010;19945438;20881512;20945351;23252716;24710034;25441457;9246020;9711875 |
| TPMT*27 | TPMT | mercaptopurine | Cancer | 12172211;13679074;15083071;15652243;16220112;18602085;18708949;19660010;19945438;20881512;20945351;23252716;24710034;25441457;9246020;9711875 |
| TPMT*31 | TPMT | mercaptopurine | Cancer | 12172211;13679074;15083071;15652243;16220112;18602085;18708949;19660010;19945438;20881512;20945351;23252716;24710034;25441457;9246020;9711875 |
| TPMT*19 | TPMT | mercaptopurine | Cancer | 12172211;13679074;15083071;15652243;16220112;18602085;18708949;19660010;19945438;20881512;20945351;23252716;24710034;25441457;9246020;9711875 |
| TPMT*5 | TPMT | mercaptopurine | Cancer | 12172211;13679074;15083071;15652243;16220112;18602085;18708949;19660010;19945438;20881512;20945351;23252716;24710034;25441457;9246020;9711875 |
| TPMT*30 | TPMT | mercaptopurine | Cancer | 12172211;13679074;15083071;15652243;16220112;18602085;18708949;19660010;19945438;20881512;20945351;23252716;24710034;25441457;9246020;9711875 |
| TPMT*24 | TPMT | mercaptopurine | Cancer | 12172211;13679074;15083071;15652243;16220112;18602085;18708949;19660010;19945438;20881512;20945351;23252716;24710034;25441457;9246020;9711875 |
| rs7043257 | SLC28A3 | mercaptopurine | Cancer | 24624911 |
| rs2842949 | TPMT | mercaptopurine | Cancer | 18605963 |
| rs10948059 | PEX6 | mercaptopurine | Cancer | 27452984 |
| rs766023281 | NUDT15 | mercaptopurine | Cancer | 28659275 |
| C1561T | GCPII | mercaptopurine | Cancer | 22838948 |
| rs2842934 | TPMT | mercaptopurine | Cancer | 18605963 |
| TPMT extensive metabolizers | TPMT | mercaptopurine | Cancer | 18987654 |
| TPMT intermediate activity | TPMT | mercaptopurine | Cancer | 15784872 |
| rs11045879 | SLCO1B1 | mercaptopurine | Cancer | 22846425 |
| NAT1*10 | NAT1 | mesalazine | Inflammation | 25834322 |
| rs72552763 | SLC22A1 | metformin | Diabetes | 21989078;23873119;27859023;28380657;28834135 |
| rs784888 | AMHR2 | metformin | Diabetes | 24853734;29352482 |
| rs2252281 | SLC47A1 | metformin | Diabetes | 23267855;23873119;25939711;27859023;28834135 |
| rs11212617 | C11orf65 | metformin | Diabetes | 21186350;22453232;22751958;27386433;28834135;29790415 |
| rs11212617 | C11orf65 | metformin | Diabetes | 21186350;22453232;22751958 |
| rs784888 | AMHR2 | metformin | Diabetes | 24853734;29352482 |
| rs12943590 | SLC47A2 | metformin | Diabetes | 21956618;23267855;23417334;23652408;25939711;27859023;28834135 |
| rs784892 | AMHR2 | metformin | Diabetes | 24853734;29352482 |
| rs13376631 | FMO1 | metformin | Diabetes | 26306225 |
| rs391300 | SRR | metformin | Diabetes | 21933224 |
| rs254271 | PRPF31 | metformin | Diabetes | 29650774 |
| SLC19A3 | SLC19A3 | metformin | Diabetes | 26528626 |
| rs10747673 | - | metformin | Diabetes | 24853734 |
| rs10783050 | - | metformin | Diabetes | 26401715 |
| rs8192675 | SLC2A2 | metformin | Diabetes | 27500523 |
| rs316009 | SLC22A2 | metformin | Diabetes | 27415606 |
| rs6719578 | SAP18P2 | metformin | Diabetes | 28453780 |
| rs2815752 | AL513166.1 | metformin | Diabetes | 22179955 |
| rs34834489 | SLC47A2 | metformin | Diabetes | 23652408 |
| rs249429 | PRKAA1 | metformin | Diabetes | 25327507 |
| rs2464196 | HNF1A | metformin | Diabetes | 28834135 |
| rs2289669 | SLC47A1 | metformin | Diabetes | 22722338 |
| (rs2252281 and rs12943590 | MATE1, MATE2 | metformin | Diabetes | 25939711 |
| rs594709 | SLC22A1 | metformin | Diabetes | 26977146 |
| rs1801282 | PPARG | metformin | Diabetes | 22179955 |
| rs272893 | SLC22A4 | metformin | Diabetes | 27859023 |
| rs36056065 | SLC22A1 | metformin | Diabetes | 22735389 |
| rs391300 | SRR | metformin | Diabetes | 21933224 |
| rs34399035 | ALDH3A2 | metformin | Diabetes | 21956618 |
| rs2076828 | SLC22A3 | metformin | Diabetes | 25920679 |
| rs2239622 | NGF | methadone | Pain symptom | 21358750;23651024 |
| rs2239622 | NGF | methadone | Pain symptom | 21358750;23651024 |
| rs4358872 | NTRK2 | methadone | Pain symptom | 23651024 |
| rs7662029 | UGT2B7 | methadone | Pain symptom | 22676193 |
| rs1129844 | CCL11 | methadone | Pain symptom | 29222992 |
| rs8094439 and rs17446819 | CDH2 | methadone | Pain symptom | 28358908 |
| rs34230287 | ARRB2 | methadone | Pain symptom | 20514076 |
| rs1861591 | CRY1 | methadone | Pain symptom | 20560679 |
| rs17180299 | - | methadone | Pain symptom | 27010727 |
| rs11030119 | BDNF | methadone | Pain symptom | 18182069 |
| rs1714984 | MYOCD | methadone | Pain symptom | 20560679 |
| rs2760118 | ALDH5A1 | methadone | Pain symptom | 24230997 |
| rs9479757 | OPRM1 | methadone | Pain symptom | 21902500 |
| rs3735451 | CYP3A4 | methadone | Pain symptom | 21902501 |
| rs2619538 | DTNBP1 | methamphetamine | psychiatric Disorders | 17555717 |
| rs2248829 | SLC6A9 | methamphetamine | psychiatric Disorders | 17582620 |
| rs3924999 | NRG1 | methamphetamine | psychiatric Disorders | 19394386 |
| rs135745 | CSNK1E | methamphetamine | psychiatric Disorders | 18991847 |
| rs2236418 | GAD2 | methamphetamine | psychiatric Disorders | 27967329 |
| rs3916965 | - | methamphetamine | psychiatric Disorders | 19482054 |
| rs2076369 | PICK1 | methamphetamine | psychiatric Disorders | 17606663 |
| rs878567 | HTR1A | methamphetamine | psychiatric Disorders | 19747927 |
| rs4790694 | ARRB2 | methamphetamine | psychiatric Disorders | 17233643 |
| rs1126442 | GRIN1 | methamphetamine | psychiatric Disorders | 23880023 |
| rs135745 | CSNK1E | methamphetamine | psychiatric Disorders | 18991847 |
| rs35592 | ABCC1 | methotrexate | cancer | 18256692;29743634 |
| rs6064463 | - | methotrexate | cancer | 24583629;29743634 |
| rs1801394 | PTPRM | methotrexate | cancer | 24583629;29743634 |
| rs10106 | ENG | methotrexate | cancer | 26652611;27992285 |
| rs9516519 | ABCC4 | methotrexate | cancer | 23222202;24404132 |
| rs1901633 | AC018978.1 | methotrexate | cancer | 24583629;29743634 |
| rs7563206 | ATIC | methotrexate | cancer | 22450926;27676277 |
| rs3821353 | ATIC | methotrexate | cancer | 22450926;27676277 |
| rs5760410 | SPECC1L | methotrexate | cancer | 18539621;27399166 |
| rs4982133 | EGLN3 | methotrexate | cancer | 24583629;29743634 |
| rs246240 | ABCC1 | methotrexate | cancer | 18256692;26086825 |
| rs1544105 | FPGS | methotrexate | cancer | 19902562;22450926;26652611;27992285 |
| rs3758149 | GGH | methotrexate | cancer | 15564880;19827168;26616421 |
| rs2650972 | - | methotrexate | cancer | 24583629;29743634 |
| rs7624766 | - | methotrexate | cancer | 24583629;29743634 |
| rs66554220 | - | methotrexate | cancer | 16906016;19088262 |
| rs4673993 | ATIC | methotrexate | cancer | 19193698;20847201;27676277;27992285 |
| rs6506569 | PTPRM | methotrexate | cancer | 24583629;29743634 |
| rs1703794 | AC036108.1 | methotrexate | cancer | 24583629;29743634 |
| rs4451422 | ENG | methotrexate | cancer | 22450926 |
| rs11702425 | COL18A1 | methotrexate | cancer | 22450926 |
| rs2238476 | ABCC1 | methotrexate | cancer | 18256692 |
| rs1544105 | FPGS | methotrexate | cancer | 19902562 |
| rs17421511 | MTHFR | methotrexate | cancer | 25084201 |
| KIR2DL2 | KIR2DL2 | methotrexate | cancer | 27251940 |
| rs10280623 | ABCB1 | methotrexate | cancer | 26652611 |
| rs3761548 | FOXP3 | methotrexate | cancer | 28444425 |
| rs4846051 | C1orf167 | methotrexate | cancer | 16439441 |
| rs1053129 | DHFR | methotrexate | cancer | 25778468 |
| SLCO1B1*23 | SLCO1B1 | methotrexate | cancer | 22147369 |
| rs56292801 | ZFPM1 | methotrexate | cancer | 27649261 |
| rs3812265 | CNOT4 | methotrexate | cancer | 28628559 |
| KIR2DS4 | KIR2DS4 | methotrexate | cancer | 25069714 |
| rs12517451 | DHFR | methotrexate | cancer | 22450926 |
| rs11045872 | SLCO1B1 | methotrexate | cancer | 23233662 |
| rs1105525 | MSH3 | methotrexate | cancer | 19861437 |
| rs4888024 | NA | methotrexate | cancer | 19176441 |
| rs10841753 | SLCO1B1 | methotrexate | cancer | 28525903 |
| rs9624472 | SLC46A1 | methotrexate | cancer | 21317831 |
| rs1800909 | GGH | methotrexate | cancer | 17286537 |
| rs3763980, rs10877333 | SLC16A7 | methotrexate | cancer | 20827233 |
| rs3768142 | MTR | methotrexate | cancer | 24712521 |
| rs2236624 | ADORA2A-AS1 | methotrexate | cancer | 18539621 |
| rs3763980 | SLC16A7 | methotrexate | cancer | 20827233 |
| rs408626 | MSH3 | methotrexate | cancer | 26335211 |
| rs868853 | ABCC4 | methotrexate | cancer | 24404132 |
| rs4148396 | ABCC2 | methotrexate | cancer | 18381794 |
| rs10994982 | ARID5B | methotrexate | cancer | 19684603 |
| rs719235 | GGH | methotrexate | cancer | 22763757 |
| rs7317112 | ABCC4 | methotrexate | cancer | 25348617 |
| rs2372536, rs3821353, rs7563206, rs12995526, rs2267076 | ATIC,ADORA2A | methotrexate | cancer | 27676277 |
| rs1232027 | LINC01337 | methotrexate | cancer | 20472929 |
| rs9895420 | ABCC3 | methotrexate | cancer | 21606946 |
| SLCO1B1*35 | SLCO1B1 | methotrexate | cancer | 22147369 |
| rs3784864 | ABCC1 | methotrexate | cancer | 26086825 |
| rs3045983 | MSH3 | methotrexate | cancer | 27636122 |
| BLVRB deficiency | BLVRB | methylene blue | Infection | 23913015 |
| exon 3 48-bp VNTR | DRD4 | methylphenidate | Psychiatric disorder | 23856854 |
| rs2071421 | ARSA | methylphenidate | Psychiatric disorder | 29382897 |
| rs828199 | - | methylphenidate | Psychiatric disorder | 20863575 |
| rs11134178 | LINC02236 | methylphenidate | Psychiatric disorder | 18821564 |
| rs3815589 | 0 | methylphenidate | Psychiatric disorder | 24350812 |
| rs1355368 | ADGRL3 | methylphenidate | Psychiatric disorder | 25989180 |
| rs6090384 | AL121827.1 | methylphenidate | Psychiatric disorder | 17948872 |
| CYP2D6 extensive metabolizer | CYP2D6 | methylphenidate | Psychiatric disorder | 10831022 |
| rs2284411 | GRIN2B | methylphenidate | Psychiatric disorder | 27624150 |
| CYB5R3 deficiency | CYB5R3 | metoclopramide | Nausea | 11418378 |
| CYP2D6*20 | CYP2D6 | metoprolol | Hypertension | 10471072 |
| CYP2D6*2 | CYP2D6 | mianserin | Psychiatric Disorders | 14652703 |
| rs1464602 | NR1I2 | midazolam | Psychiatric disorders | 17050801 |
| rs1464603 | NR1I2 | midazolam | Psychiatric disorders | 17050801 |
| rs12721627 | CYP3A4 | midazolam | Psychiatric disorders | 19255940 |
| rs1160351 | MDGA2 | milnacipran | Psychiatric Disorders | 22445761 |
| rs1364043 | NA | milnacipran | Psychiatric Disorders | 18484082 |
| rs10879346 | TPH2 | mirtazapine | Psychiatric Disorders | 18496129 |
| CYP2B6*7 | CYP2B6 | mirtazapine | Psychiatric Disorders | 22926595 |
| rs1487278 | TPH2 | mirtazapine | Psychiatric Disorders | 18496129 |
| rs4149087 | SLCO1B1 | misoprostol | abortion, ulcer | 26122863 |
| rs1799807 | BCHE | mivacurium | Anesthesia | 12881446 |
| rs755648929 | BCHE | mivacurium | Anesthesia | 27031121 |
| rs12422149 | SLCO2B1 | montelukast | Asthma | 19151602;23970434 |
| rs2115819 | ALOX5 | montelukast | Asthma | 16293801 |
| rs2712807 | SLCO2B1 | montelukast | Asthma | 23970434 |
| rs1364805 | AC092445.1 | montelukast | Asthma | 26083242 |
| rs11576941 | FAAH | morphine | Pain symptom | 25558980;27977335 |
| rs2295632 | FAAH | morphine | Pain symptom | 25558980;27977335 |
| rs2075507 | TXNRD2 | morphine | Pain symptom | 19094200 |
| rs1051660 | OPRK1 | morphine | Pain symptom | 29259946 |
| rs3766246 | FAAH | morphine | Pain symptom | 25558980 |
| rs6699322 | FAAH | morphine | Pain symptom | 27977335 |
| rs165728 | COMT | morphine | Pain symptom | 19094200 |
| rs174699 | COMT | morphine | Pain symptom | 19094200 |
| rs7668282 | UGT2B7 | morphine | Pain symptom | 17724700 |
| rs2368564 | REN | muraglitazar | Diabetes | 18794727 |
| rs11572076 | CYP2C8 | mycophenolate mofetil | transplant rejection | 21107304;24681964 |
| rs2037483 | HUS1 | mycophenolate mofetil | transplant rejection | 21107304 |
| rs4974081 | IMPDH2 | mycophenolate mofetil | transplant rejection | 20679962 |
| rs6714486 | UGT1A10 | mycophenolic acid | transplant rejection | 22765258 |
| rs2741046 | UGT1A10 | mycophenolic acid | transplant rejection | 25163792 |
| rs2280055, rs3894049 | NFATC1 | mycophenolic acid | transplant rejection | 28244807 |
| rs4654327 | OPRD1 | naltrexone | Alcoholism | 22954510 |
| CYP2D6*24 | CYP2D6 | n-desmethyltamoxifen | Cancer | 18838503;24647041 |
| CYP2D6*52 | CYP2D6 | n-desmethyltamoxifen | Cancer | 24647041 |
| rs12721646 | CYP2B6 | nevirapine | Viral infection | 20017669;23774940 |
| rs58425034 | CYP2B6 | nevirapine | Viral infection | 20017669;23774940 |
| HLA-DRB1*01:02:01 | HLA-DRB1 | nevirapine | Viral infection | 23328091;28819312 |
| RS3099844 | HCP5 | nevirapine | Viral infection | 24322967;28689274 |
| HLA-A*24:07 | HLA-A | nevirapine | Viral infection | 19104471 |
| rs76228616 | TRAF3IP2 | nevirapine | Viral infection | 25775161 |
| HLA-C*18:01 | HLA-C | nevirapine | Viral infection | 28819312 |
| rs9461684 | RPL3P2 | nevirapine | Viral infection | 21810746 |
| HLA-B*15:10:01 | HLA-B | nevirapine | Viral infection | 23328091 |
| G516T and T983C | CYP2B6 | nevirapine | Viral infection | 23774940 |
| HLA-C*08:02:01 | HLA-C | nevirapine | Viral infection | 16868443 |
| rs1265112 | CCHCR1 | nevirapine | Viral infection | 21810746 |
| rs3786547 | CYP2B6 | nevirapine | Viral infection | 21505298 |
| HLA-DRB1*12:02:01 | HLA-DRB1 | nevirapine | Viral infection | 19104471 |
| rs12768009 | CYP2C19 | nevirapine | Viral infection | 21860339 |
| rs3842689 | NR1I2 | nevirapine | Viral infection | 22111602 |
| HLA-DRB1*15:03:01:01 | HLA-DRB1 | nevirapine | Viral infection | 23362284 |
| HLA-DQB1*06:03:01 | HLA-DQB1 | nevirapine | Viral infection | 23362284 |
| rs35979566 | CYP2B6 | nevirapine | Viral infection | 22354160 |
| CYP2A6*27 | CYP2A6 | nicotine | smoking cessation | 11278503;11779172;12445030;12844137;15900015;15940289;15993850;16402128;16952495;17267622;17522595;18360915;22569203;23371292;24448396;25416559;28181923 |
| rs12718541 | DDC | nicotine | smoking cessation | 16740595;21806388 |
| rs5031016 | CYP2A6 | nicotine | smoking cessation | 11779172;12445030 |
| CYP2A6 poor metabolizer genotype | CYP2A6 | nicotine | smoking cessation | 23371292;24448396 |
| rs1044397 | CHRNA4 | nicotine | smoking cessation | 19290018;21740768 |
| rs28399468 | CYP2A6 | nicotine | smoking cessation | 11779172;12445030 |
| rs2229959 | CHRNA4 | nicotine | smoking cessation | 19290018;20854418 |
| CYP2A6*1X2B | CYP2A6 | nicotine | smoking cessation | 11278503;11779172;12445030;12844137;15900015;15940289;15993850;16402128;16952495;17267622;17522595;18360915;22569203;23371292;24448396;25416559;28181923 |
| rs56113850 | CYP2A6 | nicotine | smoking cessation | 26407342;29232328 |
| rs3813567 | CHRNB4 | nicotine | smoking cessation | 20886544 |
| rs2072658 | CHRNB2 | nicotine | smoking cessation | 20854418 |
| rs921451 | DDC | nicotine | smoking cessation | 21806388 |
| rs17189632 | GRIN3A | nicotine | smoking cessation | 20084518 |
| rs4838547 | CHAT | nicotine | smoking cessation | 20383528 |
| rs143690364 | CYP2A6 | nicotine | smoking cessation | 24305170 |
| rs854568 | PON1 | nicotine | smoking cessation | 25741997 |
| rs113288603 | AC008537.1 | nicotine | smoking cessation | 26407342 |
| rs10912675 | FMO1 | nicotine | smoking cessation | 21540762 |
| rs6721498 | NRXN1 | nicotine | smoking cessation | 18270208 |
| rs55781567 | CHRNA5 | nicotine | smoking cessation | 29196725 |
| rs111645190 | AC008537.1 | nicotine | smoking cessation | 28921760 |
| rs324650 | CHRM2 | nicotine | smoking cessation | 19644963 |
| rs112561475 | UGT2B10 | nicotine | smoking cessation | 24192532 |
| CYP2A6 poor metabolizers | CYP2A6 | nicotine | smoking cessation | 25416559 |
| rs77905 | DBH | nicotine | smoking cessation | 15077009 |
| rs11101191 | CHAT | nicotine | smoking cessation | 20383528 |
| rs2273504 | AL121827.1 | nicotine | smoking cessation | 19290018 |
| rs56256500 | CYP2A6 | nicotine | smoking cessation | 18216723 |
| rs3735273 | DDC | nicotine | smoking cessation | 21806388 |
| rs1541333 | DBH | nicotine | smoking cessation | 24667010 |
| CYP2A6*28A | CYP2A6 | nicotine | smoking cessation | 18360915 |
| rs2060761 | DDC | nicotine | smoking cessation | 16740595 |
| rs2869950 | FAM13A | nicotine | smoking cessation | 23692359 |
| rs5031017 | CYP2A6 | nicotine | smoking cessation | 16952495 |
| rs4986913 | CYP3A4 | nifedipine | Cardiovascular disease | 15634941 |
| rs3745009 | SLC14A2 | nifedipine | Cardiovascular disease | 17344938 |
| rs1048101 | ADRA1A | nifedipine | Cardiovascular disease | 19444285 |
| rs5273 | PTGS2 | nimesulide | Pain symptom | 20548327 |
| CYP2D6 ultrarapid metabolizer genotype | CYP2D6 | noroxycodone | Pain symptom | 20590587 |
| rs2500535 | UST | nortriptyline | Psychiatric Disorders | 20360315;25916525 |
| CYP2D6 ultrarapid metabolizer phenotype | CYP2D6 | nortriptyline | Psychiatric Disorders | 24257813;4082245 |
| rs11195419 | ADRA2A | nortriptyline | Psychiatric Disorders | 19641488 |
| rs7148697, rs1887104, rs17097273, rs7146234, rs6573304, rs2273623, rs9323364 | PPM1A | nortriptyline | Psychiatric Disorders | 20970119 |
| CYP2D6*81 | CYP2D6 | nortriptyline | Psychiatric Disorders | 23799451 |
| rs2500535 | UST | nortriptyline | Psychiatric Disorders | 20360315 |
| rs17060812 | SLC39A14 | nortriptyline | Psychiatric Disorders | 20970119 |
| rs2853826 | MT-ND3 | Nucleoside and nucleotide reverse transcriptase inhibitors | viral infection, AIDS | 23944767 |
| rs757573592 | SLX1B | o-desmethyl-tramadol | Pain symptom | 29705271 |
| rs553050853 | SLX1A | o-desmethyl-tramadol | Pain symptom | 29705271 |
| rs2440390 | DRD2 | olanzapine | Psychiatric Disorders | 22967772 |
| rs2213712 | NA | olanzapine | Psychiatric Disorders | 21519338 |
| rs227092 | SV2C | olanzapine | Psychiatric Disorders | 23886675 |
| rs2283271 | CACNA1C | olanzapine | Psychiatric Disorders | 26049408 |
| rs2734841 | DRD2 | olanzapine | Psychiatric Disorders | 21095016 |
| rs4731426 | LEP | olanzapine | Psychiatric Disorders | 18681781 |
| rs7973796 and rs11111201 | PMCH | olanzapine | Psychiatric Disorders | 17541984 |
| rs11240594 | SLC26A9 | olanzapine | Psychiatric Disorders | 21107309 |
| rs12720462 | FMO1 | olanzapine | Psychiatric Disorders | 23147717 |
| rs1880676 | CHAT | olanzapine | Psychiatric Disorders | 17503482 |
| rs4410790 | AHR | olanzapine | Psychiatric Disorders | 23492908 |
| rs2527894 | Y_RNA | olanzapine | Psychiatric Disorders | 21519338 |
| rs2734842 | DRD2 | olanzapine | Psychiatric Disorders | 21095016 |
| rs2661319 and rs2842030 | RGS4 | olanzapine | Psychiatric Disorders | 17588543 |
| rs6314 | HTR2A | olanzapine | Psychiatric Disorders | 25563748 |
| rs75519181 | AHR | omeprazole | Stomach ulcers | 15860653 |
| rs1143634 | IL1B | omeprazole | Stomach ulcers | 21054464 |
| rs2066853 | AHR | omeprazole, benzidine, somatostatin | Cancer, acromegaly | 10739168;11207035;15860653;11866883; 30488289 |
| CYP2D6*102 | CYP2D6 | ondansetron | Nausea | 28002639 |
| rs1042173 | SLC6A4 | ondansetron | Nausea | 21247998 |
| CYP2D6*9 | CYP2D6 | ondansetron | Nausea | 28002639 |
| rs2070762 | INS | opioids | Pain symptom | 29210332 |
| rs1805007 | TUBB3 | opioids | Pain symptom | 24086514 |
| rs1997794, rs1022563 | PDYN | opioids | Pain symptom | 19298317 |
| rs6090041 | LKAAEAR1 | opioids | Pain symptom | 20032820 |
| rs1997794 | PDYN | opioids | Pain symptom | 19298317 |
| rs660652 | CHRNA5 | Opium alkaloids and derivatives | Pain symptom | 20725741 |
| rs121912777 | CES1 | oseltamivir | Viral infection | 22588607 |
| CYP2A6*4 alleles, CYP2A6*7, *9, and *10 | CYP2A6 | oxaliplatin | Cancer | 21326246;23963147 |
| rs830884 | AC022126.1 | oxaliplatin | Cancer | 27069012 |
| UGT1A1*60 | UGT1A1 | oxaliplatin | Cancer | 23963147 |
| CYP2A6*1A | CYP2A6 | oxaliplatin | Cancer | 21326246 |
| rs5275 | PTGS2 | oxaliplatin | Cancer | 19219602 |
| rs1846692 | MT1A | oxaliplatin | Cancer | 24924344 |
| rs1976391 | UGT1A6 | oxazepam | Psychiatric Disorders, Major Depression | 29737521 |
| HLA-B*27:09 | HLA-B | oxcarbazepine | Seizures, Epilepsy | 22818943 |
| HLA-DRB1*04:03:01 | HLA-DRB1 | oxcarbazepine | Seizures, Epilepsy | 27666425 |
| rs7016778 | OPRK1 | oxycodone | Pain symptom | 27472837 |
| rs7349683 | EPHA5 | paclitaxel | Cancer | 22843789;26133776;26133777;26763541 |
| rs1113129 | CYP2C8 | paclitaxel | Cancer | 20212519;25495407 |
| rs9657362 | ARHGEF10 | paclitaxel | Cancer | 25164601;26143528 |
| rs7349683 | EPHA5 | paclitaxel | Cancer | 22843789;26133776;26133777;26763541 |
| rs492338 | ABCG1 | paclitaxel | Cancer | 24706167 |
| rs10771973 | FGD4 | paclitaxel | Cancer | 22843789 |
| rs306005 | SPATA5 | paclitaxel | Cancer | 24444404 |
| rs209709 | EPHA8 | paclitaxel | Cancer | 26133777 |
| CYP3A4*8 | CYP3A4 | paclitaxel | Cancer | 25398452 |
| rs66501115 | CYP2C8 | paclitaxel | Cancer | 11767116 |
| rs56240334 | ADCK1 | paliperidone | Psychiatric Disorders | 27846195 |
| rs3212986 | ERCC1 | palonosetron | Nausea | 29177570 |
| CYP2D6 extensive metabolizer genotype | CYP2D6 | paramethoxymethamphetamine | psychiatric disorders | 26930544 |
| rs10789970 | HTR3B | paroxetine | Psychiatric Disorders, Major Depression | 17697394 |
| rs1800042 | HTR1A | paroxetine | Psychiatric Disorders, Major Depression | 20075642 |
| rs2216711, rs2973049 | GDNF | paroxetine | Psychiatric Disorders, Major Depression | 24577123 |
| rs11042725 | ADM | paroxetine | Psychiatric Disorders, Major Depression | 19636336 |
| UGT1A1*37 | UGT1A1 | pazopanib | Cancer | 20389299;24107802 |
| rs11549467 | HIF1A | pazopanib | Cancer | 21576632 |
| rs2858996 | HFE | pazopanib | Cancer | 21145803 |
| rs307821 and rs307826 | VEGFR3 | pazopanib | Cancer | 26063633 |
| rs3824662 | GATA3 | pegaspargase | Cancer | 24141364 |
| rs75161997 and rs1891059 | LINC00251 | pegaspargase | Cancer | 26590194 |
| rs6051702 | C20orf194 | peginterferon alfa-2a | Tumour | 25713999;27833958 |
| rs1127354 | ITPA | peginterferon alfa-2a | Tumour | 23133602;26441325;27833958 |
| rs368234815 | IFNL3 | peginterferon alfa-2a | Tumour | 23291588;23712427;24072198;24205831;24308755;24355007;24362944;24690180;24748394;25393304;25548683;26032235;26186989;26670100;26820907;26916827;27027531;27180197;27810523;28186161;28261910;28732143;28739427 |
| rs4803217 | IFNL3 | peginterferon alfa-2a | Tumour | 22328925;25287681;26186989;28638221 |
| rs12980275 | IFNL3 | peginterferon alfa-2a | Tumour | 19749757;21112657;21346780;21390311;21987611;24308755;25769643;27498543;27714501;28703131;28739427 |
| rs8105790 | IFNL3 | peginterferon alfa-2a | Tumour | 19749757;21346780 |
| rs8103142 | IFNL3 | peginterferon alfa-2a | Tumour | 19684573;19749757;23281610 |
| rs11881222 | IFNL3 | peginterferon alfa-2a | Tumour | 19749757;21346780;22328925;23281610 |
| rs28416813 | IFNL3 | peginterferon alfa-2a | Tumour | 19684573;19749757;22328925 |
| rs7248668 | MSRB1P1 | peginterferon alfa-2a | Tumour | 19749757;21346780;27498543 |
| rs4273729 | MTCO3P1 | peginterferon alfa-2a | Tumour | 22992578;26632884;27714501;28739427 |
| rs52797880 | APOH | peginterferon alfa-2a | Tumour | 26670100 |
| rs117648444 | IFNL3 | peginterferon alfa-2a | Tumour | 25534433 |
| rs10846744 | SCARB1 | peginterferon alfa-2a | Tumour | 27561198 |
| rs10883841 | NT5C2 | peginterferon alfa-2a | Tumour | 28453396 |
| rs2660 | OAS1 | peginterferon alfa-2a | Tumour | 23133602 |
| rs8109886 | MSRB1P1 | peginterferon alfa-2a | Tumour | 27498543 |
| rs760370 | SLC29A1 | peginterferon alfa-2a | Tumour | 20812847 |
| HLA-A*01:01:01:01 | HLA-A | peginterferon alfa-2a | Tumour | 23360626 |
| rs1800797 | IL6 | peginterferon alfa-2a | Tumour | 19387461 |
| rs14158 | LDLR | peginterferon alfa-2a | Tumour | 21572301 |
| rs11854484 | SLC28A2 | peginterferon alfa-2b | Cancer | 26071337 |
| rs17461620 | TUT7 | peginterferon alfa-2b | Cancer | 19749758 |
| rs12819210 | OASL | peginterferon alfa-2b | Cancer | 21993426 |
| HLA-C*14:02:01 | HLA-C | peginterferon alfa-2b | Cancer | 21931540 |
| HLA-C*05:01:01:01 | HLA-C | peginterferon alfa-2b | Cancer | 21931540 |
| rs8113007 | MSRB1P1 | peginterferon alfa-2b | Cancer | 22328925 |
| rs10853728 | MSRB1P1 | peginterferon alfa-2b | Cancer | 21346780 |
| rs304478 | IFIT1 | peginterferon alfa-2b | Cancer | 21993426 |
| TYMS enhancer repeat | TYMS | pemetrexed | Cancer | 11937185;12651279;12972956;14522928;14647408;15713801;15797993;16130010;17203168;17323057;17410198;18322994;18368069;19159907;20386493;21262916;22450926;22838948;25246386 |
| rs3788189, rs1051298 and rs914232 | SLC19A1 | pemetrexed | Cancer | 12571805;19841321;24732178 |
| A2756G | MTR | pemetrexed | Cancer | 11937185;12651279;12972956;14522928;14647408;15713801;15797993;16130010;17203168;17323057;17410198;18322994;18368069;19159907;20386493;21262916;22450926;22838948 |
| rs1051298 | SLC19A1 | pemetrexed | Cancer | 19841321;24732178 |
| rs45445694 | TYMS | pemetrexed | Cancer | 11937185;12651279;12972956;14522928;14647408;15713801;15797993;16130010;17203168;17323057;17410198;18322994;18368069;19159907;20386493;21262916;22450926;22838948 |
| rs3788189 | SLC19A1 | pemetrexed | Cancer | 24732178 |
| rs2236225 | MTHFD1 | pemetrexed | Cancer | 28422153 |
| rs151264360 | TYMS | pemetrexed | Cancer | 25246386 |
| rs11545077 | GGH | pemetrexed | Cancer | 19841321 |
| rs12995526 | ATIC | pemetrexed | Cancer | 25823786 |
| rs442767 | MSH3 | pemetrexed | Cancer | 23709418 |
| rs11615 | ERCC1 | pemetrexed | Cancer | 21262916 |
| rs2952768 | METTL21A | pentazocine | Pain symptom | 23183491;25615449 |
| rs12050217 | BDKRB1 | perindopril | Hypertension | 20712529;27021566 |
| rs12050217 | BDKRB1 | perindopril | Hypertension | 20712529;27021566 |
| rs275651 | AGTR1 | perindopril | Hypertension | 20712529;27021566 |
| G6PD Mediterranean Haplotype | G6PD Mediterranean Haplotype | phenazopyridine | Allergy | 6125724;6410650 |
| HLA-A*24:20 | HLA-A | phenobarbital | Seizures, Epilepsy | 24236482 |
| rs12714145 | GGCX | phenprocoumon | Blood clots, thrombosis, stroke | 21057703;21110013 |
| rs7294 | PRSS53 | phenprocoumon | Blood clots, thrombosis, stroke | 20376629;24224579;25519826 |
| rs510317 | F7 | phenprocoumon | Blood clots, thrombosis, stroke | 21057703;21110013 |
| rs510335 | F7 | phenprocoumon | Blood clots, thrombosis, stroke | 21057703;21110013 |
| rs2359612 | PRSS53 | phenprocoumon | Blood clots, thrombosis, stroke | 19738376 |
| rs10871454 | STX4 | phenprocoumon | Blood clots, thrombosis, stroke | 21063236 |
| rs1799808 | PROC | phenprocoumon | Blood clots, thrombosis, stroke | 21110013 |
| rs3735814 | GATA4 | phenprocoumon | Blood clots, thrombosis, stroke | 23215884 |
| HLA-B*56:02 | HLA-B | phenytoin | Seizures, Epilepsy | 23025739;26928377 |
| CYP2C9*17 | CYP2C9 | phenytoin | Seizures, Epilepsy | 26122019 |
| CYP2C9*41 | CYP2C9 | phenytoin | Seizures, Epilepsy | 27163851 |
| rs71486745 | CYP2C9 | phenytoin | Seizures, Epilepsy | 19855097 |
| rs7900194 | CYP2C9 | phenytoin | Seizures, Epilepsy | 16220110 |
| rs544027339 | CYP2C9 | phenytoin | Seizures, Epilepsy | 26658169 |
| HLA-B*15:13:01 | HLA-B | phenytoin | Seizures, Epilepsy | 26927288 |
| HLA-DRB1*16:02:01 | HLA-DRB1 | phenytoin | Seizures, Epilepsy | 20235791 |
| rs5985 | F13A1 | photodynamic therapy | Cancer | 26307969 |
| rs16861194 | ADIPOQ | pioglitazone | Diabetes | 25405601 |
| rs328 | LPL | pioglitazone | Diabetes | 17394430 |
| rs1110470 | IL4R | pitrakinra | Asthma | 22541248 |
| rs7305534 | AQP2 | platinum | cancer | 24643204;26358256 |
| rs3759125 | AQP2 | platinum | cancer | 24643204;26358256 |
| rs7314734 | AQP2 | platinum | cancer | 24643204;26358256 |
| rs10875989 | AQP2 | platinum | cancer | 24643204;26358256 |
| rs1051685, rs6941 | XRCC5 | platinum | cancer | 26358256 |
| rs7204252, rs4788186, rs4788184, rs1057451 | LRP1 | platinum | cancer | 24643204 |
| rs182455 | STMN1 | platinum | cancer | 26220844 |
| rs7719775 | RICTOR | platinum | cancer | 27676404 |
| rs2748249 | CD74 | platinum | cancer | 24220096 |
| rs1042927 | RRM1 | Platinum compounds | Cancer | 22134350;24361227;28422153 |
| rs1799782 | XRCC1 | Platinum compounds | Cancer | 22821704;24446315;27248474 |
| rs12806698 | STIM1 | Platinum compounds | Cancer | 18483375;18538445;20226083;21642870;22134350;28791697 |
| rs1799801 | ERCC4 | Platinum compounds | Cancer | 25069034;28791697 |
| rs7921977 | CASP7 | Platinum compounds | Cancer | 22441531 |
| rs4979223 | FKBP15 | Platinum compounds | Cancer | 29844858 |
| rs2234767 | FAS | Platinum compounds | Cancer | 22821704 |
| rs1045411 | HMGB1 | Platinum compounds | Cancer | 24684392 |
| rs116907618 | MEG3 | Platinum compounds | Cancer | 26729200 |
| rs1829346 | TUSC7 | Platinum compounds | Cancer | 28814798 |
| rs1130409 | OSGEP | Platinum compounds | Cancer | 28791697 |
| rs2269577 | XBP1 | Platinum compounds | Cancer | 23510626 |
| rs2071554 | HLA-DOB | Platinum compounds | Cancer | 24755914 |
| rs3738948 | ERCC3 | Platinum compounds | Cancer | 25069034 |
| rs2275112 | SFXN4 | Platinum compounds | Cancer | 25732572 |
| rs1127687 | CASP7 | Platinum compounds | Cancer | 22441531 |
| rs1690924 | MDM2 | Platinum compounds | Cancer | 27498158 |
| rs7999812 | ATP7B | Platinum compounds | Cancer | 24852429 |
| rs11030918 | STIM1 | Platinum compounds | Cancer | 22134350 |
| rs12415607 | CASP7 | Platinum compounds | Cancer | 22441531 |
| P2RY12 | P2RY12 | prasugrel | heart attack, stroke | 20938371;24951432 |
| CYP1A2*1A | CYP1A2 | prasugrel | heart attack, stroke | 19414633 |
| rs3737224, rs822442, rs1214331, rs12566888 | PEAR1 | prasugrel | heart attack, stroke | 23859572 |
| CYP1A2*1D | CYP1A2 | prasugrel | heart attack, stroke | 19414633 |
| CYP2C19 extensive metabolizer genotype | CYP2C19 | prasugrel | heart attack, stroke | 20492467 |
| CYP2C19*3A | CYP2C19 | prasugrel | heart attack, stroke | 21689142 |
| rs17655652 | NPC1L1 | pravastatin | High Cholesterol levels | 19752398 |
| rs7102569 | IP6K2 | pravastatin | High Cholesterol levels | 24816038 |
| rs428785 | ADAMTS1 | pravastatin | High Cholesterol levels | 18174457 |
| rs11666735 | FCAR | pravastatin | High Cholesterol levels | 17008591 |
| rs1433099 | LDLR | pravastatin | High Cholesterol levels | 18261733 |
| rs11716445 | RHOA | pravastatin | High Cholesterol levels | 23166513 |
| rs4986791 | TLR4 | pravastatin | High Cholesterol levels | 12742999 |
| rs113646094 | ABCC2 | pravastatin | High Cholesterol levels | 17047488 |
| rs6461639 | RAPGEF5 | prednisone | Inflammation | 26856247 |
| CYP2D6*10B | CYP2D6 | propafenone | Cardiovascular disease | 14653957 |
| rs58597806 | UGT1A10 | propofol | Anesthetic | 18816295 |
| rs6746030 | SCN9A | propofol | Anesthetic | 28611364 |
| Gly16-Glu/Gln27 | ADRB2 3 | propranolol | Hypertension | 26109805 |
| rs2228043 | IL6ST | purine analogues | Cancer | 23570467 |
| rs12746200 | PLA2G4A | Pyrazolones | Pain symptom | 26398624 |
| rs1806201 | GRIN2B | quetiapine | Psychiatric Disorders | 23859574 |
| rs5993883 | COMT | quetiapine | Psychiatric Disorders | 26282453 |
| rs502046, rs10455255 | RIMS1 | quetiapine | Psychiatric Disorders | 19156168 |
| rs264663 | TANC1 | radiotherapy | Cancer | 24974847 |
| rs10711 | CDK1 | radiotherapy | Cancer | 25054431 |
| rs8060157 | ZNF423 | raloxifene | Osteoporosis | 23764426 |
| rs11543791 | AC073114.1 | raloxifene | Osteoporosis | 27546373 |
| UGT1A8*2 | UGT1A8 | raloxifene | Osteoporosis | 23682072 |
| rs10841795 | SLCO1A2 | raltegravir | Viral infection | 23975735 |
| UGT1A9*3a | UGT1A9 | raltegravir | Viral infection | 22371894 |
| rs4359 | ACE | ramipril | Hypertension | 17885551 |
| rs2053044 | ADRB2 | ramipril | Hypertension | 26111150 |
| rs10033900 | CFI | ranibizumab | Hypertension | 23842101 |
| rs3736228 | LRP5 | ranibizumab | Hypertension | 22840423 |
| rs2229935 | NRP1 | ranibizumab | Hypertension | 26426212 |
| rs2283265 | DRD2 | rasagiline | Neurodegenerative disease, Parkinson's Disease | 27190009 |
| G6PD B (wildtype) | G6PD B (wildtype) | rasburicase | Gout | 12075750;12942574;16204390;17387701;18561168;19654083;2019023;20196170;22015451;22190578;22573495;23209099;23860572;23989394;24750455;25115783;25988058;26033222;28370399;29290749;9369411 |
| A- 202A_376G | G6PD | rasburicase | Gout | 12075750;12942574;16204390;17387701;18561168;19654083;2019023;20196170;22015451;22190578;22573495;23209099;23860572;23989394;24750455;25115783;25988058;26033222;28370399;29290749;9369411 |
| G6PD deficiency | G6PD | rasburicase | Gout | 12075750;12942574;16204390;17387701;18561168;19654083;2019023;20196170;20701405;22015451;22190578;22573495;23209099;23860572;23989394;24372186;24750455;24787449;24915143;25115783;25988058;26033222;27099716;27119769;28370399;29290749;9369411 |
| G6PD deficiency | G6PD deficiency | rasburicase | Gout | 12075750;12942574;16204390;17387701;18561168;2019023;22015451;22190578;22573495;23860572;24750455;25115783;25988058;26033222;27099716;27119769;28370399;9369411 |
| G6PD Mediterranean, Dallas, Panama' Sassari, Cagliari, Birmingham | G6PD Mediterranean, Dallas, Panama' Sassari, Cagliari, Birmingham | rasburicase | Gout | 12075750;12942574;16204390;17387701;18561168;19654083;2019023;20196170;22015451;22190578;22573495;23209099;23860572;23989394;24750455;25115783;25988058;26033222;28370399;29290749;9369411 |
| rs5751862 | SPECC1L | regadenoson | cardiovascular disease | 28358597 |
| rs17602729 | AMPD1 | regadenoson | cardiovascular disease | 26554440 |
| rs1470579 | IGF2BP2 | repaglinide | Diabetes | 20523342 |
| rs1801262 | NEUROD1 | repaglinide | Diabetes | 22296034 |
| rs114202595 | PAX4 | repaglinide | Diabetes | 22296034 |
| rs2237892 | KCNQ1 | repaglinide | Diabetes | 22414228 |
| rs16889462 | SLC30A8 | repaglinide | Diabetes | 20809084 |
| rs290487 | TCF7L2 | repaglinide | Diabetes | 20054294 |
| rs2276706 | NR1I2 | repaglinide | Diabetes | 23807564 |
| rs10229583 | FSCN3 | repaglinide | Diabetes | 24752311 |
| rs1800477 | BCL2 | ribavirin | Viral infection | 21159314 |
| rs10946737 | RIPOR2 | rifampin | Infection | 27671213 |
| rs4646536 | CYP27B1 | rifampin | Infection | 28594304 |
| rs4795893,  rs4586 and rs2857657 | CCL2 | risperidone | Psychiatric Disorders | 24495780;26788534 |
| rs724226 | GRM3 | risperidone | Psychiatric Disorders | 19451915;25209194 |
| rs4586 | CCL2 | risperidone | Psychiatric Disorders | 24495780;26788534 |
| rs4795893 | - | risperidone | Psychiatric Disorders | 24495780;26788534 |
| rs4483927 | HRH4 | risperidone | Psychiatric Disorders | 23422377 |
| rs10244329 | LEP | risperidone | Psychiatric Disorders | 23799528 |
| rs9659997 | HTR6 | risperidone | Psychiatric Disorders | 19997080 |
| rs3787430 | HRH3 | risperidone | Psychiatric Disorders | 21652606 |
| rs2494732 | AKT1 | risperidone | Psychiatric Disorders | 18855532 |
| rs167771 | DRD3 | risperidone | Psychiatric Disorders | 19506579 |
| rs9698290 | HTR2C | risperidone | Psychiatric Disorders | 20415561 |
| rs3803300 | ZBTB42 | risperidone | Psychiatric Disorders | 18855532 |
| rs10917670 | - | risperidone | Psychiatric Disorders | 18204343 |
| CYP2D6 ultrarapid metabolizer | CYP2D6 | risperidone | Psychiatric Disorders | 24643635 |
| rs2857657 | CCL2 | risperidone | Psychiatric Disorders | 24495780 |
| rs2069062 | GRM7 | risperidone | Psychiatric Disorders | 26905411 |
| rs724226 | GRM3 | risperidone | Psychiatric Disorders | 19451915 |
| rs4633 | COMT | risperidone | Psychiatric Disorders | 22935916 |
| rs12535512 | ABCB1 | risperidone | Psychiatric Disorders | 27456824 |
| rs10774053 | CACNA1C | ritodrine | psychiatric Disorders | 28391406 |
| rs12488654 | TNFSF10 | rituximab | Rheumatoid arthritis | 27977511 |
| VNTR-243 | TERT | rituximab | Rheumatoid arthritis | 25684018 |
| rs3759467 | TNFSF13B | rituximab | Rheumatoid arthritis | 27780742 |
| rs1081848 | - | rituximab | Rheumatoid arthritis | 22129793 |
| CXCL13 | CXCL13 | rituximab | Rheumatoid arthritis | 26384320 |
| rs3834939 | SLCO1A2 | rocuronium | Anesthesia | 28409297 |
| rs10192566 | LPIN1 | rosiglitazone | Diabetes | 18693052;23426382 |
| Rs10192566 | LPIN1 | rosiglitazone | Diabetes | 18693052;23426382 |
| rs2970847 | PPARGC1A | rosiglitazone | Diabetes | 23426382 |
| SLCO1B1*15 | SLCO1B1 | rosuvastatin | High Cholesterol levels | 16697742;17568401 |
| SLCO1B1*18 | SLCO1B1 | rosuvastatin | High Cholesterol levels | 16697742;17568401 |
| SLCO1B1*1A | SLCO1B1 | rosuvastatin | High Cholesterol levels | 16697742;17568401 |
| SLCO1B1*9 | SLCO1B1 | rosuvastatin | High Cholesterol levels | 16697742;17568401 |
| SLCO1B1*1B | SLCO1B1 | rosuvastatin | High Cholesterol levels | 16697742;17568401 |
| SLCO1B1*5 | SLCO1B1 | rosuvastatin | High Cholesterol levels | 16697742;17568401 |
| APOE E2/E3 | APOE | rosuvastatin | High Cholesterol levels | 17289397 |
| rs1481012 | ABCG2 | rosuvastatin | High Cholesterol levels | 22331829 |
| rs2808630 | CRP | rosuvastatin | High Cholesterol levels | 21094359 |
| Ile796Val | SCAP | rosuvastatin | High Cholesterol levels | 27885915 |
| rs71352238 | NECTIN2 | rosuvastatin | High Cholesterol levels | 22331829 |
| rs4363657 | SLCO1B1 | rosuvastatin | High Cholesterol levels | 23708174 |
| rs17111584 | USP24 | rosuvastatin | High Cholesterol levels | 22331829 |
| rs4149081 | SLCO1B1 | rosuvastatin | High Cholesterol levels | 22668755 |
| rs200707504 | CES1 | sacubitril | cardiovascular disease | 28838926 |
| rs71647871 | CES1 | sacubitril | cardiovascular disease | 26817948 |
| rs295137 | - | salbutamol | Asthma | 22792082;23992748 |
| rs295137 | SPATS2L | salbutamol | Asthma | 22792082;23992748 |
| rs1419555 | AC003975.1 | salbutamol | Asthma | 23508266 |
| rs881152 | DUSP1 | salbutamol | Asthma | 20673984 |
| rs115501901 | SULF2 | salbutamol | Asthma | 23992748 |
| rs892940 | THRB | salbutamol | Asthma | 22212731 |
| rs6500265 | - | salicylamide | Pain symptom | 29239905 |
| rs1540339 | VDR | selective beta-2-adrenoreceptor agonists | Respiratory diseases | 15282200 |
| rs255100 | CRHR2 | selective beta-2-adrenoreceptor agonists | Respiratory diseases | 18408560 |
| rs2284220 | CRHR2 | selective beta-2-adrenoreceptor agonists | Respiratory diseases | 18408560 |
| rs2781659 | ARG1 | selective beta-2-adrenoreceptor agonists | Respiratory diseases | 18617639 |
| rs2781659 | ARG1 | selective beta-2-adrenoreceptor agonists | Respiratory diseases | 18617639 |
| rs6295 | HTR1A | Selective serotonin reuptake inhibitors | Psychiatric Disorders, Major Depression | 18047755;18387740;18484082;19590397;19800133;21688171;23733030 |
| rs1800544 | ADRA2A | Selective serotonin reuptake inhibitors | Psychiatric Disorders, Major Depression | 25642918 |
| rs1843809, rs1386492,rs1487276, rs10897346, rs1487278, rs2171363, rs4570625 | TPH2 | Selective serotonin reuptake inhibitors | Psychiatric Disorders, Major Depression | 24930681 |
| rs10512361 | - | Selective serotonin reuptake inhibitors | Psychiatric Disorders, Major Depression | 25897834 |
| rs1994862 | GRIA1 | Selective serotonin reuptake inhibitors | Psychiatric Disorders, Major Depression | 22534499 |
| rs301434 | SLC1A1 | Selective serotonin reuptake inhibitors | Psychiatric Disorders, Major Depression | 22776887 |
| rs13306278 | TXNRD2 | Selective serotonin reuptake inhibitors | Psychiatric Disorders, Major Depression | 20877297 |
| rs4460839 | DRD2 | Selective serotonin reuptake inhibitors | Psychiatric Disorders, Major Depression | 22796099 |
| rs3800373 | FKBP5 | Selective serotonin reuptake inhibitors | Psychiatric Disorders, Major Depression | 20709156 |
| rs1928040 | HTR2A | Selective serotonin reuptake inhibitors | Psychiatric Disorders, Major Depression | 19937159 |
| rs1994862 | GRIA1 | Selective serotonin reuptake inhibitors | Psychiatric Disorders, Major Depression | 22534499 |
| rs6108160 | PLCB1 | Selective serotonin reuptake inhibitors | Psychiatric Disorders, Major Depression | 25649181 |
| rs140504 | BCR | Selective serotonin reuptake inhibitors | Psychiatric Disorders, Major Depression | 25642918 |
| rs10516436, rs696692, rs5743467, rs2741130, and rs2702877, rs17137566 | TSPAN5, ERICH3, DEFB1, AHR | Selective serotonin reuptake inhibitors | Psychiatric Disorders, Major Depression | 25897834 |
| rs4343 | ACE | sertraline | Psychiatric Disorders, Major Depression | 27262302 |
| rs1137101 | LEPR | simvastatin | High Cholesterol levels | 14625131;18854995 |
| rs3917643 | F3 | simvastatin | High Cholesterol levels | 19027114 |
| rs12052787 | UGT1A6 | simvastatin | High Cholesterol levels | 25493567 |
| SLCO1B1*2 | SLCO1B1 | simvastatin | High Cholesterol levels | 18781850 |
| 3UTR haplotype 5 (rs14158, rs1433099, rs7254521,rs5742911, rs2738467 | LDLRL5 | simvastatin | High Cholesterol levels | 20413733 |
| rs3917643 | F3 | simvastatin | High Cholesterol levels | 19027114 |
| 1784A4G | SREBF2 | simvastatin | High Cholesterol levels | 16158080 |
| CYP3A4*4 | CYP3A4 | simvastatin | High Cholesterol levels | 15650881 |
| rs6924995 | AL021407.1 | simvastatin | High Cholesterol levels | 27071970 |
| UGT2B15*1 | UGT2B15 | sipoglitazar | Diabetes, hypertriglyceridemia | 22960998;23444281 |
| UGT2B15*2 | UGT2B15 | sipoglitazar | Diabetes, hypertriglyceridemia | 22960998;23444281 |
| rs6923761 | GLP1R | sitagliptin | Diabetes | 27160388 |
| UGT1A9*1a | UGT1A9 | SN-38 | Cancer | 24897286 |
| rs35350960 | UGT1A6 | SN-38 | Cancer | 12181437 |
| rs2069502 | CDK4 | somatropin recombinant | Short stature | 23567489 |
| rs2257212 | SLC15A2 | sorafenib | Cancer | 25965825 |
| rs7574296 | UGT1A6 | sorafenib | Cancer | 22736425 |
| rs2239702 | KDR | sorafenib | Cancer | 26830973 |
| rs4604006 | AC093801.1 | sorafenib | Cancer | 24510746 |
| p.Val204Asp | BCHE | succinylcholine | Anesthesia | 25054547 |
| rs761142 | GCLC | sulfamethoxazole | Infection | 22824134 |
| rs7284807 | POLDIP3 | sulfamethoxazole | Infection | 22850190 |
| G6PD deficiency | G6PD | sulfamethoxazole and trimethoprim | Infection | 16388034;18349424;20065266;20732351;3495027;4116253;4120538;4574753;46571;4818663;5061461;751223;990860 |
| HLA-C*06:02:01:01 | HLA-C | sulfamethoxazole and trimethoprim | Infection | 26086150 |
| NAT2*5B | NAT2 | sulfapyridine | Infection | 18167504;20040334 |
| NAT2*6A | NAT2 | sulfapyridine | Infection | 18167504;20040334 |
| NAT2*7B | NAT2 | sulfapyridine | Infection | 18167504;20040334 |
| NAT2*5C | NAT2 | sulfapyridine | Infection | 20040334 |
| HLA-B*15:05:01 | HLA-B | sulfasalazine | Inflammation | 25303297 |
| rs13306673 | SLC12A3 | sulfonamides, plain | Urinary tract infection | 23788015 |
| rs1057910 | CYP2C9 | sulfonamides, urea derivatives | Urinary tract infection | 19794412;29681852 |
| rs7903146 | TCF7L2 | sulfonamides, urea derivatives | Urinary tract infection | 17519421;21114608 |
| rs757110 | ABCC8 | sulfonamides, urea derivatives | Urinary tract infection | 22176616;24442125;29681852 |
| rs5219 | ABCC8 | sulfonamides, urea derivatives | Urinary tract infection | 11318841;16595597;22385882;24442125;25115353;29681852 |
| rs12255372 | TCF7L2 | sulfonamides, urea derivatives | Urinary tract infection | 17519421 |
| rs1799939 | RET | sunitinib | cancer | 19248971;24013576 |
| rs9554320 | FLT1 | sunitinib | cancer | 23421954;27417418 |
| rs1048943 | CYP1A1 | sunitinib | cancer | 19667267;24013576 |
| rs307821 | FLT4 | sunitinib | cancer | 22015057;23462807 |
| rs1126647 | CXCL8 | sunitinib | cancer | 25695485;26387812 |
| rs9582036 | FLT1 | sunitinib | cancer | 23421954;26935927;27417418 |
| rs2276707 | NR1I2 | sunitinib | cancer | 21097692;23462807;24874929 |
| rs307826 | FLT4 | sunitinib | cancer | 22015057;23462807 |
| rs1933437 | FLT3 | sunitinib | cancer | 19667267;20683446;24013576;26244574 |
| ABCB1*2 | ABCB1 | sunitinib | cancer | 12893986; 21097692 |
| rs6785049 | NR1I2 | sunitinib | cancer | 25344452 |
| rs2981582 | FGFR2 | sunitinib | cancer | 23462807 |
| rs34231037 | KDR | sunitinib | cancer | 28430711 |
| rs20541 | IL13 | sunitinib | cancer | 26387812 |
| rs7709359 | FLT4 | sunitinib | cancer | 27896475 |
| rs5744247 | IL18 | tacrolimus | Rheumatoid arthritis | 25712187;28246425 |
| rs11265572 | NR1I3 | tacrolimus | Skin disease | 24351870 |
| rs1927907 | TLR4 | tacrolimus | Skin disease | 24820765 |
| rs2232365 | FOXP3 | tacrolimus | Skin disease | 27747372 |
| rs6267 | COMT | tacrolimus | Skin disease | 24465960 |
| rs846908 | HSD11B1 | tacrolimus | Skin disease | 25587129 |
| rs4646458 | CYP3A5 | tacrolimus | Skin disease | 21047202 |
| rs9200 | C6 | tacrolimus | Skin disease | 28685716 |
| rs237025 | SUMO4 | tacrolimus | Skin disease | 28941036 |
| rs181781 | IL3 | tacrolimus | Skin disease | 28112181 |
| rs1800822 | FMO3 | tacrolimus | Skin disease | 28084894 |
| rs3730251 | PPP3CA | tacrolimus | Skin disease | 28435308 |
| rs1851426 | CYP3A4 | tacrolimus | Skin disease | 22108237 |
| rs5030952 | CAPN10 | tacrolimus | Skin disease | 19752882 |
| rs2307418 | NR1I3 | tacrolimus | Skin disease | 28777242 |
| rs17143212 | ABCB5 | talinolol | Hypertension, cardiovascular diseases | 27825374 |
| rs17143212 | ABCB5 | talinolol | Hypertension, cardiovascular diseases | 27825374 |
| SULT1A1*1 | SULT1A1*1 | tamoxifen | Cancer, Breast | 12419790;15987423;17244352 |
| SULT1A1*2 | SULT1A1*2 | tamoxifen | Cancer, Breast | 12419790;15987423;17244352 |
| P1272S | SRC1 | tamoxifen | Cancer, Breast | 22174377 |
| CCNA2 | CCNA2 | tamoxifen | Cancer, Breast | 24622579 |
| rs2016347 | IGF1R | tamoxifen | Cancer, Breast | 23459444 |
| rs10509373 | LRMDA | tamoxifen | Cancer, Breast | 22180457 |
| rs478437 | E2F7 | tamoxifen | Cancer, Breast | 23508821 |
| rs10509373 | C10orf11 | tamoxifen | Cancer, Breast | 22180457 |
| rs11023197 | RRAS2 | tamoxifen | Cancer, Breast | 19047159 |
| CYP2D6*6B | CYP2D6 | tamoxifen | Cancer, Breast | 24060820 |
| rs2011425 | UGT1A6 | tamoxifen-n-glucuronide | Cancer | 27098059 |
| rs9369421 | - | taxanes | Cancer | 26194361 |
| rs111033610 | CYP2A6 | tegafur | Cancer | 12042667 |
| rs8192720 | CYP2A6 | tegafur | Cancer | 21521021 |
| rs2308321 | MGMT | temozolomide | Cancer | 20938339;24444404 |
| rs2308321 | MGMT | temozolomide | Cancer | 20938339 |
| rs1045642 | ABCB1 | tenofovir | Viral infection | 17083032;19400747 |
| rs3742106 | ABCC4 | tenofovir | Viral infection | 19400747;25801567 |
| rs1128503 | ABCB1 | tenofovir | Viral infection | 17083032;19400747 |
| rs717620 | ABCC2 | tenofovir | Viral infection | 17083032;19400747;21288825;25801567;26287941;26535588;26807589 |
| 1249 G>A | ABCC2 | tenofovir | Viral infection | 17083032;19400747;21288825;25485598;25801567;26287941;26535588;26807589 |
| rs2273697 | ABCC2 | tenofovir | Viral infection | 17083032;19400747;21288825;25485598;25801567;26535588;26807589 |
| rs1751034 | ABCC4 | tenofovir | Viral infection | 17083032;17597712;18398970;19400747;25801567 |
| rs17222723 | ABCC2 | tenofovir | Viral infection | 17083032;19400747 |
| rs8187710 | ABCC2 | tenofovir | Viral infection | 17083032;19400747;25485598 |
| rs899494 | ABCC4 | tenofovir | Viral infection | 17083032;19400747;21288825;25485598 |
| rs3740066 | ABCC2 | tenofovir | Viral infection | 19400747;25801567 |
| rs2274407 | ABCC4 | tenofovir | Viral infection | 17083032 |
| rs11568694 | OCRL | tenofovir | Viral infection | 25485598 |
| rs2125739 | ABCC10 | tenofovir | Viral infection | 21628669 |
| rs11568658 | ABCC4 | tenofovir | Viral infection | 17083032 |
| rs1059751 | ABCC4 | tenofovir | Viral infection | 26807589 |
| rs11231809 | SLC22A11 | tenofovir | Viral infection | 19400747 |
| rs2032582 | ABCB1 | tenofovir | Viral infection | 26535588 |
| rs2231142 | ABCG2 | tenofovir | Viral infection | 28462920 |
| rs11568695 | ABCC4 | tenofovir | Viral infection | 17083032 |
| rs4341 | ACE | Thiazides, plain | Hypertension | 19247266 |
| rs113681054 | - | ticagrelor | heart attack, stroke | 25935875;28049954 |
| rs6785930 | MED12L | ticagrelor | heart attack, stroke | 26083990 |
| rs4661012 | PEAR1 | ticagrelor | heart attack, stroke | 27937053 |
| rs2242480 | CYP3A4 | ticagrelor | heart attack, stroke | 28049954 |
| rs3093135 | CYP4F2 | ticagrelor | heart attack, stroke | 28604225 |
| rs56324128 | CYP3A4 | ticagrelor | heart attack, stroke | 25935875 |
| rs4329505 | IL6R | tocilizumab | Rheumatoid arthritis | 24978393;27958380 |
| rs12083537 | IL6R | tocilizumab | Rheumatoid arthritis | 24978393;27958380 |
| rs11265618 | IL6R | tocilizumab | Rheumatoid arthritis | 27958380 |
| rs9332239 | CYP2C9 | tolbutamide | Blood glucose lowering agent | 15284535 |
| rs2832407 | GRIK1 | topiramate | Seizures, Epilepsy | 19331489 |
| rs2396185 | INSR | topiramate | Seizures, Epilepsy | 26524290 |
| rs2832407 | GRIK1 | topiramate | Seizures, Epilepsy | 19331489 |
| rs4984241 | CA12 | topiramate | Seizures, Epilepsy | 21278619 |
| rs12346562 and rs1104514, rs4742610 | PTPRD | trandolapril | Hypertension | 26425837 |
| rs121907896, rs121907892 | SLC22A12 | tranilast | Inflammation, gout, hyperuricemia | 24915143 |
| rs2523864, rs3873352 | HCG22 | triamcinolone | Inflammation | 25813999 |
| HLA-B*13:01:01 | HLA-B | trichloroethylene | Anesthetics | 18007983 |
| rs10811661 | - | troglitazone | Blood glucose lowering agent | 18544707 |
| rs4696480 | AC106865.1 | Tumor necrosis factor alpha (TNF-alpha) inhibitors | Inflammation, arthritis | 24776844;28696418 |
| rs352139 | TWF2 | Tumor necrosis factor alpha (TNF-alpha) inhibitors | Inflammation, arthritis | 24776844;28696418 |
| rs11938228 | TLR2 | Tumor necrosis factor alpha (TNF-alpha) inhibitors | Inflammation, arthritis | 24776844;28696418 |
| rs1813443 | CNTN5 | Tumor necrosis factor alpha (TNF-alpha) inhibitors | Inflammation, arthritis | 23233654 |
| rs854548 | PON1 | Tumor necrosis factor alpha (TNF-alpha) inhibitors | Inflammation, arthritis | 18615156 |
| rs9304742 | ZNF816 | Tumor necrosis factor alpha (TNF-alpha) inhibitors | Inflammation, arthritis | 27670765 |
| rs11465996 | LY96 | Tumor necrosis factor alpha (TNF-alpha) inhibitors | Inflammation, arthritis | 28696418 |
| rs651630 | SLC12A8 | Tumor necrosis factor alpha (TNF-alpha) inhibitors | Inflammation, arthritis | 26194362 |
| rs10945919 | AL078602.1 | Tumor necrosis factor alpha (TNF-alpha) inhibitors | Inflammation, arthritis | 18615156 |
| rs11591741 | CHUK | Tumor necrosis factor alpha (TNF-alpha) inhibitors | Inflammation, arthritis | 25896535 |
| rs1799964 | LTA | Tumor necrosis factor alpha (TNF-alpha) inhibitors | Inflammation, arthritis | 23057546 |
| rs1975974 | C17orf51 | ustekinumab | Skin disease | 27977334 |
| PTGS2 | PTGS2 | valdecoxib | Inflammation | 19602986 |
| rs11568658 | SLC29A1 | valganciclovir | Viral infection | 27402191 |
| rs1731017 | ABAT | valproic acid | Seizures, Epilepsy | 23407051;27918244 |
| UGT1A6*3a | UGT1A6 | valproic acid | Seizures, Epilepsy | 23099353 |
| rs2307441 | POLG | valproic acid | Seizures, Epilepsy | 21038416 |
| rs3816877 | APEH | valproic acid | Seizures, Epilepsy | 27406852 |
| rs226957 | XBP1 | valproic acid | Seizures, Epilepsy | 23407051 |
| rs28898617 | UGT1A6 | valproic acid | Seizures, Epilepsy | 28763744 |
| rs6731242 | UGT1A10 | valproic acid | Seizures, Epilepsy | 21806385 |
| rs1799883 | FABP2 | valproic acid | Seizures, Epilepsy | 21806385 |
| rs1731017 | ABAT | valproic acid | Seizures, Epilepsy | 27918244 |
| rs10070381 | - | vancomycin | Infection | 26030142 |
| rs776746 | CYP3A5 | vardenafil | erectile dysfunction | 21934637 |
| rs2072660 | CHRNB2 | varenicline | Smoking cessation | 21606948;25774163 |
| rs1044396 | CHRNB2 | varenicline | Smoking cessation | 21606948;25774163 |
| rs2072661 | CHRNB2 | varenicline | Smoking cessation | 21606948;25774163 |
| rs2236196 | CHRNA4 | varenicline | Smoking cessation | 25774163 |
| rs4292956 | CHRNB2 | varenicline | Smoking cessation | 21606948 |
| CYP2A6 extensive metabolizer | CYP2A6 | varenicline | Smoking cessation | 25588294 |
| rs578776 | CHRNA5 | varenicline | Smoking cessation | 26010901 |
| rs28363170 | SLC6A3 | venlafaxine | Psychiatric Disorders, Major Depression | 24723432 |
| rs2274639 | HTR2A | venlafaxine | Psychiatric Disorders, Major Depression | 28068779 |
| rs3810651 | GABRQ | venlafaxine | Psychiatric Disorders, Major Depression | 23394390 |
| rs11739136 | KCNIP1 | verapamil | Hypertension | 17700361;18496125;18854753 |
| rs11739136 | KCNMB1 | verapamil | Hypertension | 17700361;18496125;18854753 |
| rs11739136 | KCNMB1 | verapamil | Hypertension | 17700361;18496125;18854753 |
| rs2357928 | CACNB2 | verapamil | Hypertension | 21156931 |
| rs2301149 | KCNMB1 | verapamil | Hypertension | 17700361 |
| rs10918594 | - | verapamil | Hypertension | 19247217 |
| rs12221497 | NR1H3 | verapamil | Hypertension | 21562465 |
| rs924607 | CEP72 | vincristine | Cancer | 25710658;25999454;27618250 |
| rs924607 | CEP72 | vincristine | Cancer | 25710658;26618658;27618250 |
| CYP3A5*3C | CYP3A5 | vincristine | Cancer | 21225912;21404110 |
| rs3770102 | CAPG | vincristine | Cancer | 25084203 |
| rs1801133 | MTHFR | vitamin b-complex, plain | Diet Supplement | 22926161 |
| rs9409929 | - | vitamin d and analogues | Diet Supplement | 20307661 |
| rs3093105 | CYP4F2 | vitamin e | Inflammation | 20861217;24759732 |
| rs2108622 | CYP4F2 | Vitamin K | Blood clots, thrombosis, stroke | 19297519;28521079 |
| rs193922832 | RYR1 | volatile anesthetics | Anesthesia | 19648156;28403410 |
| rs193922764 | RYR1 | volatile anesthetics | Anesthesia | 12059893;16163667;24433488;25735680 |
| rs193922843 | RYR1 | volatile anesthetics | Anesthesia | 19648156;28403410 |
| CYP2C19 ultrarapid metabolizer | CYP2C19 | voriconazole | Infection | 25558073;28923870 |
| CYP2C19 poor metabolizers and intermediate metabolizers | CYP2C19 | voriconazole | Infection | 19261446;27388292;28923870 |
| CYP2C19 intermediate metabolizers | CYP2C19 | voriconazole | Infection | 25239277;25558073;27388292 |
| CYP3A5*3A | CYP3A5 | voriconazole | Infection | 19033450 |
| CYP2C19*1 | CYP2C19 | voriconazole | Infection | 27981572 |
| CYP2C19 poor metabolizer genotype | CYP2C19 | voriconazole | Infection | 15179414 |
| CYP2C9 poor metabolizer and intermediate metabolizer genotypes | CYP2C9 | voriconazole | Infection | 17827141 |
| CYP2C19*2A | CYP2C19 | voriconazole | Infection | 25084200 |
| CYP2C19 intermediate metabolizer | CYP2C19 | voriconazole | Infection | 25558073 |
| rs4646437 | CYP3A4 | voriconazole | Infection | 25515945 |
| CYP3A5 poor metabolizer genotype | CYP3A5 | voriconazole | Infection | 17827141 |
| rs4841588 | GATA4 | warfarin | Blood clots, thrombosis, stroke | 25026456;29298995 |
| rs56165452 | CYP2C9 | warfarin | Blood clots, thrombosis, stroke | 17391071;18542936;19387626;21228733 |
| rs61162043 | BCKDK | warfarin | Blood clots, thrombosis, stroke | 21270790;22158446 |
| rs4889606 | STX1B | warfarin | Blood clots, thrombosis, stroke | 26751406;28135054 |
| rs2645400 | GATA4 | warfarin | Blood clots, thrombosis, stroke | 25026456;29298995 |
| rs4653436 | EPHX1 | warfarin | Blood clots, thrombosis, stroke | 19794411;21593757;25126975 |
| rs5896 | F2 | warfarin | Blood clots, thrombosis, stroke | 14656880;15590403;21320153 |
| rs7196161 | VKORC1 | warfarin | Blood clots, thrombosis, stroke | 21383771;24019055;25519826 |
| VKORC1*3 | VKORC1 | warfarin | Blood clots, thrombosis, stroke | 21127708;22676192;25084205 |
| rs2901783 | CYP2C18 | warfarin | Blood clots, thrombosis, stroke | 19752777;23327576;28686080 |
| rs339097 | CALU | warfarin | Blood clots, thrombosis, stroke | 20200517;21228733;25126975 |
| VKORC1*4 | VKORC1 | warfarin | Blood clots, thrombosis, stroke | 21127708;22676192;25084205 |
| rs104894541 | VKORC1 | warfarin | Blood clots, thrombosis, stroke | 14765194;18234403 |
| rs104894540 | VKORC1 | warfarin | Blood clots, thrombosis, stroke | 14765194;18234403 |
| rs2290228 | CALU | warfarin | Blood clots, thrombosis, stroke | 20128861;22549502;25126975 |
| rs1877724 | EPHX1 | warfarin | Blood clots, thrombosis, stroke | 21192345;29054760 |
| rs61742245 | VKORC1 | warfarin | Blood clots, thrombosis, stroke | 17110455;20128861;22266406;22871975;23571513 |
| rs12777823 | CTBP2P2 | warfarin | Blood clots, thrombosis, stroke | 23755828;25461246;26024874;26877068;28686080 |
| rs8050894 | PRSS53 | warfarin | Blood clots, thrombosis, stroke | 16270629;16493479;16611750;17456829;18030307;18466099;19874474;20203262;24019055 |
| VKORC1*1 | VKORC1 | warfarin | Blood clots, thrombosis, stroke | 18322281;20354686 |
| rs699664 | GGCX | warfarin | Blood clots, thrombosis, stroke | 17049586;19582440;21326313;22549502;22676192;23941071;25126975;25594941;25681132;26106580;26257249;27262824;27632229;28049362;28429387 |
| rs104894542 | PRSS53 | warfarin | Blood clots, thrombosis, stroke | 14765194;18234403 |
| rs4917639 | CYP2C9 | warfarin | Blood clots, thrombosis, stroke | 18535201;18574025;25126975 |
| rs2884737 | VKORC1 | warfarin | Blood clots, thrombosis, stroke | 16611750;18030307;20072124;24019055 |
| CYP4F2*1 | CYP4F2 | warfarin | Blood clots, thrombosis, stroke | 21174619;24085937;24503627;26745506 |
| rs7856096 | FPGS | warfarin | Blood clots, thrombosis, stroke | 25079360;26751406 |
| rs7089580 | CYP2C9 | warfarin | Blood clots, thrombosis, stroke | 21270790;24474498;25499099 |
| rs72558189 | CYP2C9 | warfarin | Blood clots, thrombosis, stroke | 24911077;27121899 |
| VKORC1 H7 | VKORC1 | warfarin | Blood clots, thrombosis, stroke | 17387222;17391071 |
| rs17708472 | VKORC1 | warfarin | Blood clots, thrombosis, stroke | 16270629;16611750;20128861;21127708;22676192;25084205 |
| rs9332127 | CYP2C9 | warfarin | Blood clots, thrombosis, stroke | 22248286;25126975 |
| VKORC1 H1 | VKORC1 | warfarin | Blood clots, thrombosis, stroke | 17387222;19745563 |
| rs72547529 | PRSS53 | warfarin | Blood clots, thrombosis, stroke | 15630486;19663669;20615525;26513304 |
| rs2901783 | CYP2C18 | warfarin | Blood clots, thrombosis, stroke | 19752777;23327576 |
| rs11676382 | GGCX | warfarin | Blood clots, thrombosis, stroke | 17764537;20128861;20694283;24019055;25042728;25681132;27632229;28049362;29432897 |
| CYP4F2*3 | CYP4F2 | warfarin | Blood clots, thrombosis, stroke | 21174619;24085937;24503627;26710337;26745506 |
| VKORC1*2 | VKORC1 | warfarin | Blood clots, thrombosis, stroke | 18322281;20354686;21127708 |
| rs17886199 | VKORC1 | warfarin | Blood clots, thrombosis, stroke | 15358623;16270629;16611750;17049586;17329985;20716240 |
| rs17880887 | VKORC1 | warfarin | Blood clots, thrombosis, stroke | 20072124;21179214 |
| rs104894539 | VKORC1 | warfarin | Blood clots, thrombosis, stroke | 14765194;18234403 |
| rs17650 | ORM1 | warfarin | Blood clots, thrombosis, stroke | 19794411;23208322 |
| rs2260863 | EPHX1 | warfarin | Blood clots, thrombosis, stroke | 19794411;28079798 |
| rs2189784 | CLEC4O | warfarin | Blood clots, thrombosis, stroke | 19741565;20128861;28079798;28620303 |
| rs4889606 | STX1B | warfarin | Blood clots, thrombosis, stroke | 26751406;27740732;28135054 |
| CYP2C9*9 | CYP2C9 | warfarin | Blood clots, thrombosis, stroke | 21590310 |
| rs10509680 | CYP2C9 | warfarin | Blood clots, thrombosis, stroke | 20833655 |
| VKORC1L1 | VKORC1 | warfarin | Blood clots, thrombosis, stroke | 29054760 |
| rs17650 | ORM1 | warfarin | Blood clots, thrombosis, stroke | 23208322 |
| CYP2C9*58 | CYP2C9 | warfarin | Blood clots, thrombosis, stroke | 25075423 |
| rs3756009 | F11 | warfarin | Blood clots, thrombosis, stroke | 25126975 |
| rs7200749 | PRSS53 | warfarin | Blood clots, thrombosis, stroke | 21635147 |
| rs10239977 | POR | warfarin | Blood clots, thrombosis, stroke | 27488389 |
| rs2304429 | DNMT3A | warfarin | Blood clots, thrombosis, stroke | 27740732 |
| rs142410335 | MIR133B | warfarin | Blood clots, thrombosis, stroke | 28429387 |
| CYP2C9*4 | CYP2C9 | warfarin | Blood clots, thrombosis, stroke | 17391071 |
| rs11653 | CALU | warfarin | Blood clots, thrombosis, stroke | 19794411 |
| CYP2C9*10 | CYP2C9 | warfarin | Blood clots, thrombosis, stroke | 19802360 |
| rs1051741 | EPHX1 | warfarin | Blood clots, thrombosis, stroke | 20716240 |
| rs3212198 | HNF4A | warfarin | Blood clots, thrombosis, stroke | 25356900 |
| rs4645974 | MYC | warfarin | Blood clots, thrombosis, stroke | 26249541 |
| rs2288344 | NEDD4 | warfarin | Blood clots, thrombosis, stroke | 27488176 |
| rs11150606 | PRSS53 | warfarin | Blood clots, thrombosis, stroke | 15883587 |
| rs216013 | CACNA1C | warfarin | Blood clots, thrombosis, stroke | 18535201 |
| rs2592551 | GGCX | warfarin | Blood clots, thrombosis, stroke | 24148610 |
| rs4760658 | AC121338.1 | warfarin | Blood clots, thrombosis, stroke | 29298995 |
| VKORC1 H8 | VKORC1 | warfarin | Blood clots, thrombosis, stroke | 17387222 |
| rs2917677 | NQO1 | warfarin | Blood clots, thrombosis, stroke | 26257249 |
| rs2860905 | CYP2C9 | warfarin | Blood clots, thrombosis, stroke | 19752777 |
| rs11636419 | CYP1A2 | warfarin | Blood clots, thrombosis, stroke | 22248286 |
| rs17126068 | DDHD1 | warfarin | Blood clots, thrombosis, stroke | 27488176 |
| rs112936952 | GGCX | warfarin | Blood clots, thrombosis, stroke | 19582440 |
| CYP2C9*57 | CYP2C9 | warfarin | Blood clots, thrombosis, stroke | 23582453 |
| rs9332238 | CYP2C9 | warfarin | Blood clots, thrombosis, stroke | 26265036 |
| rs12772169 | CTBP2P2 | warfarin | Blood clots, thrombosis, stroke | 28686080 |
| rs6046 | F7 | warfarin | Blood clots, thrombosis, stroke | 22071881 |
| rs9332098 | CYP2C9 | warfarin | Blood clots, thrombosis, stroke | 21326313 |
| rs1060467 | CYP4F11 | warfarin | Blood clots, thrombosis, stroke | 28620303 |
| rs10931910 | AHR | XK469 | Tumour | 24300566 |
| rs11568695 | ABCC4 | zidovudine | Viral infection | 22960662 |
| rs286913 | DTYMK | ziprasidone | Psychiatric Disorders | 21107309 |
| rs1934951 | EHF | zoledronate | cancer | 18594024;21151627 |
| rs1934951, rs1934980, rs1341162, and rs17110453 | CYP2C8 | zoledronate | cancer | 18594024;21151627 |
| rs1152003 | PPARG | zoledronate | cancer | 21517810 |
| BsmI, ApaI, and TaqI | VDR | zoledronate | cancer | 18443790 |
| rs2297480 | FDPS | zoledronate | cancer | 21196316 |

**Supplementary Table 11:** A comprehensive table of 2,304 human PGx relationships between genetic variant- gene- drug-disease with their corresponding evidences available. The genetic variants are annotated as in the articles presented as evidences. In certain cases when no genetic variant in particular was found, but the gene itself is referred as a biomarker, the gene names are mentioned both in genetic variant and in the gene column. The disease column contains the diseases as mentioned in the evidence articles, if not, then diseases for which the drugs are commonly prescribed are mentioned.

| **Disease category** | **Disease** | **Drugs** | **Genes** | **Frequency** |
| --- | --- | --- | --- | --- |
| Cardiovascular | Analgesic | Tramadol | CYP2D6 | 33 |
|  | Congestive heart failure | Carvedilol | CYP2D6 | 44 |
|  | Heart attack and stroke | Clopidogrel | CYP2C19 | 140 |
|  | Coronary disease | Prasugrel | CYP2C19 | 21 |
|  |  | Prasugrel | CYP2C9 | 3 |
|  |  | Prasugrel | CYP3A5 | 3 |
|  |  | Prasugrel | CYP2B6 | 3 |
|  | Prolonged QT interval | Quinidine | CYP2D6 | 73 |
|  | Coronary disease | Ticagrelor | CYP2C19 | 15 |
|  | Skin cancer | Fluorouracil | DPYD | 1779 |
|  | Stomach ulcer | Esomeprazole | CYP2C19 | 14 |
|  | Gastroesophageal reflux disease | Lansoprazole | CYP2C19 | 26 |
|  |  | Omeprazole | CYP2C19 | 137 |
|  |  | Pantoprazole | CYP2C19 | 15 |
|  |  | Rabeprazole | CYP2C19 | 26 |
|  | Heart disease | Warfarin | CYP2C9 | 431 |
|  |  | Warfarin | VKORC1 | 359 |
|  |  | Warfarin | PROC | 6 |
| Infection | HIV | Abacavir | HLA-B | 97 |
|  |  | Dolutegravir | UGT1A1 | 22 |
|  |  | Efavirenz | CYP2B6 | 138 |
|  | Leprosy | Dapsone | G6PD | 30 |
|  | Hepatitis C | Dasabuvir | IFNL3 | 4 |
|  | Malaria | Primaquine | G6PD | 21 |
| Neurological | Epilepsy | Carbamazepine | HLA-B | 62 |
|  |  | Carbamazepine | HLA-A | 32 |
|  |  | Clobazam | CYP2C19 | 10 |
|  |  | Oxcarbazepine | HLA-B | 27 |
|  |  | Phenytoin | CYP2C9 | 55 |
|  |  | Phenytoin | CYP2C19 | 67 |
|  |  | Phenytoin | HLA-B | 14 |
|  |  | Valproic Acid | POLG | 478 |
|  | Huntington's disease | Tetrabenazine | CYP2D6 | 4 |
|  | PseudoBulbar Affect | Dextromethorphan and Quinidine | CYP2D6 | 78 |
| Cancer | Lung cancer | Afatinib | EGFR | 67 |
|  |  | Ceritinib | ALK | 29 |
|  | Leukemia | Bosutinib | BCR-ABL1 | 13 |
|  | Colorectal cancer | Irinotecan | UGT1A1 | 502 |
|  |  | Cetuximab | EGFR | 20 |
|  | Cervical cancer | Cisplatin | TPMT | 3324 |
|  | Non-small cell lung cancer | Erlotinib | EGFR | 604 |
|  |  | Gefitinib | EGFR | 794 |
|  | Chronic myelogenous leukemia | Dasatinib | BCR-ABL1 | 221 |
|  |  | Imatinib | KIT | 242 |
|  |  | Imatinib | BCR-ABL1 | 14 |
|  |  | Imatinib | PDGFRB | 57 |
|  |  | Imatinib | FIP1L1-PDGFRA | 52 |
|  | Breast cancer | Lapatinib | ERBB2 | 41 |
|  |  | Lapatinib | HLA-DQA1, HLA-DRB1 | 4 |
|  |  | Tamoxifen | ESR, PGR | 56 |
|  |  | Trametinib | RAS | 55 |
| Psychiatry | Major depressive disorder | Citalopram | CYP2C19 | 30 |
|  |  | Citalopram | CYP2D6 | 18 |
|  |  | Clomipramine | CYP2D6 | 19 |
|  |  | Desipramine | CYP2D6 | 34 |
|  |  | Desvenlafaxine | CYP2D6 | 6 |
|  |  | Escitalopram | CYP2D6 | 6 |
|  |  | Escitalopram | CYP2C19 | 13 |
|  |  | Fluoxetine | CYP2D6 | 127 |
|  |  | Imipramine | CYP2D6 | 26 |
|  |  | Paroxetine | CYP2D6 | 123 |
|  |  | Venlafaxine | CYP2D6 | 67 |
|  | Schizophrenia | Clozapine | CYP2D6 | 275 |
|  |  | Risperidone | CYP2D6 | 219 |

**Additional table for readers (not cited in article):** **PGx markers common between FDA approved drug labels available and proposed pipeline**
